# Design, synthesis, and anticancer evaluation of novel quinoline derivatives of ursolic acid with hydrazide, oxadiazole, and thiadiazole moieties as potent MEK inhibitors

**DOI:** 10.1080/14756366.2019.1605364

**Published:** 2019-05-09

**Authors:** Xiao-Yan Jin, Hao Chen, Dong-Dong Li, A-Liang Li, Wen-Yan Wang, Wen Gu

**Affiliations:** Jiangsu Provincial Key Lab for the Chemistry and Utilization of Agro-forest Biomass, Jiangsu Key Lab of Biomass-Based Green Fuels and Chemicals, Co-Innovation Center for Efficient Processing and Utilization of Forest Products, College of Chemical Engineering, Nanjing Forestry University, Nanjing, PR China

**Keywords:** Ursolic acid, quinoline, antiproliferative activity, MEK inhibitor, apoptosis

## Abstract

In this article, a series of novel quinoline derivatives of ursolic acid (UA) bearing hydrazide, oxadiazole, or thiadiazole moieties were designed, synthesised, and screened for their *in vitro* antiproliferative activities against three cancer cell lines (MDA-MB-231, HeLa, and SMMC-7721). A number of compounds showed significant activity against at least one cell line. Among them, compound **4d** exhibited the most potent activity against three cancer cell lines with IC_50_ values of 0.12 ± 0.01, 0.08 ± 0.01, and 0.34 ± 0.03 μM, respectively. In particular, compound **4d** could induce the apoptosis of HeLa cells, arrest cell cycle at the G0/G1 phase, elevate intracellular reactive oxygen species level, and decrease mitochondrial membrane potential. In addition, compound **4d** could significantly inhibit MEK1 kinase activity and impede Ras/Raf/MEK/ERK transduction pathway. Therefore, compound **4d** may be a potential anticancer agent and a promising lead worthy of further investigation.

## GRAPHICAL ABSTRACT

**Figure:**
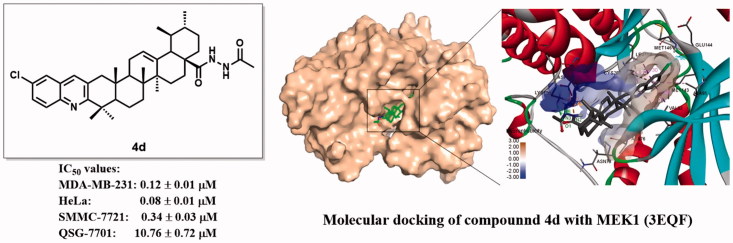

## 1. Introduction

Nowadays, cancer has still been the leading cause of human death worldwide and has become a major public health problem. It is estimated that there were 18 million new cases of cancer and 9.6 million cancer-related deaths occurred in 2018 according to Global Cancer Statistics 2018^1^. Despite the indispensable role in the treatment of cancer in the past few decades, traditional cancer chemotherapies often cause less satisfactory results due to severe side effects and rapidly occurred drug-resistance^2^. Therefore, there still has been an overwhelming need in searching for and developing new anticancer drugs with better efficacy and toxicity profiles.

**Figure 1.**
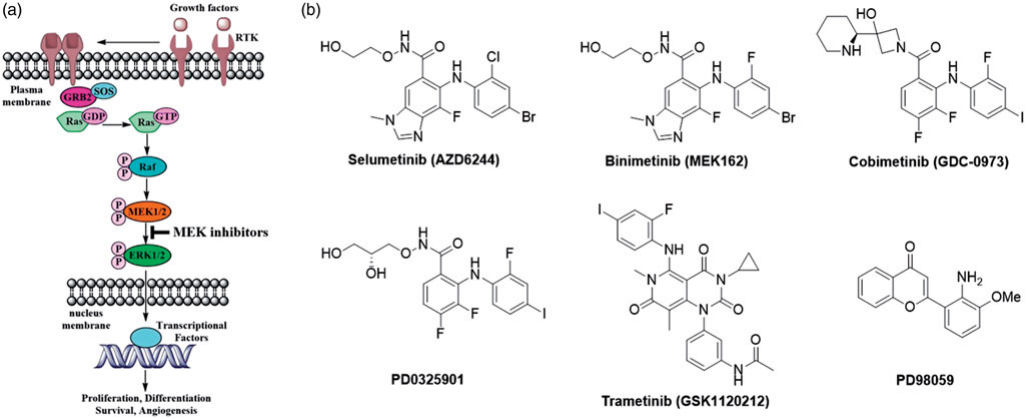
(a) A concise diagram of MAPK signalling pathway. (b) Examples of MEK kinase inhibitors.

In recent years, a number of key signalling pathways, membrane receptors, kinases, transcriptional factors, and other biological macromolecules have been identified, which results in further understanding on the pathogenesis of cancer^3^. Development of small molecular drugs specially targeting the key proteins in the signalling pathways relevant to tumourigenesis and tumour growth may provide opportunities to find new targeted anticancer agents with significantly improved therapeutic index. The mitogen-activated protein kinase (MAPK) signalling pathway is one of the most important signalling pathways correlated with targeted cancer therapeutics^4^^,^^5^. Rat sarcoma small GTPase/rapidly accelerated fibrosarcoma kinase/ MAPK/extracellular signal-regulated kinase (Ras/Raf/MEK/ERK) signalling pathway (i.e. MAPK pathway) transduces signals from cell surface receptors to the nucleus through a series of phosphorylation events (Figure 1(a)). Once a growth factor binds to its respective receptor, the signalling pathway is activated. The receptor tyrosine kinase (RTK) transmits the extracellular signal into the cell *via* the adaptor protein GRB2 which subsequently activates the membrane-bound GTPase (Ras) with the help of nucleotide exchange factor SOS. Activated Ras then initiates the signalling cascade of MAP kinases. Finally, activated ERK can phosphorylate its cytosolic or nuclear effectors. The latter results in changes to transcription and ultimately promotes many cellular processes including proliferation, differentiation, survival, and angiogenesis^6–8^. The dysregulation of MAPK pathway is strongly associated with many human cancers, such as hepatocellular carcinoma (HCC)^9^, non-small cell lung cancer (NSCLC)^10^, cervical carcinoma^11^, prostate carcinoma^12^, and melanoma^13^. Therefore, the signalling pathway offers attractive targets for the development of anticancer agents.

Among this signalling pathway, the Ser/Thr kinases MEK1/2 have been attracting special interests because the kinases specifically phosphorylate and activate ERK1/2. The activated ERK translocates to the nucleus where it phosphorylates a variety of transcription factors regulating gene expression^14^. Hence, the interest in MEK1/2 has generated several small molecule MEK inhibitors, such as Selumetinib (AZD6244), binimetinib (MEK162), cobimetinib (GDC-0973), Trametinib (GSK1120212), PD0325901, and PD98059 (Figure 1(b))^7^^,^^15^. Selumetinib (AZD6244), a potent selective MEK1/2 inhibitor was reported in 2008 and showed significant improvements in patients with advanced cancers. The compound was granted orphan drug status by FDA in May 2016 for the treatment of patients with stage III or IV differentiated thyroid cancer^15^. Selumetinib has also been extensively studied for the treatment of KRAS-mutated NSCLC. A 2013 Phase II study demonstrated that the combination of selumetinib and docetaxel acted synergistically, leading to an improved OS and PFS compared to placebo and docetaxel^16^. However, disappointing data emerged from the next phase III trial in which the addition of selumetinib to docetaxel in patients with advanced KRAS-mutant NSCLC did not improve survival or show clinical benefit^17^. Moreover, binimetinib (MEK162) was endorsed as a contemporary MEK1/2 inhibitor, which has been recently approved by FDA for the treatment of BRAF-mutant melanoma in combination with encorafenib based on a phase III clinical trial (COLUMBUS)^18^. Trametinib (GSK1120212, MEKINIST^®^) is also a potent MEK1/2 inhibitor, which showed good inhibition to metastatic melanoma carrying BRAF^V600E^ mutation. FDA recently approved the combination of dabrafenib and trametinib for the treatment of BRAF-mutant metastatic melanoma, NSCLC, and anaplastic thyroid cancer^19^. These examples have highlighted the potential of MEK inhibitors as novel anticancer agents. However, discovery of more MEK-targeting lead inhibitors with better efficacy and tolerability is still an urgent therapeutic need.

Terpenoids represent a large class of natural products consisting of approximately 25,000 chemical structures, which are widely applied in the fragrance and flavour industries. Furthermore, terpenoids exhibit extensive potential as pharmaceutical products treating different diseases^20^. *In vitro*, *in vivo*, and clinical trial studies have demonstrated the therapeutic role of terpenoids against various kinds of cancers^21^. Ursolic acid (UA, **1**) is an ursane-type pentacyclic triterpenoid widely distributed in many plants. As a bioactive constituent in many traditional Chinese medicines, UA and its derivatives possess a variety of biological activities including antimicrobial, anticancer, antiviral, antioxidant, antiulcer, antidiabetic, antiarrhythmic, anti-hypercholesterolemic, anti-hyperlipidemic, and anti-neurodegenerative activities^22–26^. To improve the therapeutic efficacy and bioavailability of UA, a large number of UA derivatives have been synthesised based on different strategies.

The introduction of nitrogen-containing heterocycle is usually a useful tactic in the structural modification of natural products because nitrogen atom can carry a positive charge and act as a hydrogen bond acceptor or donor that strongly influence the interaction between the molecule and its target^27^. Quinoline has been widely investigated as an important heterocyclic motif in the development of anticancer agents. The anticancer mechanisms of quinoline derivatives include inhibition of tyrosine kinases, proteasome, topoisomerase, tubulin polymerisation, and DNA repair^28^. 1,3,4-Oxadiazole is also regarded as an important scaffold for drug discovery owning to its metabolic profile and the ability to form hydrogen bonds with receptor sites^29^. Recent studies have pointed out that some 1,3,4-oxadiazole derivatives showed potent anticancer activity through inhibiting various targets, such as telomerase, histone deacetylase (HDAC), glycogen synthase kinase-3 (GSK), epidermal growth factor (EGF), and vascular endothelial growth factor (VEGF)^29^^,^^30^. In addition, 1,3,4-thiadiazole also represents a key heterocyclic motif in medicinal chemistry owing to its high electron-donating ability to form hydrogen bonds or to coordinate metal ions^31^. Some 1,3,4-thiadiazole derivatives have been reported for their considerable anticancer properties^32^^,^^33^. Molecular hybridisation may be a strategy to enhance activity or selectivity and overcome the side effects associated with the original compound^32^. Therefore, the introduction of quinoline, oxadiazole, and/or thiadiazole moieties to the molecule of UA may probably afford novel derivatives with promising anticancer activities. In continuation of our research on the novel UA derivatives with anticancer properties^34^, a series of novel UA derivatives bearing quinoline, oxadiazole, and thiadiazole moieties were designed and synthesised, and their anticancer activities and possible mechanisms of action were also investigated and presented.

## 2. Experimental

### 2.1. Chemistry

Melting points were measured on an XT-4 apparatus (Taike Corp., Beijing, China) and were uncorrected. IR spectra were measured on a Nexus 870 FT-IR spectrometer (Thermo Fisher Scientific, Waltham, MA, USA), and the absorption bands were expressed in cm^−1^. The HRMS spectra were recorded on a high-resolution mass spectrometer equipped with electrospray (ESI) and nanospray sources, and a quadrupole time of flight hybrid analyser (Q-TOF Premier/nanoAquity, Waters, Milford, MA, USA). ^1^H and ^13 ^C NMR spectra were obtained in CDCl_3_ on Bruker AV-300, AV-500 (Billerica, MA, USA) and DRX-600 NMR spectrometers using TMS as internal standard. Reactions and the resulted products were monitored by TLC which was carried out on TLC Silica gel 60 F_254_ Aluminium sheets from Merck KGaA, Darmstadt, Germany and visualised in UV light (254 nm). Silica gel (300 ∼ 400 mesh) for column chromatography was purchased from Qingdao Marine Chemical Factory, China. The reagents (chemicals), all being of A.R. grade, were purchased from Shanghai Chemical Reagent Company (Shanghai, China) and Energy Chemical (Shanghai, China). UA (95%) was bought from Jingzhu Biological Technology Co., Ltd. (Nanjing, China).

### 2.2. The preparation of compound 2

To a solution of UA (**1**, 2.0 g, 4.4 mmol) in acetone (180 mL) was added dropwise Jones reagent (1.9 mL), and the solution was stirred at room temperature for 5 h. Then 80 mL of isopropanol was added and the mixture was stirred for 30 min. At the end of reaction, the mixture was filtered, and the filtrate was concentrated *in vacuo*. The residue was recrystallised in methanol to afford pure compound **2** as a white solid (1.18 g, yield 80%). The spectral data of compound **2** were in accordance with the records of previous literature^35^.

### 2.3. General procedures for the synthesis of compounds 3a–d

Compounds **3a–d** was synthesised according to the previous literature^34^. Briefly, to a solution of differently substituted *o*-nitrobenzaldehyde (3 mmol) in 20 mL of EtOH/AcOH/H_2_O (2:2:1) was added reduced iron powder (1.68 g, 30 mmol) and concentrated hydrochloric acid (600 μL). The mixture was refluxed for 1 h. After cooling, the mixture was filtered to remove iron powder and the filtrate was extracted with CH_2_Cl_2_ (3 × 40 mL). The organic layer was combined, washed with water, saturated NaHCO_3_ solution and brine, dried over anhydrous Na_2_SO_4_, and concentrated *in vacuo* to give a crude product of differently substituted *o*-aminobenzaldehyde which could be used directly for the next step.

To a solution of compound **2** (0.67 g, 1.5 mmol) in EtOH was added the corresponding *o*-aminobenzaldehyde (3.0 mmol) and the saturated solution of KOH in EtOH. The mixture was refluxed under nitrogen atmosphere for 24 h and monitored by TLC. After cooling, the mixture was poured into 100 mL of ice-cold water and extracted with CH_2_Cl_2_ (3 × 40 mL). The organic layer was combined, washed with water and brine, dried over anhydrous Na_2_SO_4_, and concentrated *in vacuo* to give crude product, which was purified by silica gel column chromatography (petroleum ether-acetone 200:1, v/v) to afford compounds **3a–d**. Their spectral data were in accordance with the previous literature^34^.

### 2.4. General procedures for the synthesis of compounds 4a–h

To the solution of compounds **3a–d** (0.15 mmol) in benzene (5 mL) was added dropwise 200 μL of SOCl_2_ (1.5 mmol). The reaction mixture was refluxed at 80 °C for 3 h. After cooling, the solvent and excess SOCl_2_ were removed by concentration *in vacuo*. The corresponding products were dissolved in ether (8 mL) and the mixture of acylhydrazine (0.225 mmol), Et_3_N (45 μL), and CH_2_Cl_2_ (2 mL) were slowly added. The reaction mixture was stirred at room temperature for 8 ∼ 12 h, and monitored by TLC. At the end of reaction, the mixture was poured into 30 mL of ice-cold water and extracted with CH_2_Cl_2_ (3 × 40 mL). The organic layer was combined, washed with water, saturated NaHCO_3_ and brine, dried over anhydrous Na_2_SO_4_, and concentrated *in vacuo* to obtain a crude product, which was purified by silica gel column chromatography (petroleum ether-acetone 200:1 ∼ 10:1, v/v) to afford compounds **4a–h**.

#### 2.4.1. N-[ursa-12-en-(2,3)-quinoline-28-oyl]-acetohydrazide (4a)

Yellow powder; Yield 84%; M.p. 267 ∼ 269 °C; ^1^H NMR (500 MHz, CDCl_3_): *δ* 0.84 (s, 3H), 0.90 (s, 3H), 0.94 (d, *J* = 6.4 Hz, 3H), 0.98 (d, *J* = 5.0 Hz, 3H), 1.19 (s, 3H), 1.28 ∼ 1.35 (m, 2H), 1.42 (s, 3H), 1.44 (s, 3H), 1.50 ∼ 1.85 (m, 12H), 1.93 (m, 1H), 2.04 (s, 3H), 2.07 (m, 2H), 2.18 (m, 2H), 2.59 (d, *J* = 15.4 Hz, 1H), 2.97 (d, *J* = 15.4 Hz, 1H), 5.60 (s, 1H), 7.41 (t, *J* = 7.5 Hz, 1H), 7.58 (t, *J* = 7.5 Hz, 1H), 7.69 (d, *J* = 8.5 Hz, 1H), 7.70 (s, 1H), 8.00 (d, *J* = 8.5 Hz, 1H), 8.77 (d, *J* = 7.0 Hz, 1H, NH), 8.95 (d, *J* = 7.1 Hz, 1H, NH); ^13 ^C NMR (125 MHz, CDCl_3_): *δ* 15.48, 16.38, 17.30, 20.51, 20.80, 21.28, 23.48, 23.83, 25.08, 25.40, 27.99, 30.93, 32.41, 32.55, 36.28, 37.07, 39.12, 39.70, 39.84, 40.33, 42.78, 45.71, 46.39, 47.78, 53.21, 54.02, 125.60, 126.70, 127.10, 127.48, 128.17, 128.66, 129.09, 135.34, 139.36, 147.68, 166.00, 168.42, 173.07; IR (KBr, cm^−1^): 3251, 2953, 2924, 2854, 1618, 1492, 1458, 1378, 1081, 968, 759; HRMS (ESI): *m/z* [M + H]^+^ calcd. for C_39_H_54_N_3_O_2_: 596.4216; found: 596.4218.

#### 2.4.2. N-[5′-methoxy-ursa-12-en-(2,3)-quinoline-28-oyl]-acetohydrazide (4b)

Yellow powder; Yield 58%; M.p. 290 ∼ 292 °C; ^1^H NMR (500 MHz, CDCl_3_): *δ* 0.83 (s, 3H), 0.87 (s, 3H), 0.92 (d, *J* = 7.2 Hz, 3H), 0.97 (d, *J* = 7.5 Hz, 3H), 1.18 (s, 3H), 1.28 ∼ 1.35 (m, 2H), 1.39 (s, 3H), 1.42 (s, 3H), 1.47 ∼ 1.93 (m, 13H), 2.03 (s, 3H), 2.05 ∼ 2.18 (m, 4H), 2.57 (d, *J* = 15.1 Hz, 1H), 2.92 (d, *J* = 15.3 Hz, 1H), 3.91 (s, 3H, OCH_3_), 5.59 (s, 1H), 6.96 (d, *J* = 2.6 Hz, 1H), 7.25 (d, *J* = 7.6 Hz, 1H), 7.60 (s, 1H), 7.91 (d, *J* = 8.8 Hz, 1H), 8.78 (m, 1H, NH), 8.94 (m, 1H, NH); ^13 ^C NMR (125 MHz, CDCl_3_): *δ* 15.51, 16.43, 17.29, 20.51, 20.79, 21.28, 23.49, 23.73, 25.08, 25.43, 27.92, 30.94, 32.46, 32.56, 36.29, 37.08, 39.12, 39.72, 39.85, 40.47, 42.81, 45.68, 46.43, 47.72, 53.24, 53.97, 55.63 (OCH_3_), 104.21, 118.14, 126.10, 127.47, 127.81, 131.05, 134.31, 139.25, 144.34, 157.98, 163.27, 168.40 (C = O), 173.01 (C = O); IR (KBr, cm^−1^): 3249, 2953, 2925, 2855, 1621, 1492, 1457, 1378, 1222, 1080, 1030, 828, 668; HRMS (ESI): *m/z* [M + H]^+^ calcd. for C_40_H_56_N_3_O_3_: 626.4322; found: 626.4317.

#### 2.4.3. N-[5′-fluoro-ursa-12-en-(2,3)-quinoline-28-oyl]-acetohydrazide (4c)

Yellow powder; Yield 88%; M.p. 276 ∼ 278 °C; ^1^H NMR (500 MHz, CDCl_3_): *δ* 0.84 (s, 3H), 0.89 (s, 3H), 0.93 (d, *J* = 6.40 Hz, 3H), 0.98 (d, *J* = 6.2 Hz, 3H), 1.18 (s, 3H), 1.28 ∼ 1.35 (m, 2H), 1.40 (s, 3H), 1.43 (s, 3H), 1.45 ∼ 1.80 (m, 12H), 1.92 (m, 1H), 2.04 (s, 3H), 2.07 (m, 2H), 2.19 (m, 2H), 2.58 (d, *J* = 15.5 Hz, 1H), 2.95 (d, *J* = 15.5 Hz, 1H), 5.60 (s, 1H), 7.29 (dd, *J* = 9.0, 2.6 Hz, 1H), 7.35 (dt, *J* = 9.0, 2.8 Hz, 1H), 7.64 (s, 1H), 7.98 (dd, *J* = 9.1, 5.4 Hz, 1H), 8.89 (d, *J* = 7.0 Hz, 1H, NH), 8.96 (d, *J* = 7.0 Hz, 1H, NH); ^13 ^C NMR (125 MHz, CDCl_3_): *δ* 15.51, 16.42, 17.26, 20.51, 20.74, 21.25, 23.45, 23.75, 25.08, 25.42, 27.91, 30.90, 32.42, 32.54, 36.27, 37.09, 39.07, 39.67, 39.83, 40.33, 42.74, 45.66, 46.38, 47.70, 53.21, 54.00, 109.41 (d, *J* = 21.1 Hz), 118.40 (d, *J* = 28.4 Hz), 127.23, 127.44 (d, *J* = 9.9 Hz), 129.53, 131.43 (d, *J* = 9.1 Hz), 134.79, 139.19, 144.68, 160.15 (d, *J* = 244.7 Hz), 165.29 (d, *J* = 2.6 Hz), 165.62, 173.28; IR (KBr, cm^−1^): 3230, 2952, 2925, 2855, 1616, 1492, 1456, 1378, 1212, 1148, 1077, 969, 829, 802; HRMS (ESI): *m/z* [M + H]^+^ calcd. for C_39_H_53_FN_3_O_2_: 614.4122; found: 614.4128.

#### 2.4.4. N-[5′-chloro-ursa-12-en-(2,3)-quinoline-28-oyl]-acetohydrazide (4d)

White power; Yield 74%; M.p. 271 ∼ 273 °C; ^1^H NMR (300 MHz, CDCl_3_): *δ* 0.83 (s, 3H), 0.88 (s, 3H), 0.93 (d, *J* = 6.4 Hz, 3H), 0.98 (d, *J* = 6.3 Hz, 3H), 1.18 (s, 3H), 1.28 ∼ 1.35 (m, 2H), 1.40 (s, 3H), 1.42 (s, 3H), 1.50 ∼ 1.80 (m, 12H), 1.93 (d, *J* = 13.4 Hz, 1H), 2.03 (s, 3H), 2.06 (m, 2H), 2.18 (m, 2H), 2.58 (d, *J* = 15.7 Hz, 1H), 2.96 (d, *J* = 15.6 Hz, 1H), 5.60 (s, 1H), 7.51 (dd, *J* = 9.0, 2.1 Hz, 1H), 7.61 (s, 1H), 7.66 (d, *J* = 1.9 Hz, 1H), 7.92 (d, *J* = 9.0 Hz, 1H), 8.69 (brs, 1H, NH), 8.95 (brs, 1H, NH); ^13 ^C NMR (125 MHz, CDCl_3_): *δ* 15.53, 16.43, 17.27, 20.52, 20.79, 21.27, 23.46, 23.77, 25.09, 25.44, 27.93, 30.91, 32.44, 32.54, 36.28, 37.10, 39.09, 39.69, 39.84, 40.47, 42.77, 45.67, 46.41, 47.71, 53.23, 53.98, 125.29, 127.22, 127.61, 129.08, 129.70, 130.72, 131.14, 134.43, 139.27, 145.94, 165.41, 166.45, 173.27; IR (KBr, cm^−1^): 3234, 2949, 2924, 2855, 1616, 1479, 1456, 1378, 1184, 1070, 968, 919, 828, 753; HRMS (ESI): *m/z* [M + H]^+^ calcd. for C_39_H_53_ClN_3_O_2_: 630.3826; found: 630.3823.

#### 2.4.5. N-[ursa-12-en-(2,3)-quinoline-28-oyl]-pentanehydrazide (4e)

White powder; Yield 70%; M.p. 279 ∼ 281 °C; ^1^H NMR (500 MHz, CDCl_3_): *δ* 0.82 (s, 3H), 0.89 (s, 3H), 0.90 (t, *J* = 7.4 Hz, 3H), 0.94 (d, *J* = 6.4 Hz, 3H), 0.98 (d, *J* = 6.4 Hz, 3H), 1.19 (s, 3H), 1.26 ∼ 1.38 (m, 4H), 1.42 (s, 3H), 1.44 (s, 3H), 1.49 ∼ 1.80 (m, 14H), 1.94 (m, 1H), 2.07 (m, 2H), 2.19 (m, 2H), 2.24 (t, *J* = 7.5 Hz, 2H), 2.59 (d, *J* = 15.3 Hz, 1H), 2.96 (d, *J* = 15.4 Hz, 1H), 5.62 (s, 1H), 7.41 (t, *J* = 7.5 Hz, 1H), 7.58 (t, *J* = 7.6 Hz, 1H), 7.69 (d, *J* = 8.5 Hz, 1H), 7.70 (s, 1H), 7.99 (d, *J* = 8.5 Hz, 1H), 8.71 (d, *J* = 7.1 Hz, 1H, NH), 9.06 (d, *J* = 7.4 Hz, 1H, NH); ^13 ^C NMR (125 MHz, CDCl_3_): *δ* 13.86, 15.42, 16.35, 17.29, 20.55, 21.27, 22.37, 23.51, 23.80, 25.09, 25.47, 27.67, 27.95, 30.90, 32.42, 32.60, 33.92, 36.31, 37.04, 39.12, 39.70, 39.83, 40.42, 42.75, 45.71, 46.45, 47.65, 53.24, 54.08, 125.56, 126.69, 127.07, 127.41, 128.18, 128.63, 129.08, 135.39, 139.25, 147.60, 166.00, 168.38 (C = O), 173.00 (C = O); IR (KBr, cm^−1^): 3223, 2954, 2924, 2855, 1615, 1492, 1457, 1378, 1190, 1075, 969, 753; HRMS (ESI): *m/z* [M + H]^+^ calcd. for C_42_H_60_N_3_O_2_: 638.4686; found: 638.4690.

#### 2.4.6. N-[5′-methoxy-ursa-12-en-(2,3)-quinoline-28-oyl]-pentanehydrazide (4f)

White powder; Yield 46%; M.p. 283 ∼ 285 °C; ^1^H NMR (500 MHz, CDCl_3_): *δ* 0.81 (s, 3H), 0.89 (s, 3H), 0.90 (t, *J* = 7.4 Hz, 3H), 0.94 (d, *J* = 6.3 Hz, 3H), 0.98 (d, *J* = 6.4 Hz, 3H), 1.18 (s, 3H), 1.30 ∼ 1.38 (m, 4H), 1.39 (s, 3H), 1.42 (s, 3H), 1.47 ∼ 1.80 (m, 14H), 1.93 (m, 1H), 2.07 (m, 2H), 2.18 (m, 2H), 2.24 (t, *J* = 7.3 Hz, 2H), 2.57 (d, *J* = 15.3 Hz, 1H), 2.92 (d, *J* = 15.8 Hz, 1H), 3.90 (s, 3H, OCH_3_), 5.62 (s, 1H), 6.96 (s, 1H), 7.24 (d, *J* = 8.9 Hz, 1H), 7.59 (s, 1H), 7.89 (d, *J* = 9.0 Hz, 1H), 8.72 (d, *J* = 7.8 Hz, 1H, NH), 9.07 (d, *J* = 7.4 Hz, 1H, NH); ^13 ^C NMR (125 MHz, CDCl_3_): *δ* 13.85, 15.42, 16.33, 17.28, 20.49, 21.25, 22.36, 23.50, 23.79, 25.08, 25.35, 27.67, 27.93, 30.88, 32.41, 32.51, 33.89, 36.31, 37.03, 39.12, 39.69, 39.82, 40.09, 42.74, 45.72, 46.43, 47.65, 53.25, 54.08, 55.63 (OCH_3_), 104.20, 118.23, 126.12, 127.42, 127.84, 131.02, 134.37, 139.24, 144.34, 157.94, 163.26, 168.40 (C = O), 173.00 (C = O); IR (KBr, cm^−1^): 3226, 2954, 2924, 2854, 1618, 1492, 1459, 1379, 1222, 1080, 1032, 964, 829, 805; HRMS (ESI): *m/z* [M + H]^+^ calcd. for C_43_H_62_N_3_O_3_: 668.4791; found: 668.4799.

#### 2.4.7. N-[5′-fluoro-ursa-12-en-(2,3)-quinoline-28-oyl]-pentanehydrazide (4g)

White powder; Yield 77%; M.p. 289 ∼ 291 °C; ^1^H NMR (500 MHz, CDCl_3_): *δ* 0.82 (s, 3H), 0.88 (s, 3H), 0.90 (t, *J* = 7.4 Hz, 3H), 0.94 (d, *J* = 6.4 Hz, 3H), 0.97 (d, *J* = 6.5 Hz, 3H), 1.18 (s, 3H), 1.26 ∼ 1.38 (m, 4H), 1.40 (s, 3H), 1.43 (s, 3H), 1.45 ∼ 1.65 (m, 10H), 1.70 (m, 2H), 1.77 (m, 2H), 1.93 (d, *J* = 14.0 Hz, 1H), 2.07 (d, *J* = 11.6 Hz, 2H), 2.18 (dd, *J* = 8.7, 3.0 Hz, 2H), 2.24 (t, *J* = 7.5 Hz, 2H), 2.58 (d, *J* = 15.5 Hz, 1H), 2.95 (d, *J* = 15.5 Hz, 1H), 5.62 (s, 1H), 7.29 (dd, *J* = 9.1, 2.3 Hz, 1H), 7.35 (dt, *J* = 8.8, 2.3 Hz, 1H), 7.65 (s, 1H), 7.98 (dd, *J* = 9.1, 5.3 Hz, 1H), 8.74 (d, *J* = 7.3 Hz, 1H, NH), 9.06 (d, *J* = 7.4 Hz, 1H, NH); ^13 ^C NMR (125 MHz, CDCl_3_): *δ* 13.87, 15.42, 16.32, 17.26, 20.49, 21.25, 22.37, 23.49, 23.76, 25.05, 25.42, 27.70, 27.91, 30.88, 32.37, 32.52, 33.80, 36.26, 37.04, 39.09, 39.67, 39.81, 40.31, 42.70, 45.68, 46.35, 47.62, 53.22, 53.99, 109.41 (d, *J* = 20.7 Hz), 118.38 (d, *J* = 25.6 Hz), 127.33, 127.43 (d, *J* = 9.7 Hz), 129.53, 131.41 (d, *J* = 8.3 Hz), 134.80, 139.15, 144.66, 160.14 (d, *J* = 244.7 Hz), 165.28 (d, *J* = 2.1 Hz), 168.59, 172.89; IR (KBr, cm^−1^): 3238, 2954, 2925, 2870, 1614, 1493, 1456, 1378, 1286, 1213, 1148, 1077, 969, 829, 801; HRMS (ESI): *m/z* [M + H]^+^ calcd. for C_42_H_59_FN_3_O_2_: 656.4591; found: 656.4587.

#### 2.4.8. N-[5′-chloro-ursa-12-en-(2,3)-quinoline-28-oyl]-pentanehydrazide (4h)

Yellow power; Yield 70%; M.p. 305 ∼ 307 °C; ^1^H NMR (500 MHz, CDCl_3_): *δ* 0.81 (s, 3H), 0.88 (s, 3H), 0.90 (t, *J* = 7.4 Hz, 3H), 0.93 (d, *J* = 6.2 Hz, 3H), 0.98 (d, *J* = 6.2 Hz, 3H), 1.18 (s, 3H), 1.30 ∼ 1.38 (m, 4H), 1.40 (s, 3H), 1.43 (s, 3H), 1.49 ∼ 1.80 (m, 14H), 1.93 (d, *J* = 14.2 Hz, 1H), 2.07 (d, *J* = 11.2 Hz, 2H), 2.18 (d, *J* = 6.1 Hz, 2H), 2.23 (t, *J* = 7.4 Hz, 2H), 2.58 (d, *J* = 15.4 Hz, 1H), 2.96 (d, *J* = 15.4 Hz, 1H), 5.61 (s, 1H), 7.51 (d, *J* = 8.8 Hz, 1H), 7.61 (s, 1H), 7.67 (s, 1H), 7.92 (d, *J* = 8.2 Hz, 1H), 8.75 (d, *J* = 6.7 Hz, 1H, NH), 9.07 (d, *J* = 7.0 Hz, 1H, NH); ^13 ^C NMR (125 MHz, CDCl_3_): *δ* 13.90, 15.34, 16.47, 17.28, 20.52, 21.28, 22.38, 23.66, 23.85, 25.09, 25.43, 27.55, 27.98, 30.79, 32.42, 32.54, 33.93, 36.29, 37.03, 39.23, 39.70, 39.85, 40.34, 42.78, 45.58, 46.38, 47.67, 53.96, 54.09, 125.29, 127.39, 127.81, 129.12, 129.68, 130.72, 131.15, 134.50, 139.31, 145.91, 165.45, 166.46, 173.26; IR (KBr, cm^−1^): 3230, 2953, 2925, 2869, 1614, 1479, 1457, 1379, 1183, 1069, 965, 918, 828; HRMS (ESI): *m/z* [M + H]^+^ calcd. for C_42_H_59_ClN_3_O_2_: 672.4296; found: 672.4292.

### 2.5. General procedures for the synthesis of compounds 5a–h

To a solution of the corresponding intermediate **4a–h** (0.05 mmol) in toluene (5 mL) was added TsOH (10 mg, 0.05 mmol), and the mixture was refluxed at 110 °C for 6 h under the monitoring of TLC. Then the reaction mixture was diluted with toluene, washed with saturated NaHCO_3_ and brine, dried over anhydrous Na_2_SO_4_ and concentrated *in vacuo*. The residue was purified by silica gel column chromatography (petroleum ether-acetone 150:1 ∼ 80:1, v/v) to give compounds **5a–h**.

#### 2.5.1. N-2-[ursa-12-en-(2,3)-quinoline-28-oyl]-5-methyl-1,3,4-oxadiazole (5a)

Yellow solid; Yield 68%; M.p. 324 ∼ 326 °C; ^1^H NMR (300 MHz, CDCl_3_): *δ* 0.51 (s, 3H), 0.84 (s, 3H), 0.96 (d, *J* = 6.2 Hz, 3H), 1.00 (d, *J* = 6.2 Hz, 3H), 1.11 (m, 1H), 1.18 (s, 3H), 1.40 (s, 3H), 1.43 (s, 3H), 1.50 ∼ 1.80 (m, 12H), 1.91 (m, 2H), 2.05 (m, 2H), 2.32 (m, 1H), 2.47 (m, 1H), 2.48 (s, 3H), 2.56 (d, *J* = 15.8 Hz, 1H), 2.94 (d, *J* = 15.3 Hz, 1H), 5.42 (s, 1H), 7.40 (t, *J* = 7.2 Hz, 1H), 7.58 (t, *J* = 7.5 Hz, 1H), 7.67 (d, *J* = 6.0 Hz, 1H), 7.68 (s, 1H), 7.99 (d, *J* = 8.4 Hz, 1H); ^13 ^C NMR (125 MHz, CDCl_3_): *δ* 15.32, 16.48, 17.29, 20.55, 21.28, 22.84, 23.68, 23.86, 24.88, 25.45, 27.56, 30.80, 32.08, 32.58, 36.31, 38.94, 39.02, 39.23, 39.70, 40.41, 41.62, 42.23, 45.61, 46.45, 53.98, 54.16, 125.52, 126.66, 127.06, 127.42, 128.15, 128.77, 129.08, 135.33, 137.89, 147.59, 162.79, 166.15, 172.56; IR (KBr, cm^−1^): 2954, 2924, 2853, 1735, 1661, 1591, 1485, 1466, 1379, 1184, 1082, 966, 851, 748; HRMS (ESI): *m/z* [M + H]^+^ calcd. for C_39_H_52_N_3_O: 578.4110; found 578.4113.

#### 2.5.2. N-[5′-methoxy-ursa-12-en-(2,3)-quinoline-28-oyl]-5-methyl-1,3,4-oxadiazole (5b)

Yellow solid; Yield 73%; M.p. 313 ∼ 315 °C; ^1^H NMR (500 MHz, CDCl_3_): *δ* 0.50 (s, 3H), 0.83 (s, 3H), 0.96 (d, *J* = 6.4 Hz, 3H), 1.00 (d, *J* = 6.4 Hz, 3H), 1.14 (m, 1H), 1.17 (s, 3H), 1.36 (s, 3H), 1.40 (s, 3H), 1.42 ∼ 1.80 (m, 12H), 1.91 (m, 2H), 2.02 (m, 2H), 2.32 (m, 1H), 2.47 (m, 1H), 2.48 (s, 3H), 2.54 (d, *J* = 15.1 Hz, 1H), 2.90 (d, *J* = 15.5 Hz, 1H), 3.90 (s, 3H, OCH_3_), 5.42 (s, 1H), 6.94 (d, *J* = 2.6 Hz, 1H), 7.24 (dd, *J* = 9.2, 2.7 Hz, 1H), 7.57 (s, 1H), 7.88 (d, *J* = 9.2 Hz, 1H); ^13 ^C NMR (125 MHz, CDCl_3_): *δ* 15.32, 16.51, 17.28, 20.49, 21.27, 21.73, 22.83, 23.72, 23.88, 25.02, 25.39, 27.62, 30.84, 32.07, 32.53, 36.27, 39.05, 39.24, 39.69, 40.32, 41.87, 42.27, 45.60, 46.38, 54.08, 54.19, 55.61 (OCH_3_), 104.20, 119.31, 126.66, 127.83, 129.00, 130.56, 134.30, 137.87, 143.79, 157.23, 162.75, 163.44, 172.56; IR (KBr, cm^−1^): 2924, 2854, 1735, 1671, 1624, 1592, 1554, 1492, 1457, 1379, 1221, 1080, 1030, 964, 805, 734; HRMS (ESI): *m/z* [M + H]^+^ calcd. for C_40_H_54_N_3_O_2_: 608.4216; found 608.4219.

#### 2.5.3. N-[5′-fluoro-ursa-12-en-(2,3)-quinoline-28-oyl]-5-methyl-1,3,4-oxadiazole (5c)

Yellow solid; Yield 70%; M.p. 318 ∼ 320 °C; ^1^H NMR (500 MHz, CDCl_3_): *δ* 0.51 (s, 3H), 0.83 (s, 3H), 0.96 (d, *J* = 6.4 Hz, 3H), 1.01 (d, *J* = 6.4 Hz, 3H), 1.11 (m, 1H), 1.18 (s, 3H), 1.38 (s, 3H), 1.41 (s, 3H), 1.45 ∼ 1.78 (m, 12H), 1.87 (dd, *J* = 13.6, 4.8 Hz, 1H), 1.92 (d, *J* = 10.6 Hz, 1H), 2.04 (dd, *J* = 11.6, 2.7 Hz, 1H), 2.10 (dt, *J* = 18.8, 5.2 Hz, 1H), 2.32 (dt, *J* = 13.4, 4.6 Hz, 1H), 2.48 (s, 3H), 2.49 (m, 1H), 2.55 (d, *J* = 15.5 Hz, 1H), 2.92 (d, *J* = 15.6 Hz, 1H), 5.42 (s, 1H), 7.28 (m, 1H), 7.34 (dt, *J* = 8.8, 2.5 Hz, 1H), 7.62 (s, 1H), 7.97 (dd, *J* = 9.0, 5.3 Hz, 1H); ^13 ^C NMR (125 MHz, CDCl_3_): *δ* 15.34, 16.47, 17.28, 20.52, 21.28, 22.83, 23.66, 23.85, 24.87, 25.43, 27.55, 30.79, 32.11, 32.54, 36.29, 38.93, 39.01, 39.22, 39.70, 40.33, 41.61, 42.22, 45.58, 46.38, 53.97, 54.09, 109.38 (d, *J* = 19.5 Hz), 118.17 (d, *J* = 17.1 Hz), 126.56, 127.43 (d, *J* = 10.1 Hz), 129.68, 131.44 (d, *J* = 8.2 Hz), 134.72, 137.90, 144.69, 160.15 (d, *J* = 244.8 Hz), 162.79, 165.45 (d, *J* = 3.1 Hz), 172.54; IR (KBr, cm^−1^): 2952, 2924, 2854, 1689, 1628, 1592, 1556, 1492, 1456, 1378, 1212, 1148, 1077, 968, 829; HRMS (ESI): *m/z* [M + H]^+^ calcd. for C_39_H_51_FN_3_O: 596.4016; found 596.4023.

#### 2.5.4. N-[5′-chloro-ursa-12-en-(2,3)-quinoline-28-oyl]-5-methyl-1,3,4-oxadiazole (5d)

Yellow solid; Yield 73%; M.p. 313 ∼ 315 °C; ^1^H NMR (500 MHz, CDCl_3_): *δ* 0.51 (s, 3H), 0.83 (s, 3H), 0.96 (d, *J* = 6.4 Hz, 3H), 1.00 (d, *J* = 6.4 Hz, 3H), 1.11 (m, 1H), 1.18 (s, 3H), 1.38 (s, 3H), 1.41 (s, 3H), 1.42 ∼ 1.80 (m, 12H), 1.87 (dd, *J* = 13.6, 4.8 Hz, 1H), 1.92 (m, 1H), 2.04 (dd, *J* = 11.4, 2.6 Hz, 1H), 2.10 (dt, *J* = 18.2, 5.2 Hz, 1H), 2.32 (m, 1H), 2.48 (s, 3H), 2.49 (m, 1H), 2.55 (d, *J* = 15.5 Hz, 1H), 2.93 (d, *J* = 15.5 Hz, 1H), 5.42 (s, 1H), 7.50 (dd, *J* = 8.9, 2.0 Hz, 1H), 7.59 (s, 1H), 7.65 (d, *J* = 1.9 Hz, 1H), 7.91 (d, *J* = 9.0 Hz, 1H); ^13 ^C NMR (125 MHz, CDCl_3_): *δ* 15.32, 16.51, 17.29, 20.49, 21.27, 21.73, 22.83, 23.72, 23.88, 25.02, 25.39, 27.62, 30.84, 32.07, 32.53, 36.27, 39.05, 39.24, 39.69, 40.32, 41.87, 42.27, 45.60, 46.38, 54.08, 54.19, 125.26, 127.38, 127.69, 129.00, 129.79, 130.63, 131.18, 134.69, 139.20, 145.98, 162.85, 165.47, 172.56; IR (KBr, cm^−1^): 2924, 2854, 1735, 1671, 1624, 1592, 1554, 1492, 1457, 1379, 1221, 1080, 1030, 964, 805, 734; HRMS (ESI): *m/z* [M + H]^+^ calcd. for C_39_H_51_ClN_3_O: 612.3721; found 612.3726.

#### 2.5.5. N-2-[ursa-12-en-(2,3)-quinoline-28-oyl]-5-butyl-1,3,4-oxadiazole (5e)

White solid; Yield 78%; M.p. 328 ∼ 330 °C; ^1^H NMR (500 MHz, CDCl_3_): *δ* 0.49 (s, 3H), 0.83 (s, 3H), 0.94 (t, *J* = 7.8 Hz, 3H), 0.96 (d, *J* = 6.9 Hz, 3H), 1.01 (d, *J* = 6.4 Hz, 3H), 1.11 (m, 1H), 1.18 (s, 3H), 1.38 (m, 2H), 1.40 (s, 3H), 1.43 (s, 3H), 1.45 ∼ 1.73 (m, 14H), 1.95 (m, 2H), 2.01 (dd, *J* = 11.2, 2.2 Hz, 1H), 2.11 (dt, *J* = 18.2, 5.2 Hz, 1H), 2.32 (m, 1H), 2.47 (d, *J* = 11.2 Hz, 1H), 2.56 (d, *J* = 15.4 Hz, 1H), 2.81 (m, 2H), 2.93 (d, *J* = 15.4 Hz, 1H), 5.41 (s, 1H), 7.40 (t, *J* = 7.5 Hz, 1H), 7.57 (t, *J* = 7.5 Hz, 1H), 7.66 (d, *J* = 6.8 Hz, 1H), 7.67 (s, 1H), 7.99 (d, *J* = 8.5 Hz, 1H); ^13 ^C NMR (125 MHz, CDCl_3_): *δ* 13.85, 15.50, 16.43, 17.30, 20.52, 21.27, 22.83, 23.47, 23.84, 25.15, 25.44, 28.01, 30.37, 30.75, 31.59, 32.07, 32.41, 36.31, 38.95, 39.12, 39.71, 39.85, 40.42, 41.74, 42.83, 45.70, 47.94, 53.26, 54.08, 125.57, 126.69, 127.07, 127.48, 128.17, 128.60, 128.99, 135.07, 137.87, 147.24, 162.81, 167.82, 172.51; IR (KBr, cm^−1^): 2954, 2925, 2855, 1739, 1583, 1552, 1492, 1456, 1378, 1189, 1081, 967, 753, 723; HRMS (ESI): *m/z* [M + H]^+^ calcd. for C_42_H_58_N_3_O: 620.4580; found: 620.4572.

#### 2.5.6. N-[5′-methoxy-ursa-12-en-(2,3)-quinoline-28-oyl]-5-butyl-1,3,4-oxadiazole (5f)

White solid; Yield 63%; M.p. 304 ∼ 306 °C; ^1^H NMR (500 MHz, CDCl_3_): *δ* 0.49 (s, 3H), 0.83 (s, 3H), 0.94 (t, *J* = 7.2 Hz, 3H), 0.96 (d, *J* = 7.0 Hz, 3H), 1.01 (d, *J* = 6.4 Hz, 3H), 1.14 (m, 1H), 1.18 (s, 3H), 1.27 (m, 2H), 1.37 (s, 3H), 1.41 (s, 3H), 1.46 ∼ 1.80 (m, 14H), 1.94 (m, 2H), 2.01 (d, *J* = 11.3 Hz, 1H), 2.10 (m, 1H), 2.33 (m, 1H), 2.47 (d, *J* = 12.0 Hz, 1H), 2.54 (d, *J* = 15.6 Hz, 1H), 2.81 (m, 2H), 2.90 (d, *J* = 15.8 Hz, 1H), 3.90 (s, 3H, OCH_3_), 5.41 (s, 1H), 6.94 (s, 1H), 7.23 (d, *J* = 8.7 Hz, 1H), 7.56 (s, 1H), 7.88 (d, *J* = 8.3 Hz, 1H); ^13 ^C NMR (125 MHz, CDCl_3_): *δ* 13.88, 15.52, 16.42, 17.29, 20.50, 21.26, 22.78, 23.46, 23.82, 25.13, 25.43, 28.00, 29.47, 30.89, 31.72, 32.39, 32.56, 36.29, 37.01, 39.10, 39.70, 39.85, 40.34, 42.81, 45.68, 46.39, 47.93, 53.24, 54.01, 55.72 (OCH_3_), 104.37, 119.27, 126.75, 127.75, 128.90, 130.56, 134.24, 137.89, 143.88, 157.36, 163.37, 165.27, 172.52; IR (KBr, cm^−1^): 2955, 2925, 2855, 1671, 1625, 1492, 1457, 1378, 1221, 1080, 1029, 972, 829; HRMS (ESI): *m/z* [M + H]^+^ calcd. for C_43_H_60_N_3_O_2_: 650.4686; found: 650.4692.

#### 2.5.7. N-[5′-fluoro-ursa-12-en-(2,3)-quinoline-28-oyl]-5-butyl-1,3,4-oxadiazole (5g)

White solid; Yield 53%; M.p. 294 ∼ 296 °C; ^1^H NMR (500 MHz, CDCl_3_): *δ* 0.49 (s, 3H), 0.83 (s, 3H), 0.94 (t, *J* = 7.4 Hz, 3H), 0.96 (d, *J* = 6.4 Hz, 3H), 1.01 (d, *J* = 6.4 Hz, 3H), 1.12 (m, 1H), 1.18 (s, 3H), 1.38 (s, 3H), 1.40 (m, 2H), 1.41 (s, 3H), 1.45–1.80 (m, 14H), 1.95 (m, 2H), 2.01 (dd, *J* = 11.6, 2.1 Hz, 1H), 2.10 (dt, *J* = 18.2, 5.2 Hz, 1H), 2.32 (m, 1H), 2.47 (d, *J* = 11.2 Hz, 1H), 2.55 (d, *J* = 15.5 Hz, 1H), 2.81 (m, 2H), 2.92 (d, *J* = 15.6 Hz, 1H), 5.41 (s, 1H), 7.27 (m, 1H), 7.34 (dt, *J* = 8.7, 2.1 Hz, 1H), 7.62 (s, 1H), 7.97 (dd, *J* = 9.0, 5.3 Hz, 1H); ^13 ^C NMR (125 MHz, CDCl_3_): *δ* 13.79, 15.30, 16.62, 17.24, 20.51, 21.24, 22.12, 23.64, 23.84, 25.15, 25.41, 27.49, 29.09, 30.79, 31.59, 32.52, 32.59, 36.27, 38.88, 39.03, 39.17, 39.69, 40.32, 41.65, 42.25, 45.55, 46.36, 54.09, 54.12, 109.39 (d, *J* = 21.1 Hz), 118.37 (d, *J* = 24.5 Hz), 126.47, 127.43 (d, *J* = 9.8 Hz), 129.67, 131.42 (d, *J* = 7.4 Hz), 134.70, 138.08, 144.64, 160.15 (d, *J* = 244.5 Hz), 165.44 (d, *J* = 1.3 Hz), 166.25, 172.21; IR (KBr, cm^−1^): 2955, 2925, 2855, 1730, 1627, 1553, 1492, 1456, 1379, 1286, 1213, 1147, 1077, 968, 828, 803; HRMS (ESI): *m/z* [M + H]^+^ calcd. for C_42_H_57_FN_3_O: 638.4486; found: 638.4484.

#### 2.5.8. N-[5′-chloro-ursa-12-en-(2,3)-quinoline-28-oyl]-5-butyl-1,3,4-oxadiazole (5h)

Yellow solid; Yield 63%; M.p. 304 ∼ 306 °C; ^1^H NMR (500 MHz, CDCl_3_): *δ* 0.49 (s, 3H), 0.82 (s, 3H), 0.94 (t, *J* = 7.3 Hz, 3H), 0.96 (d, *J* = 5.9 Hz, 3H), 1.01 (d, *J* = 6.4 Hz, 3H), 1.12 (m, 1H), 1.18 (s, 3H), 1.38 (s, 3H), 1.40 (m, 2H), 1.41(s, 3H), 1.46 ∼ 1.80 (m, 14H), 1.95 (m, 2H), 2.01 (m, 1H), 2.10 (dt, *J* = 18.1, 5.5 Hz, 1H), 2.32 (m, 1H), 2.47 (d, *J* = 11.3 Hz, 1H), 2.55 (d, *J* = 16.1 Hz, 1H), 2.81 (m, 2H), 2.92 (d, *J* = 15.4 Hz, 1H), 5.41 (s, 1H), 7.50 (d, *J* = 8.4 Hz, 1H), 7.58 (s, 1H), 7.65 (s, 1H), 7.92 (d, *J* = 8.6 Hz, 1H); ^13 ^C NMR (125 MHz, CDCl_3_): *δ* 13.88, 15.52, 16.42, 17.29, 20.50, 21.26, 22.78, 23.46, 23.82, 25.13, 25.42, 28.00, 29.47, 30.89, 31.72, 32.39, 32.56, 36.29, 37.01, 39.10, 39.70, 39.84, 40.34, 42.81, 45.68, 46.38, 47.93, 53.24, 54.01, 125.26, 127.40, 127.61, 129.08, 129.79, 130.72, 131.25, 134.78, 139.40, 145.91, 165.45, 166.28, 172.17; IR (KBr, cm^−1^): 2955, 2925, 2855, 1671, 1625, 1492, 1457, 1378, 1221, 1080, 1029, 972, 829; HRMS (ESI): *m/z* [M + H]^+^ calcd. for C_42_H_57_ClN_3_O: 654.4190; found: 654.4185.

### 2.6. General procedures for the synthesis of compounds 6a–h

To the solution of corresponding intermediate **4a–h** (0.05 mmol) in toluene (5 mL) was added 35 mg of Lawesson reagent (0.05 mmol), and the mixture was refluxed at 110 °C for 6 h. At the end of reaction, the mixture was concentrated *in vacuo* and extracted with CH_2_Cl_2_ (50 mL) for three times. The organic layer was combined, washed with water, saturated NaHCO_3_ and brine, dried over anhydrous Na_2_SO_4_, and concentrated *in vacuo*. The residue was purified by silica gel column chromatography (petroleum ether-acetone 150:1 ∼ 80:1, v/v) to give compounds **6a–h**.

#### 2.6.1. N-2-[ursa-12-en-(2,3)-quinoline-28-oyl]-5-methyl-1,3,4-thiadiazole (6a)

Yellow resin; Yield: 44%; ^1^H NMR (500 MHz, CDCl_3_): *δ* 0.51 (s, 3H), 0.84 (s, 3H), 0.97 (d, *J* = 6.3 Hz, 3H), 1.01 (d, *J* = 6.4 Hz, 3H), 1.08 (m, 1H), 1.18 (s, 3H), 1.40 (s, 3H), 1.43 (s, 3H), 1.50 ∼ 1.80 (m, 12H), 1.91 (m, 2H), 2.05 (m, 2H), 2.32 (m, 1H), 2.48 (s, 3H), 2.49 (m, 1H), 2.56 (d, *J* = 15.6 Hz, 1H), 2.94 (d, *J* = 15.5 Hz, 1H), 5.42 (s, 1H), 7.40 (t, *J* = 7.8 Hz, 1H), 7.58 (t, *J* = 7.6 Hz, 1H), 7.67 (d, *J* = 7.9 Hz, 1H), 7.68 (s, 1H), 7.99 (d, *J* = 8.4 Hz, 1H); ^13 ^C NMR (125 MHz, CDCl_3_): *δ* 15.32, 16.48, 17.29, 20.56, 21.28, 22.84, 23.68, 23.86, 24.88, 25.45, 27.56, 30.81, 32.09, 32.58, 36.31, 38.95, 39.03, 39.24, 39.71, 40.42, 41.62, 42.22, 45.61, 46.45, 53.98, 54.17, 125.53, 126.66, 127.07, 127.26, 128.13, 128.75, 129.08, 135.34, 137.88, 147.58, 162.79, 166.14, 172.56; IR (KBr, cm^−1^): 2956, 2924, 2853, 2361,1732, 1592, 1495, 1459, 1378, 1076, 1021, 966, 752, 724; HRMS (ESI): *m/z* [M + H]^+^ calcd. for C_39_H_52_N_3_S: 594.3882; found: 594.3889.

#### 2.6.2. N-[5′-methoxy-ursa-12-en-(2,3)-quinoline-28-oyl]-5-methyl-1,3,4-thiadiazole (6b)

Yellow resin; Yield: 33%; ^1^H NMR (500 MHz, CDCl_3_): *δ* 0.52 (s, 3H), 0.82 (s, 3H), 1.00 (d, *J* = 6.4 Hz, 3H), 1.03 (d, *J* = 6.4 Hz, 3H), 1.11 (m, 1H), 1.18 (s, 3H), 1.40 (s, 3H), 1.44 (s, 3H), 1.50 ∼ 1.90 (m, 12H), 1.90 ∼ 2.20 (m, 4H), 2.38 (m, 1H), 2.46 (m, 1H), 2.48 (s, 3H), 2.53 (d, *J* = 15.5 Hz, 1H), 2.88 (d, *J* = 15.5 Hz, 1H), 3.89 (s, 3H, OCH_3_), 5.42 (s, 1H), 6.94 (d, *J* = 2.7 Hz, 1H), 7.23 (d, *J* = 9.2, 2.8 Hz, 1H), 7.55 (s, 1H), 7.87 (d, *J* = 9.2 Hz, 1H); ^13 ^C NMR (125 MHz, CDCl_3_): *δ* 15.32, 16.48, 17.28, 20.53, 21.29, 22.84, 23.68, 23.85, 24.88, 25.37, 27.56, 30.80, 32.08, 32.57, 36.31, 38.94, 39.02, 39.24, 39.71, 40.11, 41.63, 42.23, 45.62, 46.47, 53.99, 54.17, 55.62, 104.21, 119.30, 126.67, 127.79, 128.93, 130.54, 134.27, 137.88, 143.78, 157.25, 162.79, 163.44, 172.57; IR (KBr, cm^−1^): 2961, 2924, 2853, 1731, 1624, 1592, 1493, 1456, 1376, 1261, 1221, 1081, 1025, 964, 872, 705; HRMS (ESI): *m/z* [M + H]^+^ calcd. for C_40_H_54_N_3_OS: 624.3988; found: 624.3997.

#### 2.6.3. N-[5′-fluoro-ursa-12-en-(2,3)-quinoline-28-oyl]-5-methyl-1,3,4-thiadiazole (6c)

Yellow resin; Yield: 36%; ^1^H NMR (500 MHz, CDCl_3_): *δ* 0.51 (s, 3H), 0.83 (s, 3H), 0.99 (d, *J* = 6.4 Hz, 3H), 1.02 (d, *J* = 6.4 Hz, 3H), 1.11 (m, 1H), 1.18 (s, 3H), 1.40 (s, 3H), 1.45 (s, 3H), 1.50 ∼ 1.80 (m, 12H), 1.90 ∼ 2.10 (m, 4H), 2.35 (m, 1H), 2.48 (s, 3H), 2.49 (m, 1H), 2.54 (d, *J* = 15.6 Hz, 1H), 2.91 (d, *J* = 15.6 Hz, 1H), 5.42 (s, 1H), 7.28 (m, 1H), 7.33 (dt, *J* = 9.2, 2.4 Hz, 1H), 7.61 (s, 1H), 7.96 (dd, *J* = 9.0, 5.4 Hz, 1H); ^13 ^C NMR (125 MHz, CDCl_3_): *δ* 15.32, 16.52, 17.28, 20.48, 21.27, 22.82, 23.72, 23.86, 24.83, 25.39, 27.62, 30.83, 32.07, 32.53, 36.26, 38.94, 39.04, 39.24, 39.69, 40.31, 41.64, 42.27, 45.59, 46.37, 53.98, 54.18, 109.37 (d, *J* = 21.1 Hz), 118.30 (d, *J* = 26.7 Hz), 126.70, 127.41 (d, *J* = 9.8 Hz), 129.66, 131.45 (d, *J* = 10.5 Hz), 134.66, 137.98, 144.67, 160.12 (d, *J* = 244.6 Hz), 162.78, 165.44 (d, *J* = 2.8 Hz), 172.58; IR (KBr, cm^−1^): 2953, 2924, 2854, 1688, 1628, 1592, 1555, 1492, 1458, 1378, 1215, 1148, 1071, 962, 827; HRMS (ESI): *m/z* [M + H]^+^ calcd. for C_39_H_51_FN_3_S: 612.3788; found: 612.3786.

#### 2.6.4. N-[5′-chloro-ursa-12-en-(2,3)-quinoline-28-oyl]-5-methyl-1,3,4-thiadiazole (6d)

Yellow resin; Yield: 42%; ^1^H NMR (500 MHz, CDCl_3_): *δ* 0.51 (s, 3H), 0.84 (s, 3H), 1.00 (d, *J* = 6.4 Hz, 3H), 1.03 (d, *J* = 6.4 Hz, 3H), 1.12 (m, 1H), 1.18 (s, 3H), 1.40 (s, 3H), 1.44 (s, 3H), 1.50 ∼ 1.90 (m, 12H), 1.90 ∼ 2.20 (m, 4H), 2.35 (m, 1H), 2.48 (s, 3H), 2.49 (m, 1H), 2.54 (d, *J* = 15.6 Hz, 1H), 2.91 (d, *J* = 15.6 Hz, 1H), 5.42 (s, 1H), 7.50 (dd, *J* = 9.0, 2.2 Hz, 1H), 7.57 (s, 1H), 7.64 (d, *J* = 2.2 Hz, 1H), 7.91 (d, *J* = 8.4 Hz, 1H); ^13 ^C NMR (125 MHz, CDCl_3_): *δ* 15.32, 16.48, 17.30, 20.51, 21.28, 22.83, 23.68, 23.84, 24.88, 25.40, 27.56, 30.79, 32.07, 32.53, 36.28, 38.91, 39.02, 39.24, 39.71, 40.33, 41.86, 42.28, 45.61, 46.41, 53.99, 54.17, 125.29, 127.37, 127.60, 129.06, 129.73, 130.71, 131.12, 134.43, 139.41, 145.93, 162.69, 166.49, 172.55; IR (KBr, cm^−1^): 2927, 2852, 1738, 1669, 1620, 1582, 1567, 1472, 1463, 1380, 1222, 1087, 1030, 973, 872, 758; HRMS (ESI): *m/z* [M + H]^+^ calcd. for C_39_H_51_ClN_3_S: 628.3492; found: 628.3497.

#### 2.6.5. N-2-[ursa-12-en-(2,3)-quinoline-28-oyl]-5-butyl-1,3,4-thiadiazole (6e)

Yellow resin; Yield: 41%; ^1^H NMR (500 MHz, CDCl_3_): *δ* 0.51 (s, 3H), 0.82 (s, 3H), 0.94 (t, *J* = 7.4 Hz, 3H), 0.96 (d, *J* = 6.9 Hz, 3H), 1.01 (d, *J* = 6.4 Hz, 3H), 1.09 (m, 1H), 1.18 (s, 3H), 1.38 (m, 2H), 1.40 (s, 3H), 1.43 (s, 3H), 1.45 ∼ 1.80 (m, 14H), 1.90 ∼ 2.10 (m, 3H), 2.12 (m, 1H), 2.32 (m, 1H), 2.47 (d, *J* = 11.2 Hz, 1H), 2.56 (d, *J* = 15.5 Hz, 1H), 2.82 (m, 2H), 2.93 (d, *J* = 15.5 Hz, 1H), 5.42 (s, 1H), 7.40 (t, *J* = 7.5 Hz, 1H), 7.57 (t, *J* = 7.8 Hz, 1H), 7.66 (d, *J* = 6.9 Hz, 1H), 7.67 (s, 1H), 7.99 (d, *J* = 8.5 Hz, 1H); ^13 ^C NMR (125 MHz, CDCl_3_): *δ* 13.84, 15.51, 16.42, 17.42, 20.47, 21.31, 22.82, 23.49, 23.86, 25.16, 25.40, 28.02, 30.37, 30.74, 31.58, 32.07, 32.44, 36.33, 38.96, 39.17, 39.78, 39.90, 40.38, 41.74, 42.86, 45.62, 47.98, 53.97, 54.20, 125.58, 126.63, 127.03, 127.47, 128.18, 128.59, 128.98, 135.06, 137.87, 147.55, 162.79, 166.13, 172.56; IR (KBr, cm^−1^): 2956, 2925, 2854, 1736, 1583, 1452, 1456, 1379, 1110, 1074, 966, 857, 753, 727; HRMS (ESI): *m/z* [M + H]^+^ calcd. for C_42_H_58_N_3_S: 636.4351; found: 636.4358.

#### 2.6.6. N-2-[5′-methoxy-ursa-12-en-(2,3)-quinoline-28-oyl]-5-butyl-1,3,4-thiadiazole (6f)

Yellow resin; Yield: 38%; ^1^H NMR (500 MHz, CDCl_3_): *δ* 0.52 (s, 3H), 0.83 (s, 3H), 0.98 (t, *J* = 7.4 Hz, 3H), 1.00 (d, *J* = 6.4 Hz, 3H), 1.05 (d, *J* = 6.4 Hz, 3H), 1.12 (m, 1H), 1.19 (s, 3H), 1.37 (m, 2H), 1.40 (s, 3H), 1.45 (s, 3H), 1.45 ∼ 1.80 (m, 13H), 1.87 (d, *J* = 13.4 Hz, 1H), 1.90 ∼ 2.10 (m, 4H), 2.37 (m, 1H), 2.47 (d, *J* = 11.3 Hz, 1H), 2.53 (d, *J* = 15.5 Hz, 1H), 2.82 (m, 2H), 2.89 (d, *J* = 15.2 Hz, 1H), 3.90 (s, 3H, OCH_3_), 5.42 (s, 1H), 6.94 (s, 1H), 7.23 (d, *J* = 9.1 Hz, 1H), 7.56 (s, 1H), 7.87 (d, *J* = 9.0 Hz, 1H); ^13 ^C NMR (125 MHz, CDCl_3_): *δ* 13.88, 15.53, 16.48, 17.28, 20.53, 21.28, 22.84, 23.46, 23.85, 25.12, 25.37, 27.56, 30.39, 30.81, 31.60, 32.08, 32.56, 36.33, 39.03, 39.24, 39.71, 39.92, 40.38, 41.75, 42.86, 45.63, 47.93, 53.98, 54.18, 55.62 (OCH_3_), 104.21, 119.30, 126.67, 127.80, 128.93, 130.54, 134.27, 137.88, 143.78, 157.25, 162.79, 166.17, 172.57; IR (KBr, cm^−1^): 2957, 2922, 2856, 1673, 1617, 1472, 1450, 1383, 1212, 1085, 973, 875; HRMS (ESI): *m/z* [M + H]^+^ calcd. for C_43_H_60_N_3_OS: 666.4457; found: 666.4465.

#### 2.6.7. N-2-[5′-fluoro-ursa-12-en-(2,3)-quinoline-28-oyl]-5-butyl-1,3,4-thiadiazole (6g)

Yellow resin; Yield: 43%; ^1^H NMR (500 MHz, CDCl_3_): *δ* 0.52 (s, 3H), 0.83 (s, 3H), 0.94 (t, *J* = 7.4 Hz, 3H), 0.96 (d, *J* = 6.4 Hz, 3H), 1.01 (d, *J* = 6.4 Hz, 3H), 1.11 (m, 1H), 1.18 (s, 3H), 1.40 (s, 3H), 1.42 (m, 2H), 1.43 (s, 3H), 1.45 ∼ 1.80 (m, 14H), 1.90 ∼ 2.03 (m, 3H), 2.10 (m, 1H), 2.32 (m, 1H), 2.47 (d, *J* = 11.2 Hz, 1H), 2.55 (d, *J* = 15.4 Hz, 1H), 2.82 (m, 2H), 2.93 (d, *J* = 15.6 Hz, 1H), 5.42 (s, 1H), 7.27 (m, 1H), 7.34 (dt, *J* = 8.8, 2.1 Hz, 1H), 7.62 (s, 1H), 7.97 (dd, *J* = 9.1, 5.6 Hz, 1H); ^13 ^C NMR (125 MHz, CDCl_3_): *δ* 13.80, 15.49, 16.40, 17.42, 20.51, 21.28, 22.81, 23.48, 23.83, 25.18, 25.41, 28.00, 30.37, 30.73, 31.58, 32.06, 32.41, 36.28, 38.95, 39.11, 39.70, 39.90, 40.33, 41.75, 42.88, 45.71, 47.95, 53.97, 54.24, 109.42 (d, *J* = 21.4 Hz), 118.36 (d, *J* = 25.7 Hz), 126.48, 127.43 (d, *J* = 9.9 Hz), 129.68, 131.46 (d, *J* = 9.9 Hz), 134.74, 138.09, 144.70, 160.14 (d, *J* = 244.6 Hz), 165.33 (d, *J* = 2.4 Hz), 166.27, 172.27; IR (KBr, cm^−1^): 2957, 2926, 2853, 1733, 1630, 1558, 1487, 1452, 1381, 1282, 1218, 1139, 1081, 957, 853; HRMS (ESI): *m/z* [M + H]^+^ calcd. for C_42_H_57_FN_3_S: 654.4257; found: 654.4251.

#### 2.6.8. N-2-[5′-chloro-ursa-12-en-(2,3)-quinoline-28-oyl]-5-butyl-1,3,4-thiadiazole (6h)

Yellow resin; Yield: 49%; ^1^H NMR (500 MHz, CDCl_3_): *δ* 0.52 (s, 3H), 0.83 (s, 3H), 0.94 (t, *J* = 7.3 Hz, 3H), 0.96 (d, *J* = 6.0 Hz, 3H), 1.01 (d, *J* = 6.4 Hz, 3H), 1.11 (m, 1H), 1.18 (s, 3H), 1.40 (s, 3H), 1.42 (m, 2H), 1.43 (s, 3H), 1.45 ∼ 1.80 (m, 14H), 1.90 ∼ 2.02 (m, 3H), 2.10 (m, 1H), 2.31 (m, 1H), 2.47 (d, *J* = 11.3 Hz, 1H), 2.55 (d, *J* = 16.0 Hz, 1H), 2.82 (m, 2H), 2.93 (d, *J* = 15.4 Hz, 1H), 5.42 (s, 1H), 7.50 (d, *J* = 8.4 Hz, 1H), 7.58 (s, 1H), 7.65 (s, 1H), 7.92 (d, *J* = 8.7 Hz, 1H); ^13 ^C NMR (125 MHz, CDCl_3_): *δ* 13.84, 15.47, 16.39, 17.29, 20.50, 21.27, 22.81, 23.47, 23.83, 25.14, 25.39, 27.99, 30.33, 30.93, 31.58, 32.06, 32.40, 36.26, 39.03, 39.23, 39.69, 39.82, 40.35, 41.78, 42.86, 45.63, 47.93, 53.97, 54.18, 125.28, 127.30, 127.59, 129.03, 129.73, 130.69, 131.10, 134.41, 139.34, 145.91, 162.80, 166.48, 172.53; IR (KBr, cm^−1^): 2958, 2921, 2856, 1672, 1622, 1493, 1461, 1381, 1227, 1076, 1033, 973, 852; HRMS (ESI): *m/z* [M + H]^+^ calcd. for C_42_H_57_ClN_3_S: 670.3962; found: 670.3970.

### 2.7. Biological assay

#### 2.7.1. Cell lines and culture

Human breast cancer cell line (MDA-MB-231), cervical carcinoma cell line (HeLa), the liver cancer cell line (SMMC-7721) and normal hepatocyte cell line (QSG-7701) were maintained in Dulbecco Modified Eagle Medium (DMEM) containing 4.0 mM L-Glutamine and 4500 mg/L Glucose supplemented with 10% (v/v) foetal bovine serum (FBS) and 100 U/mL penicillin/streptomycin at 37 °C in humidified atmosphere of 5% CO_2_ and 95% air.

#### 2.7.2. MTT assay

Exponentially growing MDA-MB-231, Hela and SMMC-7721 cell lines were seeded into 96-well plate for 100 μL and treated with different concentrations of the synthetic compounds for 72 h, and then 10 μL of MTT (10 mg/mL) was added and incubation for 3 ∼ 4 h at 37 °C. The generated purple formazan crystals from viable can be dissolved by adding 100 μL DMSO. The plates were swirled gently for 5 min and quantified by measuring the OD of the plates at the wavelength of 540 nm. Each concentration was repeated in three wells and the same experimental condition was provided for all compounds. The results were expressed as IC_50_ values with standard deviations, which was defined as the concentration at which 50% survival of cells was discerned. Etoposide was co-assayed as positive control.

#### 2.7.3. Cell apoptosis analysis

The extent of apoptosis was quantitatively measured using Annexin V-FITC/PI dual staining assay^36^. HeLa cells were seeded into a six-well plate at 5 × 10^5^ cells per well in 10% foetal calf serum (FBS)-DMEM into six-well plates and treated with different concentrations of the indicated compound **4d** for 48 h. The cells were detached with 0.25% trypsin, washed with ice-cold PBS for twice and then resuspended in 1 × Binding buffer (0.1 M Hepes/NaOH (pH 7.4), 1.4 M NaCl, 25 mM CaCl_2_). The cells were stained with 5 μL of Annexin V-FITC and 5 μL of PI (propidium indole) to each tube. The cells were gently vortexed and incubated in the dark at room temperature for 15 min and then keep them at 4 °C. The samples were analysed by a flow cytometer (Becton-Dickinson FACSCalibur, Totowa, NJ, USA) and data were analysed using the FlowJo software.

#### 2.7.4. Cell cycle analysis

Cell cycle distributions in HeLa cells were determined through PI staining and analysed by flow cytometry^37^. HeLa cells were seeded into a six-well plate at 5 × 10^5^ cell/mL and treated with different concentrations of compound **4d** for 48 h. After treatment, cells were detached with 0.25% trypsin, harvested by centrifugation, washed twice with ice-cold PBS and then fixed and permeabilised with ice-cold 70% ethanol at 4 °C overnight. Ethanol was removed and the cells were washed twice with ice-cold PBS. After this, the cells were treated with 100 μL of RNase (100 μg/mL) at 37 °C for 30 min, followed by incubation with 400 μL of DNA staining solution propidium iodide (PI) (1 mg/mL) in the dark at 4 °C for 30 min. The samples were analysed by a flow cytometer (Becton-Dickinson FACSCalibur, Totowa, NJ, USA) and data were analysed using the FlowJo software (Becton-Dickinson & Co, Totowa, NJ, USA).

#### 2.7.5. ROS generation assay

ROS generation assay was performed by using the reactive oxygen species assay kit (Beyotime Biotech., Nantong, China). Intracellular ROS generation was tested through dichlorodihydro fluorescein diacetate (DCFH-DA) assay^38^. DCFH-DA is taken up by HeLa cells, and then activated by esterase-mediated cleavage of acetate to form DCFH, which is trapped in the cells. DCFH is converted to fluorescein DCF in the presence of ROS. HeLa cells were seeded in six-well plates and incubated with different concentrations of compound **4d** for 48 h. After removing the compound solution, cells were treated with 10 μM of DCFH-DA at 37 °C for 20 min. Subsequently, the cells were washed with PBS for three times and then exposed to light. Immediately after light exposure, the fluorescence intensity of dichlorofluorescein (DCF) was measured with excitation at 488 nm and emission at 525 nm by a flow cytometry (Becton-Dickinson FACSCalibur, Totowa, NJ, USA).

#### 2.7.6. JC-1 mitochondrial membrane potential assay

The JC-1 mitochondrial membrane potential assay kit (Keygene Biotech., Nanjing, China) was employed to measure mitochondrial depolarisation in HeLa cells. Briefly, cells cultured in six-well plates after indicated treatments by compound **4d** were incubated with an equal volume of JC-1 staining solution (5 μg/mL) at 37 °C for 20 min and rinsed twice with PBS. Mitochondrial membrane potentials were monitored by determining the relative amounts of dual emissions from mitochondrial JC-1 monomers or aggregates using flow cytometry (Becton-Dickinson FACSCalibur, New York, NY, USA). Mitochondrial membrane depolarisation is indicated by an increase in the percentage of cells with low ΔΨm (green fluorescence and lower right quadrant) compared with cells with high ΔΨm (red fluorescence and upper right quadrant).

#### 2.7.7. Western blot analysis

HeLa cells were seeded at a density of 5 × 10^6^ cells per well and attached for 8 h, and then treated with different concentrations of compound **4d** for 48 h. After the treatment, the cells were harvested and washed twice with PBS. The harvested cells were lysed with radio-immunoprecipitation assay (RIPA) lysis buffer (Beyotime Biotech., Nantong, China) with 1% cocktail (Sigma-Aldrich, St. Louis, MO, USA). Whole-cell protein lysates were prepared and centrifuged at 12,000 rpm for 10 min at 4 °C. The total proteins were determined using Bradford reagent (Bio-Rad Laboratories, Inc., Hercules, CA, USA). Exactly 40 μg of protein per lane was separated through sodium dodecyl sulphate-polyacrylamide gel electrophoresis and then transferred onto a polyvinylidene difluoride membrane (Millipore, Bedford, MA, USA). The membranes were incubated with each antibody and detected through immunoblot analysis. All of the antibodies were purchased from Cell signalling Technology, Inc. (Boston, MA, USA) and diluted in accordance with the manufacturer’s instruction. Proteins were visualised using a C-Digit^®^ imaging system (LI-COR, Lincoln, NE, USA).

#### 2.7.8. *In vitro* MEK1 kinase assay

An *in vitro* kinase assay of MEK1 was performed using ADP-Glo kinase assay (Promega, Madison, WI, USA) according to the manufacturer’s protocol. Briefly, the kinase reaction was conducted in a 5 μL mixture [25 mM Tris-HCl (pH 7.5), 25 mH MgCl_2_, 2 mM dithiothreitol, 10 μM ATP, 0.02% Triton X-100, 200 ng of recombinant GST-MEK1 protein (Active) and 200 ng of GST-ERK2 (Inactive) protein (Carna Biosciences, Kobe, Japan)] with or without various concentrations of tested compounds at 22 °C for 30 min. Reactions were stopped by adding 5 μL of ADP-Glo reagent to each well. After incubating at 22 °C for 40 min, 10 μL of the kinase detection reagent was added and the plates were incubated for another 30 min at 22 °C in the dark. The reaction mixture was analysed by EnSpire (PerkinElmer, Waltham, MA, USA). AZD6244 was used as the positive control for MEK1 inhibition.

### 2.8. Molecular docking

The molecular modelling of compound **4d** was performed with Schrödinger Suite 2015-1 (Schrödinger LLC., New York, NY, USA)^39^. The crystal structure of the MEK1 (PDB ID: 3EQF) was downloaded from Protein Data Bank (PDB) and prepared using the Protein Preparation Wizard workflow from Schrödinger Suite, including the optimisation of hydrogen bond network and a short energy minimisation with position restraints on heavy atoms using OPLS_2005 force field. The docking grid was generated according to the initial ligand K252A. Then the target compounds were freely docked into the designated binding site using the standard protocol implemented in Maestro version 10.1 (Schrodinger LLC, Cambridge, MA, USA). Van der Waals (vdW) scaling of 0.8 and partial cut-off of 0.15 were set to soften the potential for non-polar sites, and no constraints were specified. The best docked pose ranked by Glide Score value was recorded, and saved for each ligand. The structures of complexes were analysed for interaction modes, and the binding pose of compound **4d** with MEK1 kinase was displayed using Discovery studio 3.5 client.

## 3. Results and discussion

### 3.1. Chemistry

**Scheme 1.**
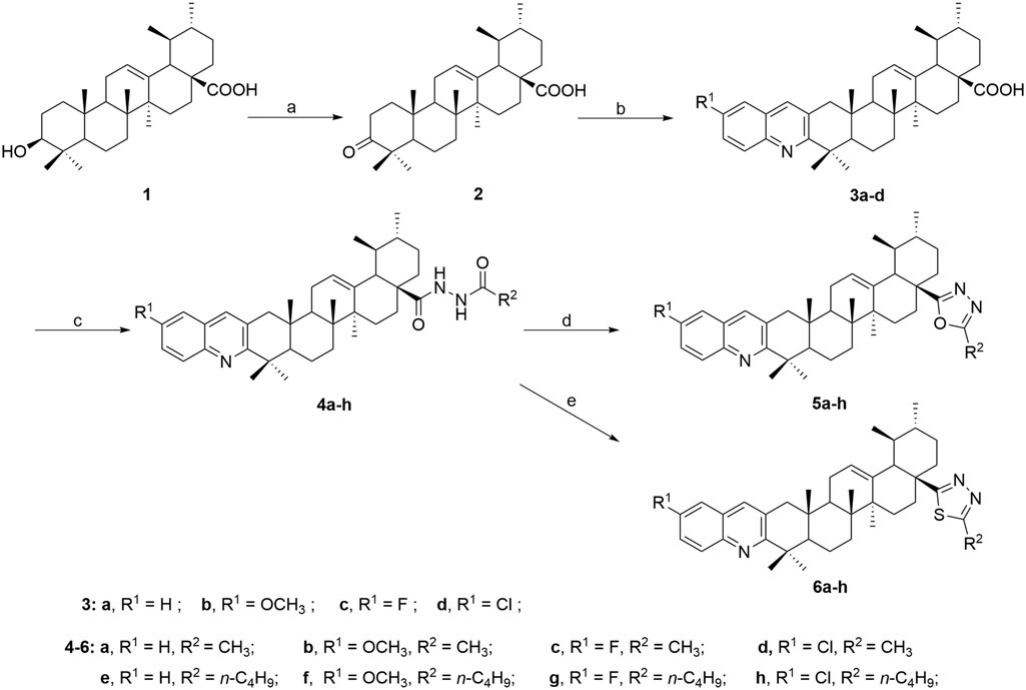
Synthetic procedure to target compounds **4a–h**, **5a–h,** and **6a–h** from ursolic acid. Reagents and conditions: (a) Jones reagent, acetone, 0 °C, 5 h; (b) EtOH, substituted *o*-amino benzaldehyde, KOH, reflux under N_2_ atmosphere for 24 h; (c) i. SOCl_2_, benzene, reflux for 3 h; ii. RCONHNH_2_, Et_3_N, CH_2_Cl_2_/ether, rt, 8 ∼ 12 h; (d) TsOH, toluene, reflux for 6 h; (e) Lawesson reagent, toluene, 110 °C, 6 h.

The target compounds were synthesised according to the procedure reported earlier^34^ as outlined in Scheme 1. In brief, UA (**1**) was oxidised with Jones reagent to form 3-oxo-ursolic acid (**2**) in 79% yield. Through Friedlander synthesis, compound **2** was further reacted with different *o*-aminobenzaldehyde under nitrogen atmosphere to afford the corresponding quinoline derivatives **3a–d** in 62 ∼ 68% yields. Concerning the modification of carboxyl group, compounds **3a–d** were treated with thionyl chloride to give the 28-acylchloride derivatives, which were then reacted with acethydrazide or valerohydrazide in the presence of Et_3_N to obtain compounds **4a–h**. Furthermore, oxadiazole derivatives **5a–h** were synthesised through dehydration condensation of compounds **4a–h** in the presence of *p*-toluenesulfonic acid in 53 ∼ 78% yields, while the thiadiazole derivatives **6a–h** were obtained by treating compounds **4a–h** with Lawesson reagent in 57 ∼ 72% yields. All the synthesised compounds (**4a–h**, **5a–h,** and **6a–h**) were purified by silica gel column chromatography and their structures were characterised through ^1^H NMR, ^13 ^C NMR (Supplementary Figures S1∼S48), IR, and HR-MS spectral data.

### 3.2. Biological evaluation

#### 3.2.1. *In vitro* cytotoxic assay

The *in vitro* cytotoxic activity of derivatives **3a–d**, **4a–h**, **5a–h,** and **6a–h** were evaluated by MTT assay against human breast cancer cell line (MDA-MB-231), cervical carcinoma cell line (HeLa), hepatocarcinoma cell line (SMMC-7721) and human normal hepatocyte cell line (QSG-7701). The anticancer drug etoposide was co-assayed as the positive control. All tested compounds were dissolved in DMSO and the stock solutions were diluted by DMEM medium before treatment of the cultured cells. The IC_50_ values of the tested compounds against four cell lines are shown in Table 1.

**Table 1.** The *in vitro* cytotoxic activities of the tested compounds (**1–2**, **3a–d**, **4a–h**, **5a–h,** and **6a–h**) against three human cancer cell lines (SMMC-7721, MDA-MB-231, and HeLa) and one normal hepatocyte cell line (QSG-7701).

Compound	IC_50_ (μM)				Selectivity^b^
	MDA-MB-231	HeLa	SMMC-7721	QSG-7701	
**1**	>50	>50	>50	NT^a^	NT
**2**	>50	>50	>50	NT	NT
**3a**	0.75 ± 0.05	0.37 ± 0.04	13.40 ± 0.08	>50	145.2
**3b**	0.61 ± 0.07	0.36 ± 0.05	12.49 ± 0.08	>50	142.3
**3c**	0.90 ± 0.10	1.87 ± 0.03	13.34 ± 0.13	>50	37.0
**3d**	1.36 ± 0.03	1.22 ± 0.08	14.62 ± 0.05	>50	59.5
**4a**	1.84 ± 0.13	1.18 ± 0.03	17.48 ± 0.10	40.59 ± 2.89	34.4
**4b**	1.42 ± 0.14	0.83 ± 0.09	17.65 ± 0.11	45.20 ± 2.82	54.5
**4c**	1.16 ± 0.06	0.99 ± 0.05	19.41 ± 0.12	>50	63.8
**4d**	0.12 ± 0.01	0.08 ± 0.01	0.34 ± 0.03	10.76 ± 0.72	134.5
**4e**	>50	>50	>50	NT	NT
**4f**	>50	>50	>50	NT	NT
**4g**	>50	>50	>50	NT	NT
**4h**	>50	46.01 ± 0.91	>50	NT	NT
**5a**	5.32 ± 0.13	19.44 ± 0.70	>50	>50	4.3
**5b**	12.25 ± 0.12	16.76 ± 0.34	>50	>50	5.6
**5c**	13.17 ± 0.58	33.84 ± 0.95	>50	>50	3.6
**5d**	10.95 ± 0.98	4.28 ± 0.23	>50	>50	20.1
**5e**	>50	>50	>50	NT	NT
**5f**	>50	30.94 ± 1.14	>50	NT	NT
**5g**	>50	>50	>50	NT	NT
**5h**	>50	>50	>50	NT	NT
**6a**	18.75 ± 1.38	27.31 ± 1.91	>50	>50	4.8
**6b**	15.66 ± 2.01	12.82 ± 1.32	>50	>50	8.5
**6c**	31.57 ± 2.70	>50	>50	>50	2.8
**6d**	15.75 ± 1.32	10.92 ± 1.07	>50	>50	5.7
**6e**	>50	>50	>50	NT	NT
**6f**	>50	>50	>50	NT	NT
**6g**	>50	>50	>50	NT	NT
**6h**	>50	>50	>50	NT	NT
Etoposide	5.26 ± 1.21	2.98 ± 0.42	3.48 ± 0.35	28.75 ± 3.28	9.6

^a^NT: not tested.

^b^Selectivity: IC_50_ (QSG-7701)/IC_50_ (HeLa).

As illustrated in Table 1, the tested compounds displayed varying degrees of cytotoxic activity against the three cancer cell lines. Generally, these derivatives showed the strongest activities against HeLa cells, then MDA-MB-231 cells, and were least active to SMMC-7721 cells. Concerning different derivatives, it was found that compounds **3a–d** with carboxyl groups exhibited potent cytotoxic activities against MDA-MB-231 and HeLa cells at low μM levels and moderate activities against SMMC-7721 cells. Among acylhydrazine derivatives **4a–h**, compounds **4a–d** also exhibited significant cytotoxic activities comparable to compounds **3a–d**, while compounds **4e–h** were almost inactive to all three cancer cell lines (IC_50_ > 50 μM). Regarding oxadiazole derivatives **5a–h** and thiadiazole derivatives **6a–h**, compounds **5a** and **5d** showed strong cytotoxicity against MDA-MB-231 and HeLa cells, respectively (IC_50_ < 10 μM). Compounds **5 b**, **5c**, **6a**, **6 b,** and **6d** displayed moderate activities against MDA-MB-231 and HeLa cells, while compounds **5e–h**, **6c,** and **6e–h** only showed mild or no cytotoxicity against three cancer cell lines. It is worth noting that compound **4d** exhibited the most potent antiproliferative activity against all three cancer cells at low μM to nM range (IC_50_: 0.12 ± 0.01, 0.08 ± 0.01 and 0.34 ± 0.03 μM, respectively), stronger than positive control etoposide. In addition, compound **4d** was less cytotoxic to normal hepatocyte cells (QSG-7701) with IC_50_ value of 10.76 ± 0.72 μM, which indicated a high selectivity of cytotoxicity (134.5) between cancer cells and normal hepatocyte cells. Hence, compound **4d** was selected for further investigations on its anticancer mechanisms.

From above-mentioned results, some preliminary structural-activity relationships (SAR) could also be deduced. A number of derivatives (**3–6**) bearing quinoline heterocycles exhibited substantially stronger cytotoxic activity than compounds **1** and **2**, indicating that the introduction of quinoline moiety will improve the cytotoxicity of UA derivatives. Concerning R^1^ substituents on quinoline moieties, compound **3b** (R^1^ = OCH_3_) showed the strongest cytotoxic activity in series **3a–d**. However, for compounds **4a–h**, **5a–h,** and **6a–h**, compounds **4d**, **5d,** and **6d** (R^1^ = Cl) displayed the strongest activities in their own series. The results indicated that for R^1^ substituents, Cl atom was most beneficial to the cytotoxic activity of target derivatives. On the other hand, the substituents derived from the carboxyl group of UA also markedly affected the cytotoxic potencies. Generally, compounds **3a–d** with carboxyl groups and **4a–d** with hydrazide moieties showed substantially stronger cytotoxicity to three cancer cell lines than compounds **5a–d** with oxadiazole moieties, which were also more cytotoxic than compounds **6a–d** with thiadiazole moieties. Furthermore, compound **4d** exhibited the most potent activity among all title compounds. Therefore, the beneficial effects of these moieties to cytotoxic potency were as following order: hydrazide > carboxyl group > oxadiazole > thiadiazole. In addition, the antiproliferative activities of compounds **4a–d** (*R*^2^ = Me) were much stronger than those of compounds **4e–h** (*R*^2^ = *n*-Butyl). Similar results could also be observed for compounds **5a–h** and **6a–h**. The results indicated that the introduction of a large alkyl group on the side chain might decrease the cytotoxic potency of the target derivatives. These preliminary SAR analyses could give useful prompt for the further investigation of UA derivatives.

#### 3.2.2. Induced apoptosis by compound 4d

**Figure 2.**
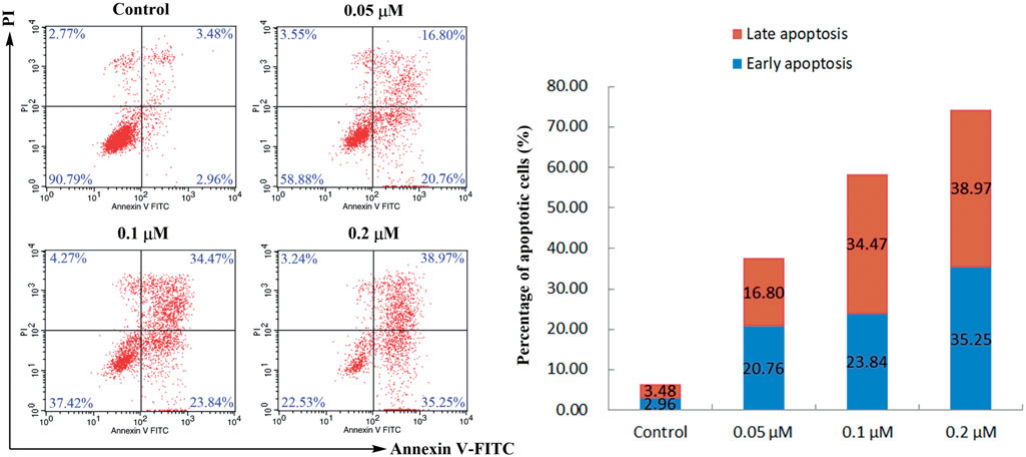
Annexin V-FITC/PI dual staining assay. HeLa cells were treated with different concentrations of compound **4d** (0, 0.05, 0.1, and 0.2 μM) for 48 h, stained with Annexin V-FITC/PI and analysed for apoptosis using flow cytometer. The percentage of cells positive for AV and/or PI is reported in the quadrants. Cells in the lower left quadrant (AV–/PI–): live cells; Lower right quadrant (AV+/PI–): early apoptotic cells; Upper right quadrant (AV+/PI+): late apoptotic cells; Upper left quadrant (AV–/PI+): necrotic cells.

The Annexin V-FITC/PI dual staining assay on HeLa cells treated with compound **4d** was carried out to examine the association of compound **4d**-mediated cytotoxicity with induction of apoptosis. As shown in Figure 2, after treating with different concentrations of **4d** (0.05, 0.1, and 0.2 μM) for 48 h, the percentage of early and late apoptotic cells (early apoptosis: Lower right quadrant, AV+/PI–; late apoptosis: Upper right quadrant, AV+/PI+) significantly increased from 6.44 to 37.56%, 58.31 and 74.22%, respectively. These results indicated that compound **4d** could trigger the apoptosis of HeLa cells in a concentration-dependent manner.

#### 3.2.3. Cell cycle analysis

**Figure 3.**
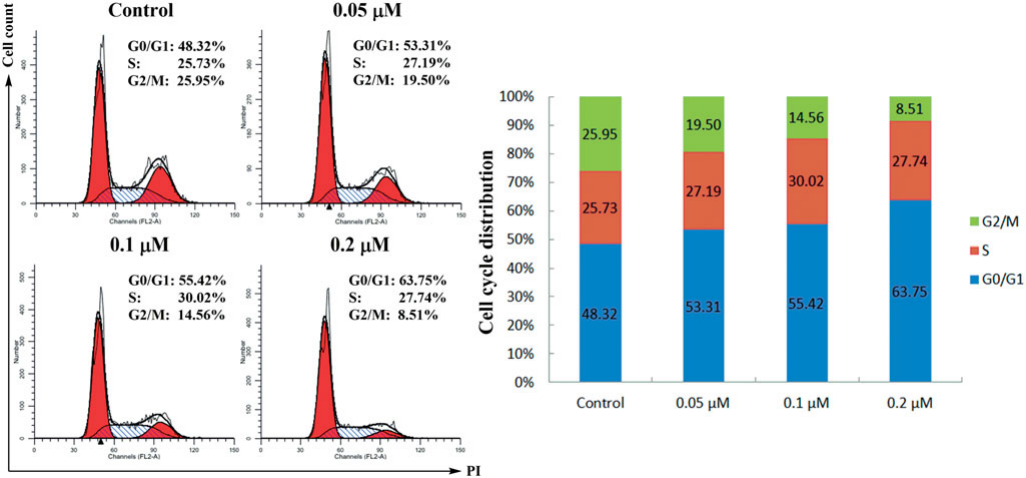
Cell cycle assay. HeLa cells were treated with different concentrations of compound **4d** (0, 0.05, 0.1, and 0.2 μM) for 48 h, stained with propidium iodide (PI) and analysed for cell cycle using flow cytometer.

Cell cycle distribution in HeLa cells was examined to determine whether or not compound **4d** inhibited the proliferation of these cells through cell cycle arrest. HeLa cells were treated with different concentrations of compound **4d** (0, 0.05, 0.1, and 0.2 μM) for 48 h. Cell cycle distribution was investigated by flow cytometric analysis after staining the DNA of the treated cells by PI. Cell cycle analysis demonstrated that treatment of compound **4d** concentration-dependently increased the population of cells in the G0/G1 phase. This phenomenon was accompanied by a decrease in the population of cells in G2/M phase. As shown in Figure 3, the population of HeLa cells in the G0/G1 phase increased from 48.32% (control group) to 63.75% (0.2 μM group), while the percentage of cells in the G2/M phase decreased from 25.95% (control group) to 8.51% (0.2 μM group). These data indicated that cell cycle arrest in the G0/G1 phase contributed to the anti-proliferative effects of compound **4d** on HeLa cells.

#### 3.2.4. ROS generation assay

**Figure 4.**
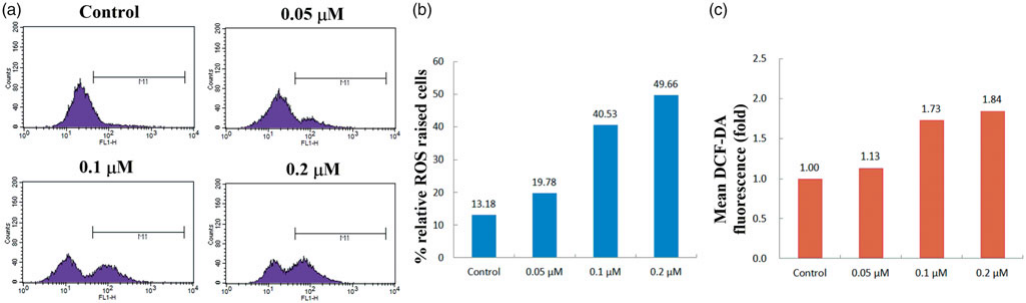
Effects of compound **4d** on the intracellular ROS level in HeLa cells. HeLa cells were treated with different concentrations of compound **4d** (0, 0.05, 0.1, and 0.2 μM) for 48 h, stained with DCFH-DA and analysed using flow cytometer.

ROS has been reported to be involved in early stages of apoptosis in many cellular systems. To determine whether the apoptosis was induced by the title compounds, the intracellular ROS level in compound **4d**-treated HeLa cells was also examined by using 2′,7′-dichlorodihydrofluorescein diacetate (DCFH-DA), which could be converted into a green fluorescent DCF by ROS oxidation^40^. HeLa cells were exposed to compound **4d** at 0 ∼ 0.2 μM concentrations for 48 h and the intracellular fluorescence intensity was quantitatively analysed by flow cytometry. As shown in Figure 4, upon treatment with different concentrations of compound **4d**, the percentage of cells with elevated ROS level increased from 13.18% (Control) to 49.66% (0.2 μM). In addition, the mean fluorescence intensity within cells treated with 0.2 μM of **4d** also increased by 84% compared to the control group. These results demonstrated that compound **4d** could induce a significant increase of ROS generation in HeLa cells in a dose-dependent manner. The elevated intracellular ROS levels indicated that cell apoptosis was correlated with the disruption of the balance between ROS generation and elimination^8^. Therefore, ROS production was responsible for apoptosis induced by compound **4d**.

#### 3.2.5. JC-1 mitochondrial membrane potential assay

As a kind of important organelle, Mitochondria play indispensable roles not only on supplying metabolic energy in the form of ATP, but also on regulating the signal transmission during the apoptosis of cancer cells^41^. In addition, the mitochondrial dysfunction can be aroused under high ROS exposure, leading to the collapse of mitochondrial membrane potential (ΔΨ_m_), which is a characteristic phenomenon of early apoptosis^42^. Loss of ΔΨ_m_ can be detected using the fluorescent cationic dye JC-1, which spontaneously forms red fluorescent dimers (J aggregates) under high ΔΨ_m_, whereas its monomeric form showing green fluorescence is prevalent in cells with low ΔΨ_m_. Green and red fluorescence can be observed at 527 and 590 nm, respectively. Thus, changes in the green/red fluorescence ratio reflect the variation in ΔΨ_m_, which can be quantitatively analysed by flow cytometry^43^.

**Figure 5.**
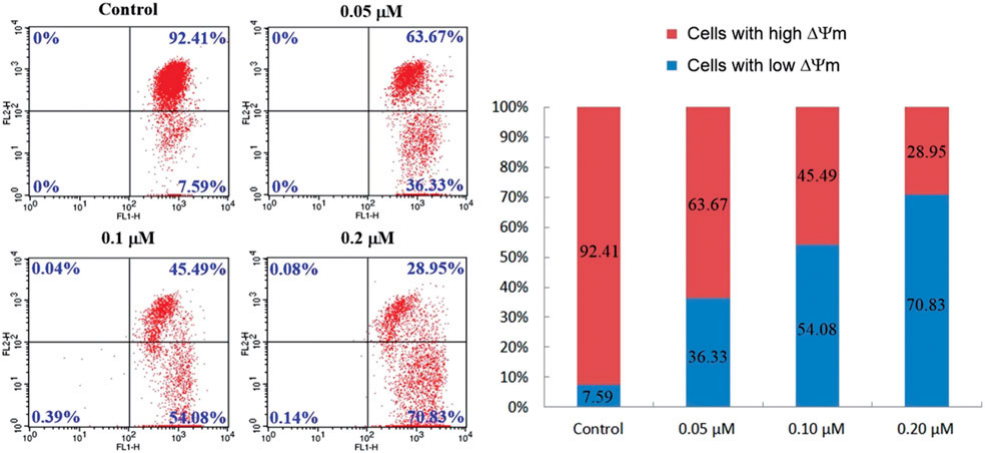
Compound **4d**-induced loss of the mitochondrial membrane potential (ΔΨ_m_). HeLa cells were treated with compound **4d** (0, 0.05, 0.1, and 0.2 μM) for 24 h, incubated with JC-1 and analysed using flow cytometry.

As shown in Figure 5, strong red fluorescence (Upper right quadrant) was detected in control cells, suggesting a high ΔΨ_m_. However, the green fluorescence indicating the loss of ΔΨ_m_ (Lower right quadrant) in compound **4d**-treated HeLa cells increased from 7.59% (control group) to 36.33% (0.05 μM group), 54.08% (0.1 μM group) and 70.83% (0.2 μM group), which indicated that compound **4d** could cause the decrease of mitochondrial membrane potential in a concentration-dependent manner. ROS production decreases ΔΨ_m_, destroys mitochondrial membrane integrity, and cause mitochondrial dysfunction^44^. The above results verified that elevated ROS level and ΔΨ_m_ loss led to mitochondrial damage, which was an important factor responsible for compound **4d**-induced apoptosis.

#### 3.2.6. Western blot analysis

It is well-known that anticancer drugs can stimulate apoptotic signalling through two major pathways. One is the death receptor (extrinsic) pathway involving the Fas ligand binding to Fas receptors. Another apoptotic pathway is the mitochondrial (intrinsic) pathway which is activated by the release of pro-apoptotic factors from mitochondria inter-membrane space, such as cytochrome c^45^. Mitochondria play an important role in cell death by changing its outer and inner membrane permeability and thus leading to cytochrome c release and caspases activation^46^.

**Figure 6.**
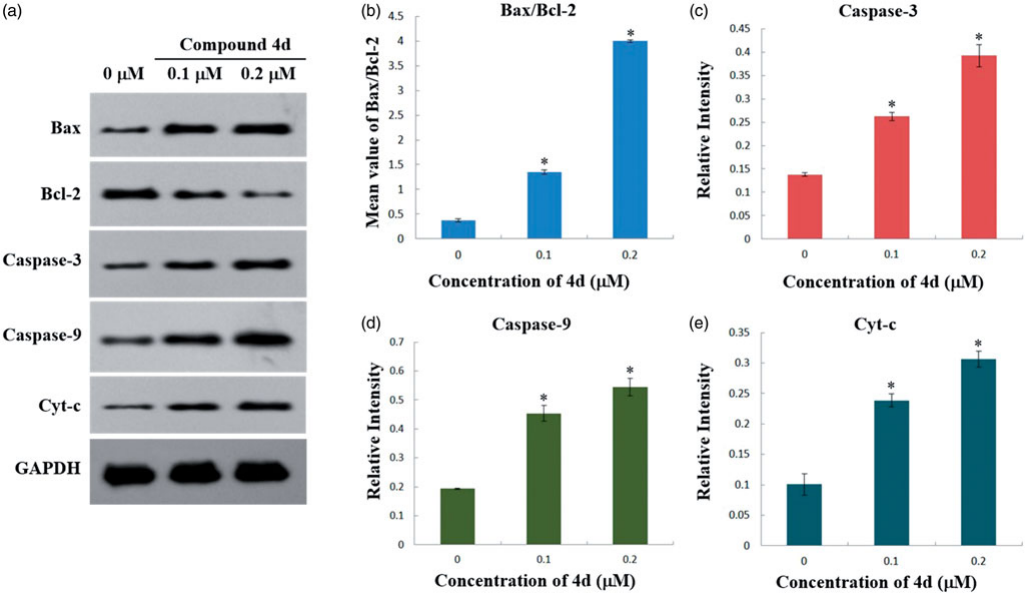
(a) Western blot analysis of the levels of Cytochrome c in cytosol, Bax, Bcl-2, Caspase-3, and Caspase-9 expression in HeLa cells treated by compound **4d** (0, 0.1 and 0.2 μM) for 48 h; (b) The rate changes of Bax/Bcl-2 in HeLa cells. *p < .001; (c) The expression level of cleaved caspase-3 in HeLa cells. *p < .001; (d) The expression level of cleaved caspase-9 in HeLa cells. *p < .02; (e) The expression level of Cytochrome c in cytosol of HeLa cells. *p < .01.

To further explore whether compound **4d** induced apoptosis *via* the mitochondrial signalling pathway, a number of key protein markers involved in mitochondria-mediated apoptosis were examined by Western blot analysis. The Bcl-2 family members are important regulators of the mitochondrial apoptotic pathway. Two most important members of Bcl-2 family, the anti-apoptotic protein Bcl-2 and the pro-apoptotic protein Bax, are key regulators of this progress^47^. As shown in Figure 6(a), the expression level of Bax was elevated in HeLa cells compared with the control group, whereas the expression level of Bcl-2 was decreased after treatment with compound **4d** for 48 h. Therefore, the ratio of Bax/Bcl-2 (Figure 6(b)) increased in a dose-dependent manner. Moreover, cytochrome c is reported as a key protein involved in the activation of the downstream caspases that trigger apoptosis. Caspases are a family of cysteinyl aspartate-specific proteases involved in apoptosis, which can be classified into groups of initiators (caspases 8, 9, and 10) and executioners (caspases 3, 6, and 7)^48^^,^^49^. In this assay, we also examined the roles of cytochrome c, caspase-3, and caspase-9 in the cellular response to compound **4d**. It was observed that with the treatment of compound **4d** (0, 0.1 and 0.2 μM), the levels of caspase-3, caspase-9, and cytochrome c of HeLa cells all significantly increased in a concentration-dependent manner (Figure 6(c–e)). Taken together, these data suggested that compound **4d** could induce the apoptosis of HeLa cells through mitochondrial signalling pathway.

#### 3.2.7. MEK inhibitory activity

The Raf/MEK/ERK pathway is the downstream of Ras activation, and the abnormal expression or phosphorylation of the associated proteins is essential for cancer cell proliferation^50^. In this pathway, MEK kinase is an important anticancer target because of its key roles in regulating the cancer cell apoptosis and oncogenic transformation^51^^,^^52^. Among MEK proteins, MEK1 and MEK2 are known as substrate-specific kinases which phosphorylate their downstream kinase ERK1/2. Upon inhibition, the phosphorylation of ERK1/2 will be significantly suppressed. As inhibition of the phosphorylation of ERK has been proposed as a primary biomarker of MEK inhibitory activity^53^, the MEK1 inhibitory activities of some compounds exhibiting good cytotoxicity were examined by an *in vitro* kinase assay of recombinant MEK1 using GST-ERK2 (Inactive) as a substrate^54^. The IC_50_ values of the tested compounds against MEK1 are shown in Table 2.

**Table 2.** MEK1 inhibitory activity of UA derivatives.

Compound	IC_50_ (μM)
**3a**	18.55
**3b**	3.59
**3c**	0.37
**3d**	0.82
**4a**	37.28
**4b**	10.91
**4c**	0.072
**4d**	0.064
**5a**	>50
**5d**	>50
**6d**	>50
AZD6244	0.039

As a result, compound **4d** exhibited the most potent inhibitory activity against MEK1 with IC_50_ value of 0.064 μM, competitive to positive control AZD6244. Compounds **4c**, **3c,** and **3d** also showed significant inhibitory activities with IC_50_ values of 0.072, 0.37 and 0.82 μM, respectively. Compounds **3a**, **3 b**, **4a,** and **4 b** showed moderate to weak inhibition to MEK1 kinase, while **5a**, **5d,** and **6d** were inactive to MEK1 (IC_50_ > 50 μM). It could be observed that compounds with carboxyl groups or hydrazide moieties showed stronger inhibition to MEK1 than compounds with oxadiazole or thiadiazole moieties. As for substituents on quinoline rings, the F and Cl substituents seems to be more beneficial to MEK1 inhibitory activity than H and OCH_3_. In general, compounds with the most antiproliferative activities also exhibited potent MEK1 inhibitory activities, which indicated that MEK1 kinase inhibition probably played an important role for the anticancer activity of these compounds.

**Figure 7.**
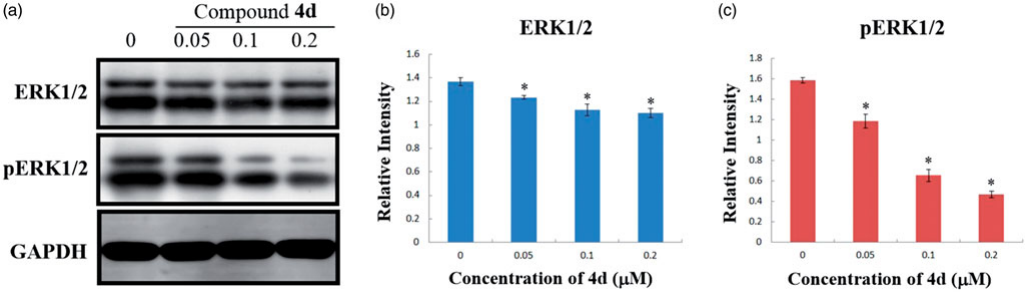
(a) Effects of compound **4d** on the expression of ERK and pERK in HeLa cells. HeLa cells were treated with compound **4d** (0, 0.05, 0.1, and 0.2 μM) for 48 h; (b) The expression level of ERK1/2 in HeLa cells. *p < .001; (c) The expression level of pERK1/2 in HeLa cells. *p < .001.

Subsequently, to evaluate the MEK inhibition of compound **4d** in cancer cells, western blot analyses were also carried out to evaluate the expression levels of ERK and phosphorylated ERK (pERK) proteins in compound **4d**-treated HeLa cells. As exhibited in Figure 7(a), the expression levels of ERK1/2 slightly decreased. More importantly, the expression levels of pERK1/2 were significantly downregulated by compound **4d** in a dose-dependent manner. After treatment with different concentrations of **4d** (0.05, 0.1, and 0.2 μM) for 48 h, the expression level of pERK1/2 was reduced to 74.7, 41.3, and 29.5% of the control group, respectively. Therefore, these immunoblot results indicate that compound **4d** can strongly inhibit MEK catalytic activity, thus can suppress the phosphorylation and activation of the downstream target ERK.

**Figure 8.**
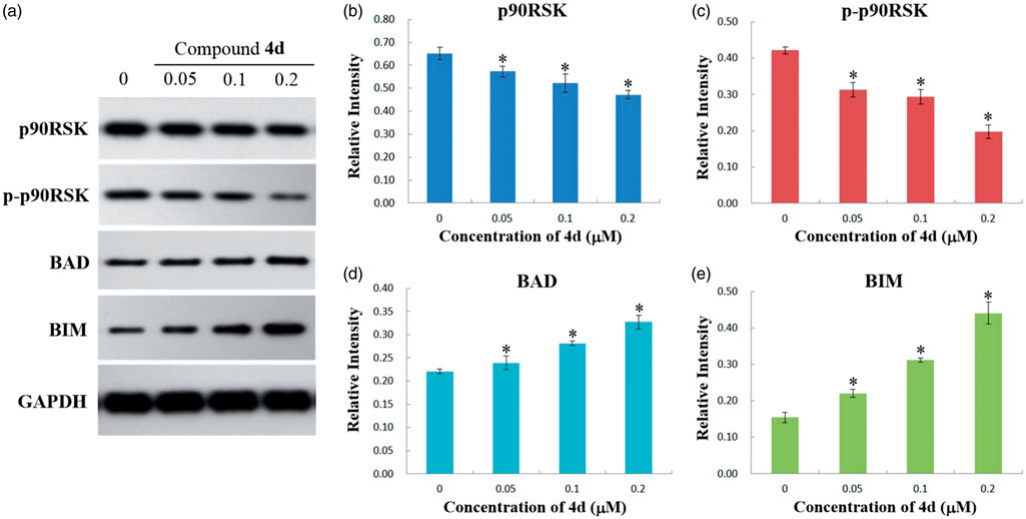
(a) Western blotting analysis of the levels of p90RSK, p-p90RSK, BAD, and BIM expression in HeLa cells treated by compound **4d** (0, 0.05, 0.1, and 0.2 μM) for 48 h; (b) The expression level of p90RSK in HeLa cells. *p < .01; (c) The expression level of p-p90RSK in HeLa cells. *p < .001; (d) The expression level of BAD in HeLa cells. *p < .001; (e) The expression level of BIM in HeLa cells. *p < .001.

In addition, the effect of compound **4d** on the downstream proteins of ERK (p90RSK, BAD, and BIM) was also investigated by western blot analysis. It is known that activated ERK1/2 (p-ERK1/2) directly phosphorylates and activates p90RSK, which subsequently decreases the level of pro-apoptotic BH3-only proteins BIM and BAD and inhibits their apoptosis-promoting activity^55^. As shown in Figure 8, the expression level of p90RSK, and p-p90RSK in HeLa cells was significantly decreased by compound **4d** in a concentration-dependent manner. On the contrary, the level of BAD and BIM were upregulated along with the increasing concentrations of compound **4d** (0, 0.05, 0.1, and 0.2 μM). These results suggested that the inhibition of MEK kinase activity could lead to the upregulation of the pro-apoptotic BIM and BAD proteins, and exert its anti-proliferative activity by promoting apoptosis signalling pathway.

#### 3.2.8. Molecular docking

**Figure 9.**
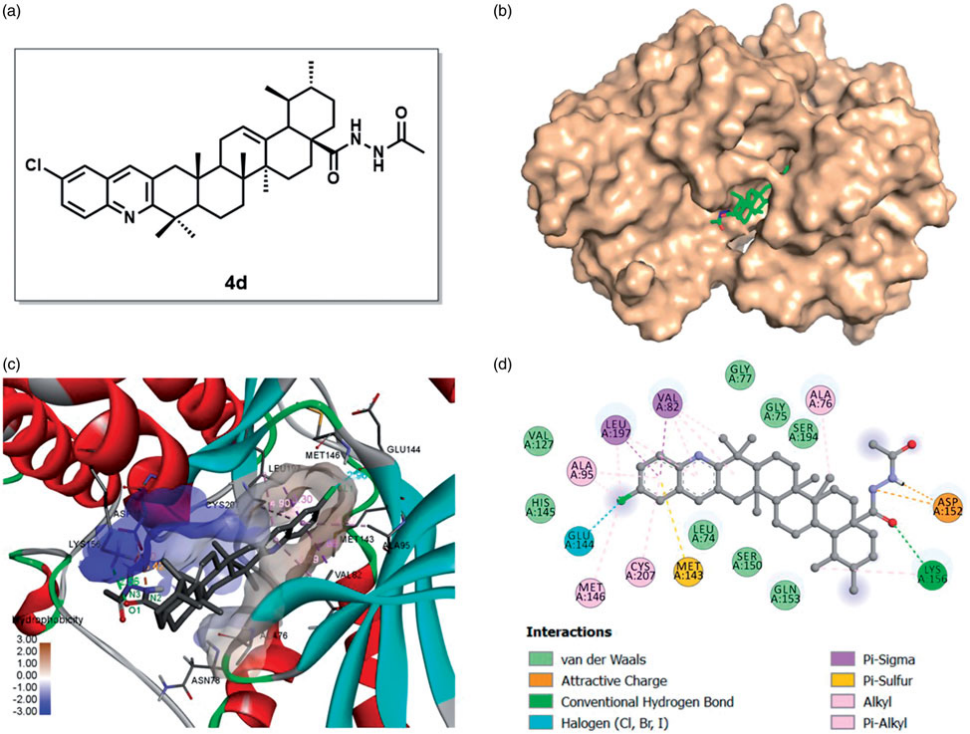
Binding mode of compound **4d** at MEK kinase domain (PDB: 3EQF). (a) Molecular structure of compound **4d**; (b) Space-filling model of MEK1 protein with compound **4d** embedded in the binding pocket; (c) Binding pose of compound **4d** within the MEK1 kinase domain. Ligand and key residues are presented as stick models and coloured by atom type, whereas the proteins are represented as ribbons. The colours of the surface indicate the hydrophobicity of the binding pocket. The dash lines exhibit the H-bond and other non-bond interactions; (d) 2D projection drawing of compound **4d** docked into MEK1 active site.

To gain more understanding of the interaction between target compound **4d** and MEK, we explored their binding modes generated by molecular docking based on the reported MEK1/inhibitor complex structure (PDB code: 3EQF). The docking studies were performed by using GLIDE docking module of Schrödinger suite 2015-1^39^ and the docking results were analysed and visualised by Discovery Studio Visualizer version 18.1.0^56^ (Accelrys Software, San Diego, CA, USA). The binding models of compound **4d** with the MEK1 structure are shown in Figure 9.

Visual inspection of the pose of compound **4d** into the MEK-binding site revealed that it has suitable shape complementarity with the binding pocket, affording a significant docking score (–7.353), comparable to the docking score of AZD6244 (–7.401). Especially, the 4-chloro-quinoline moiety was deeply embedded into the pocket (Figure 9(b)), and extensive hydrophobic interactions were formed between quinoline ring and residues Val 82, Leu 197, Ala 95, Cys 207, and MET 143 in the hydrophobic part of the pocket. The chloro atom on the quinoline ring also presented alkyl hydrophobic interactions with residues Leu 197, Ala 95, Met 146, and halogen interaction with Glu 144. On the other hand, different interactions formed by the hydrazide side chain of compound **4d** with amino acid residues in binding site also played a vital role for the stabilisation of current binding mode. An H-bond interaction was established between O1 atom of **4d** and Lys 156 (O1⋅⋅⋅H – N/Lys 156, angle O⋅⋅⋅H – *N* = 108.7°, distance = 2.56 Å). And two electrostatic interactions were also detected between N2, N3(H) of **4d** and Asp 152 with the distances of 3.42 and 1.80 Å, respectively. The skeleton of UA also played an important role in the interactions between compound **4d** and the protein. The UA skeleton ensured a suitable shape of compound **4d** to dock into the active site, and thus the hydrazide and quinoline moieties could interact with the corresponding amino acid residues. In addition, the methyl groups at C-23, C-27, and C-29 of UA skeleton formed alkyl hydrophobic interactions with Val 82, Ala 76, and Lys 156, respectively. The molecule also formed vdW interactions with residues Gln 153, Ser 150, Leu 74, His 145, Val 127, Gly 77, Gly 75, and Ser 194 in the binding site of MEK1 (Figure 9(c,d)). According to the above, the molecular docking result along with the biological assay data suggested the potential of compound **4d** as a propitious MEK inhibitor appropriate for further investigation.

### 3.3. Possible anticancer mechanisms of compound 4d

**Figure 10.**
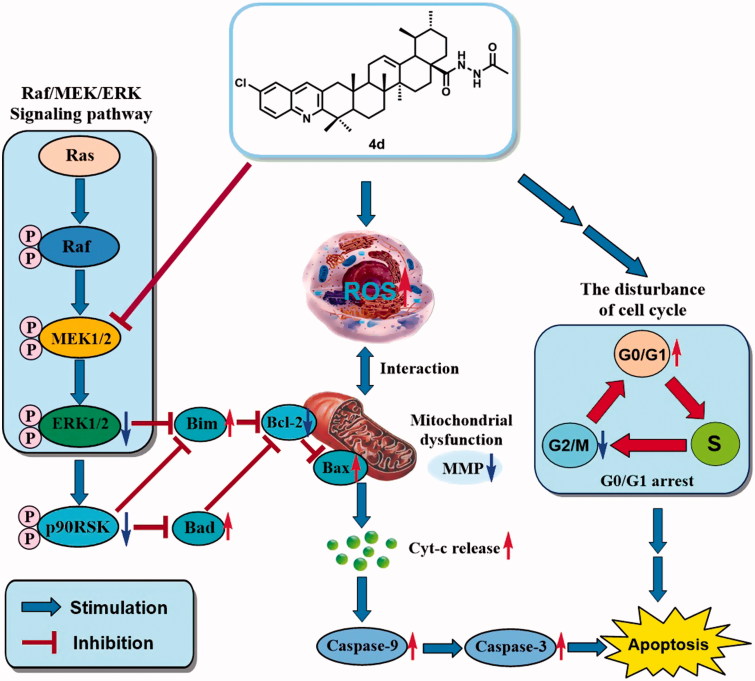
Possible anticancer mechanisms of compound **4d**.

Based on the aforementioned results, the putative mechanisms involved in the antiproliferative activity of compound **4d** against HeLa cells are summarised as follows (Figure 10). First, compound **4d** can elevate the intracellular ROS level, which causes the decrease of mitochondrial membrane potential (ΔΨ_m_), and upregulates the expression level of cyt-c, caspase-3, and caspase-9. Therefore, compound **4d** can induce the apoptosis of HeLa cells through a ROS-mediated mitochondrial pathway. Second, compound **4d** arrests the cell cycle of HeLa cells at G0/G1 phase, which is also closely correlated with the apoptosis event. Third, as a potent MEK kinase inhibitor, the compound can effectively hinder Ras/Raf/MEK/ERK signalling pathway. It is known that MEK1/2 phosphorylate and activate ERK1/2, which subsequently phosphorylate BIM, priming it for phosphorylation by p90RSK, and ultimately resulting in its ubiquitination and proteasomal degradation. ERK1/2 also directly activates p90RSK, which phosphorylates BAD on Ser112, facilitates its binding to 14–3-3. The protein 14–3-3 acts to sequester BAD and inhibit its pro-apoptotic activity^55^^,^^57^. Thus, the inhibition of MEK is expected to promote cell apoptosis by impeding the phosphorylation and activation of ERK and p90RSK, and subsequently increasing BIM and BAD levels. The upregulation of two pro-apoptotic proteins BIM and BAD can inhibit the activation of the anti-apoptotic protein Bcl-2 and increase the level of Bax, which promotes the downstream apoptotic signalling pathway^55^^,^^58^. However, the exact anticancer mechanisms of compound **4d** still remain unclear and need further explorations in the following study.

## 4. Conclusion

A series of novel quinoline derivatives of UA were designed, synthesised, and evaluated for their *in vitro* antiproliferative activities against three cancer cell lines (MDA-MB-231, HeLa, and SMMC-7721). Among them, several quinoline derivatives bearing carboxyl groups or hydrazide groups exhibited significant anticancer activities against three cancer cell lines, with IC_50_ values equivalent to or better than those of positive control etoposide. Especially, compound **4d** showed the most potent inhibitory activity against all the cancer cell lines and substantially lower cytotoxicity to the human normal hepatocytes QSG-7701. *In vitro* pharmacological analyses demonstrated that compound **4d** exerted its antiproliferative activity against HeLa cells by arresting cell cycle at the G0/G1 phase, inducing intracellular ROS generation, decreasing mitochondrial membrane potential, intervening with the Ras/Raf/MEK/ERK signalling pathway as MEK kinase inhibitor and finally inducing the apoptosis of HeLa cells. Molecular docking study also revealed that compound **4d** can effectively bind to the active site of MEK. All these results demonstrate the potential of this compound as a promising lead for the discovery of new anticancer drugs.

## Supplementary Material

Supplemental Material
