# Supplementary material for: Design, synthesis, and anticancer evaluation of novel quinoline derivatives of ursolic acid with hydrazide, oxadiazole, and thiadiazole moieties as potent MEK inhibitors
Source: J Enzyme Inhib Med Chem. 2019 May 9;34(1):955–72. doi: 10.1080/14756366.2019.1605364 (PMC6522941; doi:10.1080/14756366.2019.1605364)
Supplement: Supplemental Material [file IENZ_A_1605364_SM9447.pdf]

Supplementary material

Design, synthesis and anticancer evaluation of novel quinoline derivatives of ursolic acid with hydrazide, oxadiazole and thiadiazole moieties as potent MEK inhibitors

Xiao-Yan Jin, Hao Chen, Dong-Dong Li, A-Liang Li, Wen-Yan Wang, Wen Gu

Jiangsu Provincial Key Lab for the Chemistry and Utilization of Agro-forest Biomass, Jiangsu Key Lab of Biomass-based Green Fuels and Chemicals, Co-Inovation Center for Efficient Processing and Utilization of Forest Products, College of Chemical Engineering, Nanjing Forestry University, Nanjing 210037, P. R. China

Fig. S1~Fig. S48. ^{1}H and ^{13}C NMR spectra of compounds 4a-h, 5a-h and 6a-h.

Corresponding author.
Email address: njguwen@163.com (W. Gu)

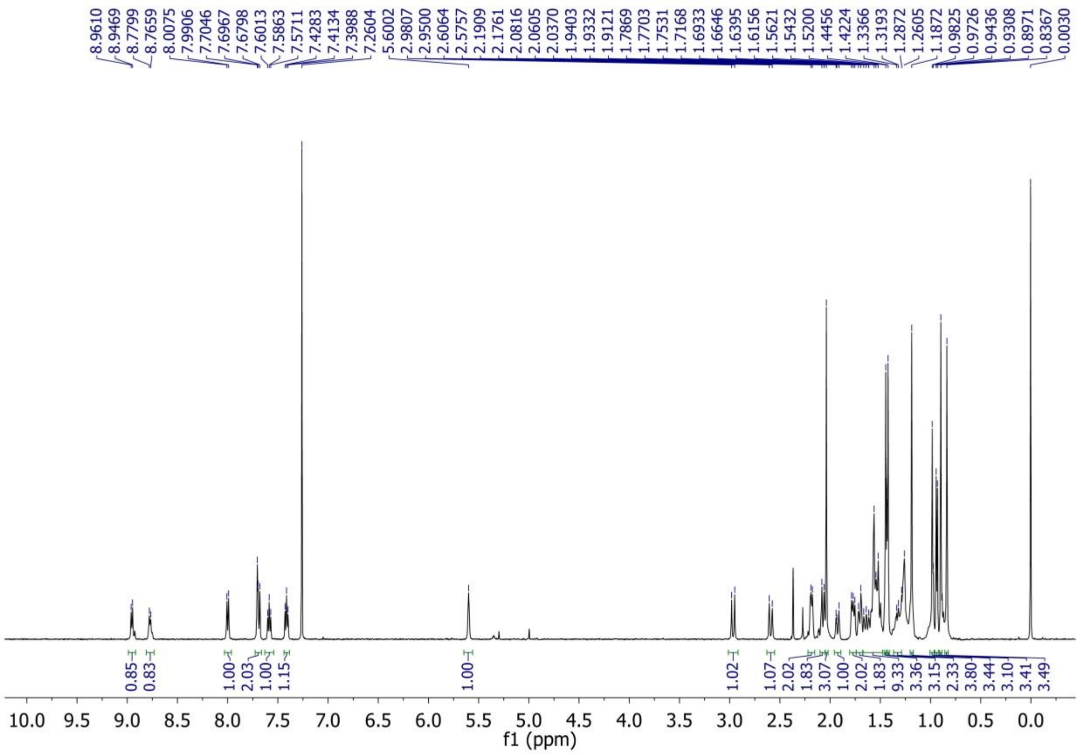

Fig. S1. ^{1}H-NMR spectrum of compound **4a** (500 MHz, $CDCl_3$).

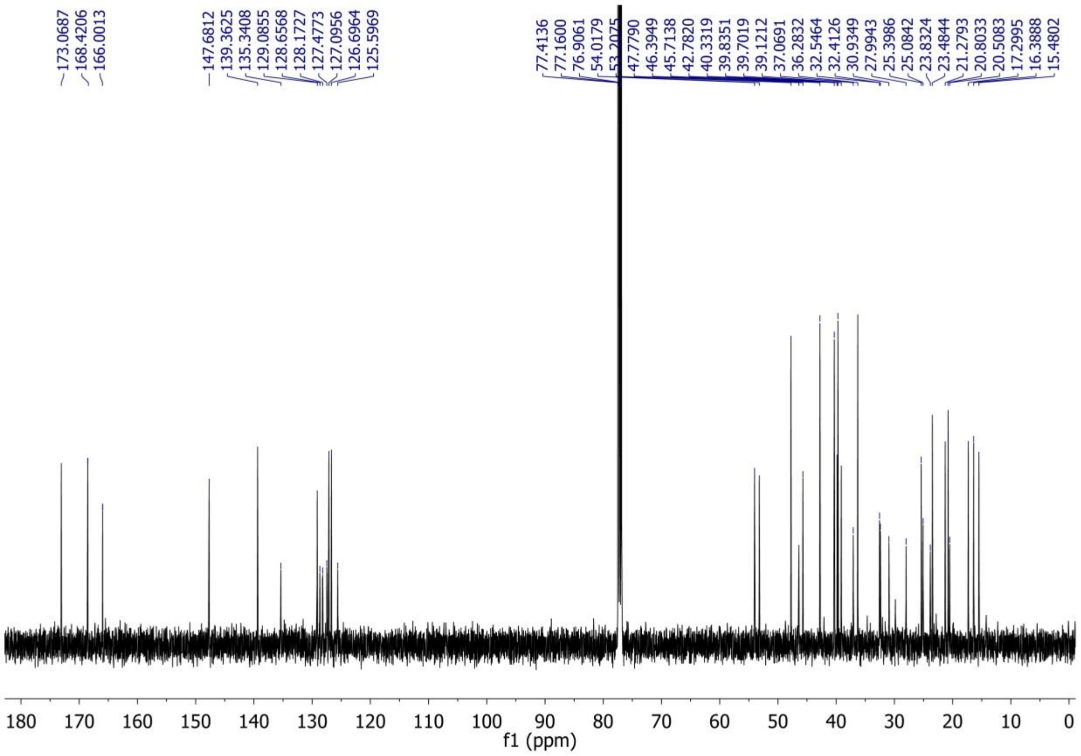

Fig. S2. ^{13}C-NMR spectrum of compound **4a** (125 MHz, $CDCl_3$).

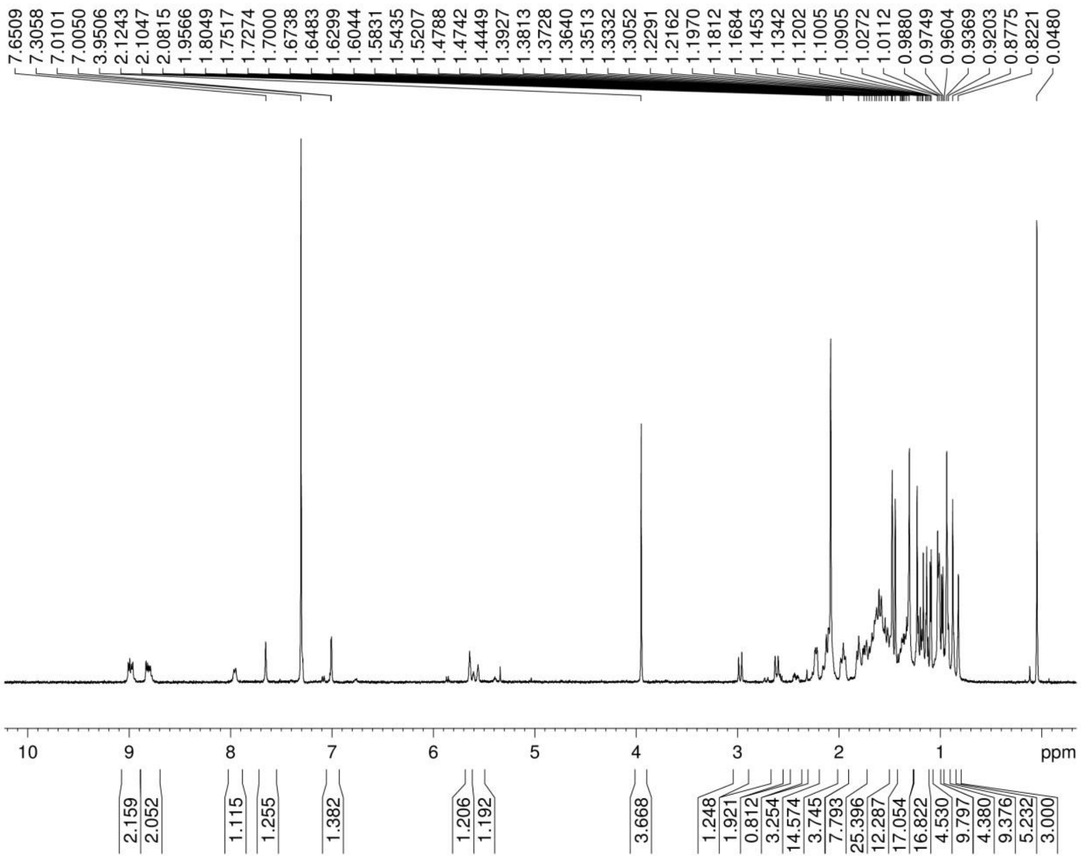

Fig. S3. ^{1}H-NMR spectrum of compound **4b** (500 MHz, $CDCl_3$).

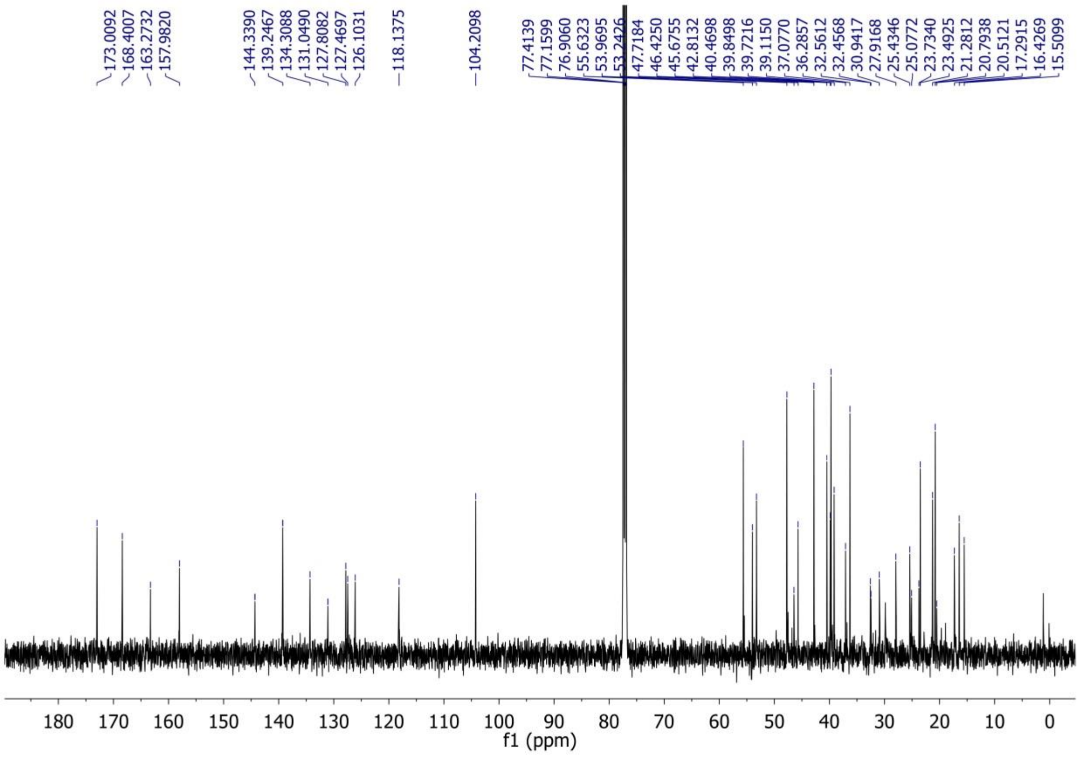

Fig. S4. ^{13}C-NMR spectrum of compound **4b** (125 MHz, $CDCl_3$).

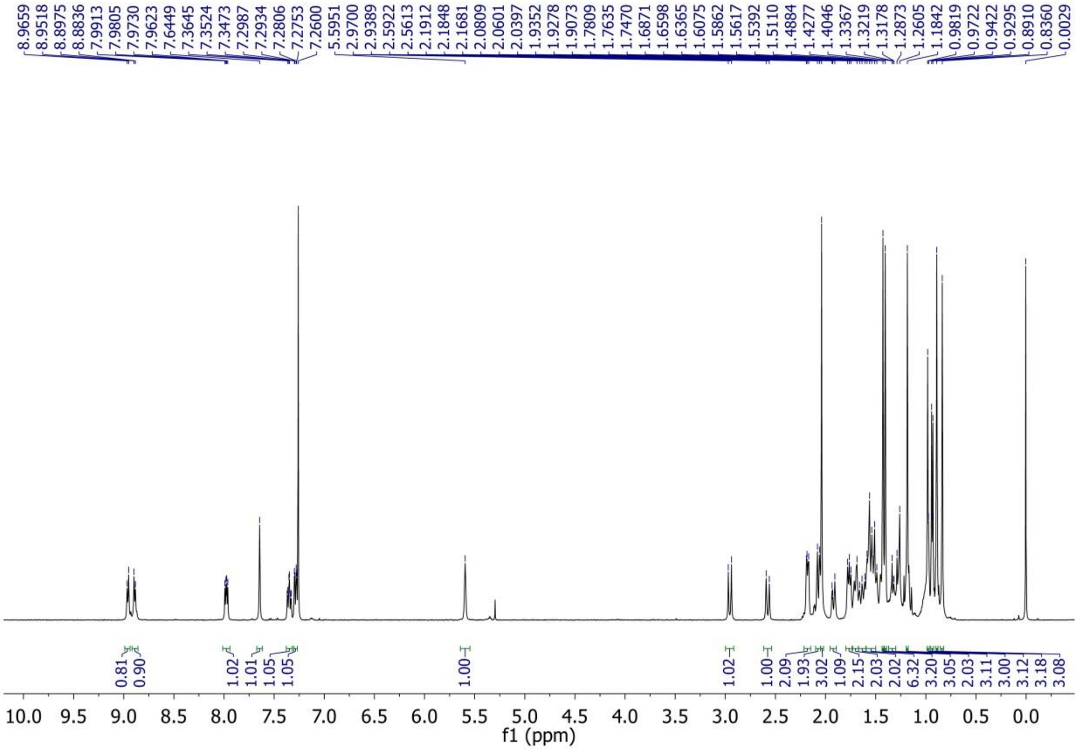

Fig. S5. ^{1}H-NMR spectrum of compound **4c** (500 MHz, $CDCl_3$).

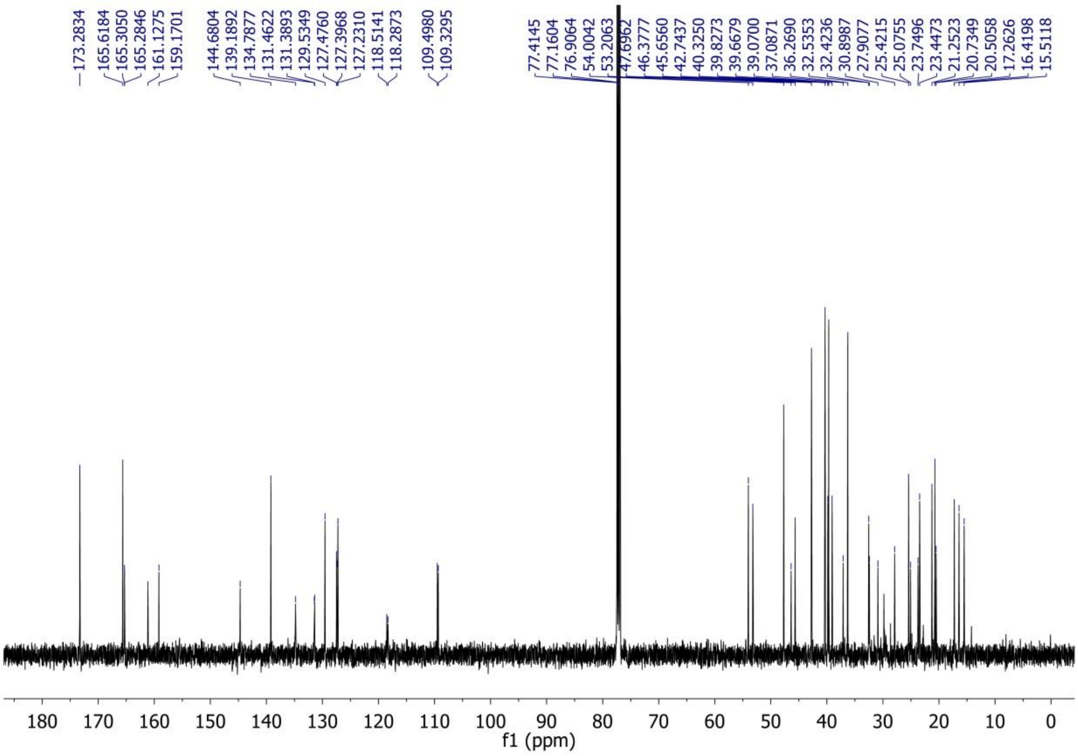

Fig. S6. ^{13}C-NMR spectrum of compound **4c** (125 MHz, $CDCl_3$).

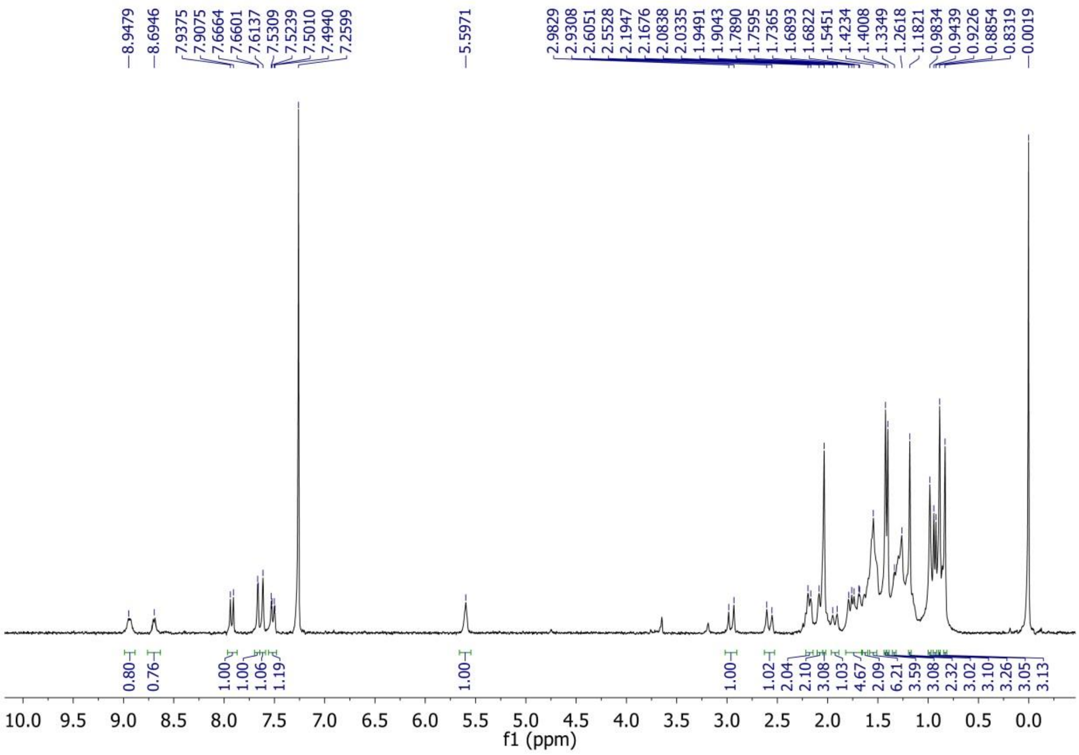

Fig. S7. ^{1}H-NMR spectrum of compound **4d** (500 MHz, $CDCl_3$).

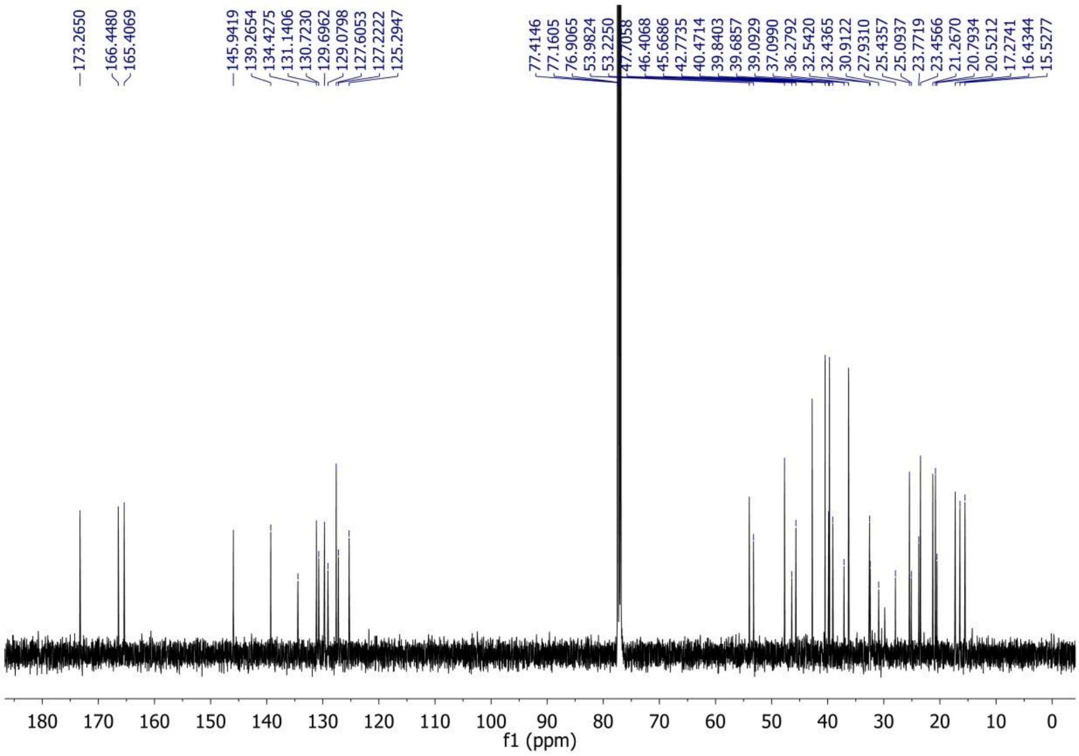

Fig. S8. ^{13}C-NMR spectrum of compound **4d** (125 MHz, $CDCl_3$).

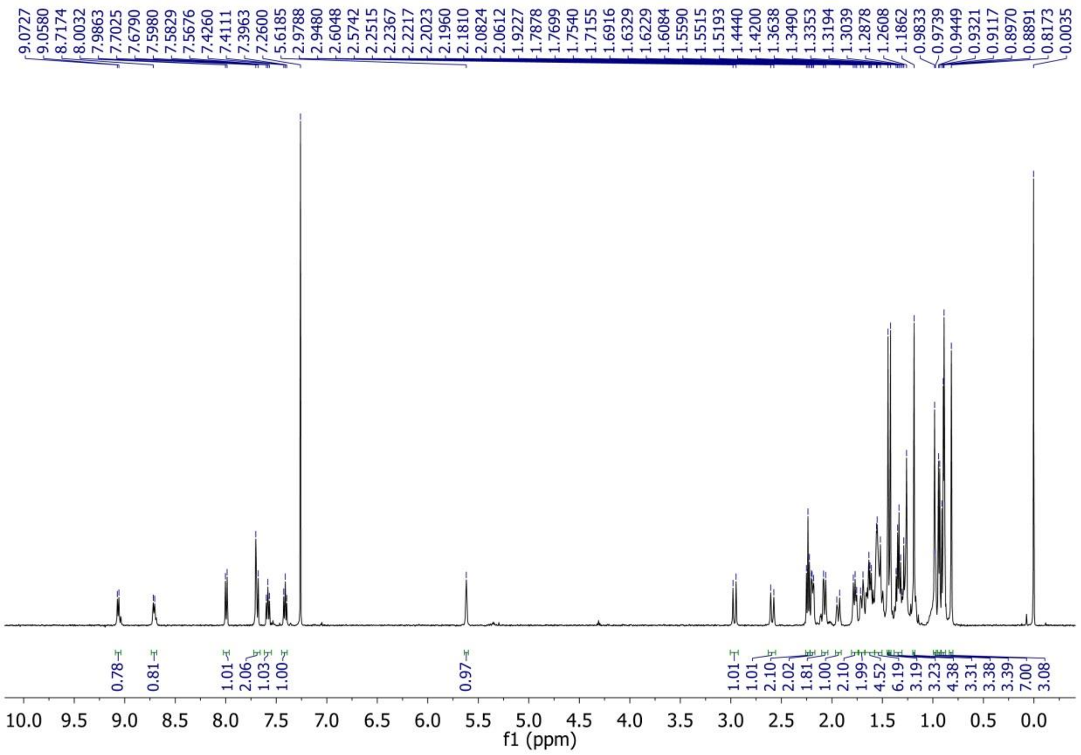

Fig. S9. ^{1}H-NMR spectrum of compound **4e** (500 MHz, $CDCl_3$).

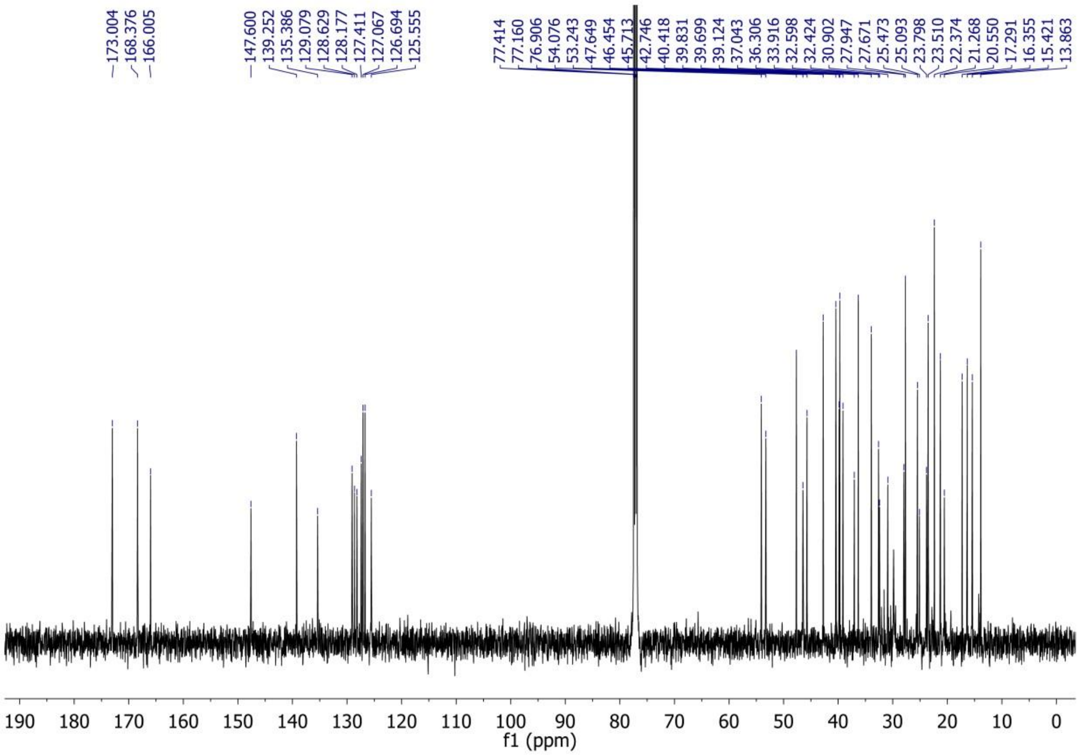

Fig. S10. ^{13}C-NMR spectrum of compound **4e** (125 MHz, $CDCl_3$).

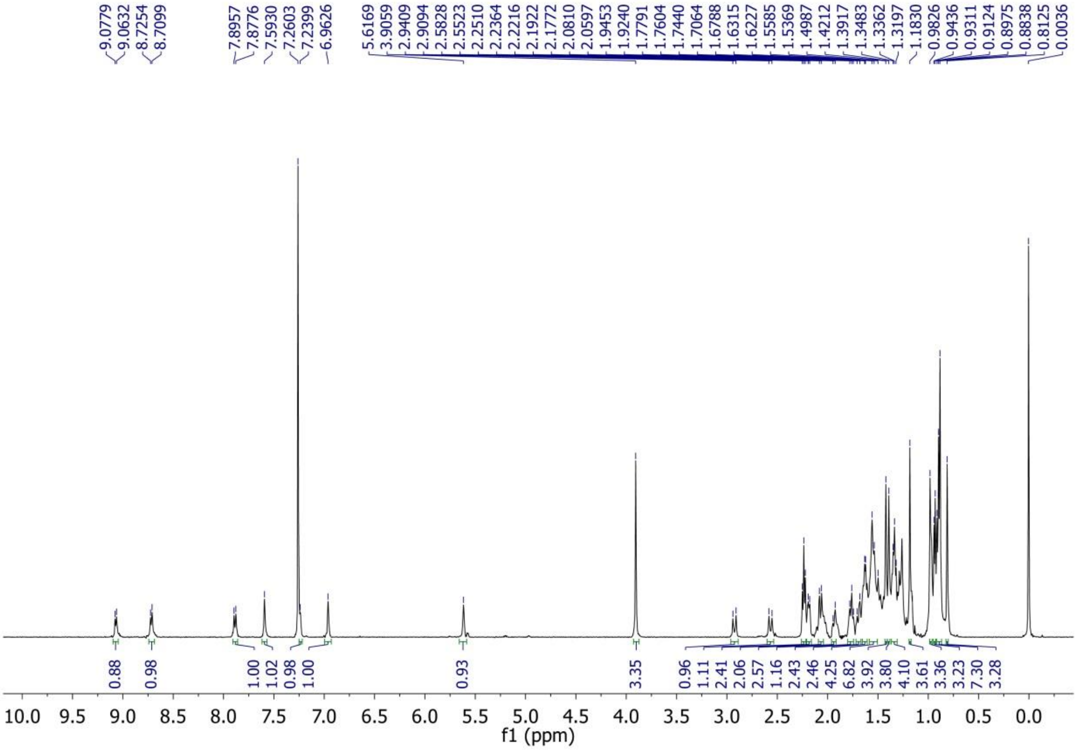

Fig. S11. ^{1}H-NMR spectrum of compound **4f** (500 MHz, $CDCl_3$).

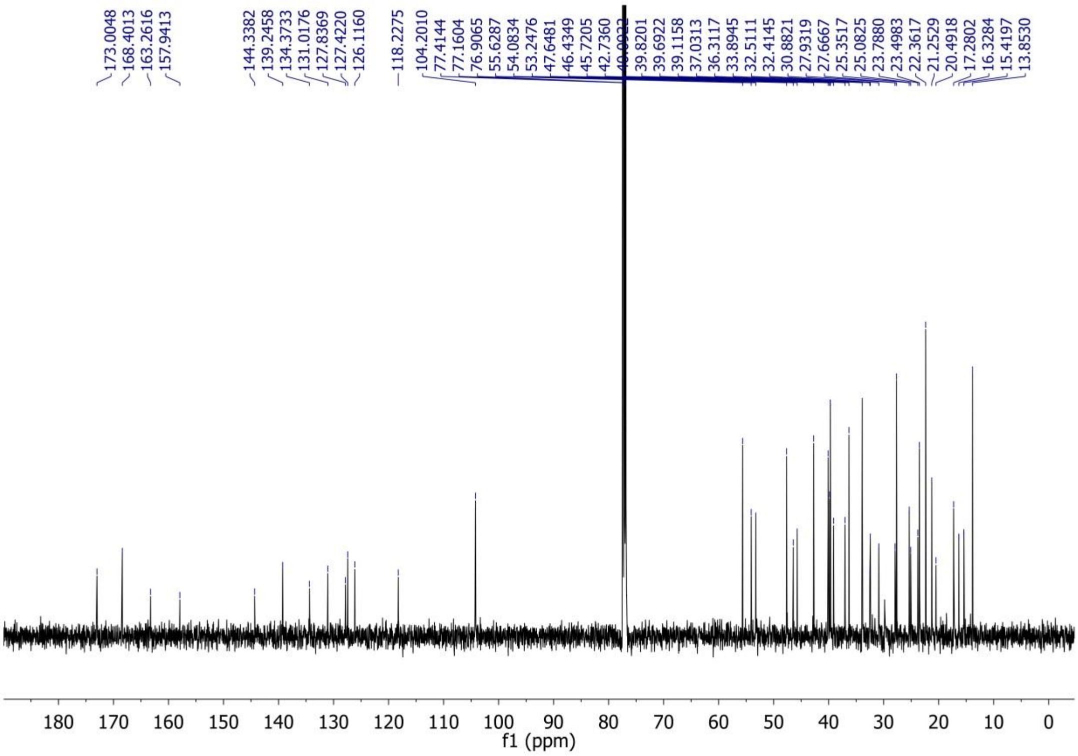

Fig. S12. ^{13}C-NMR spectrum of compound **4f** (125 MHz, $CDCl_3$).

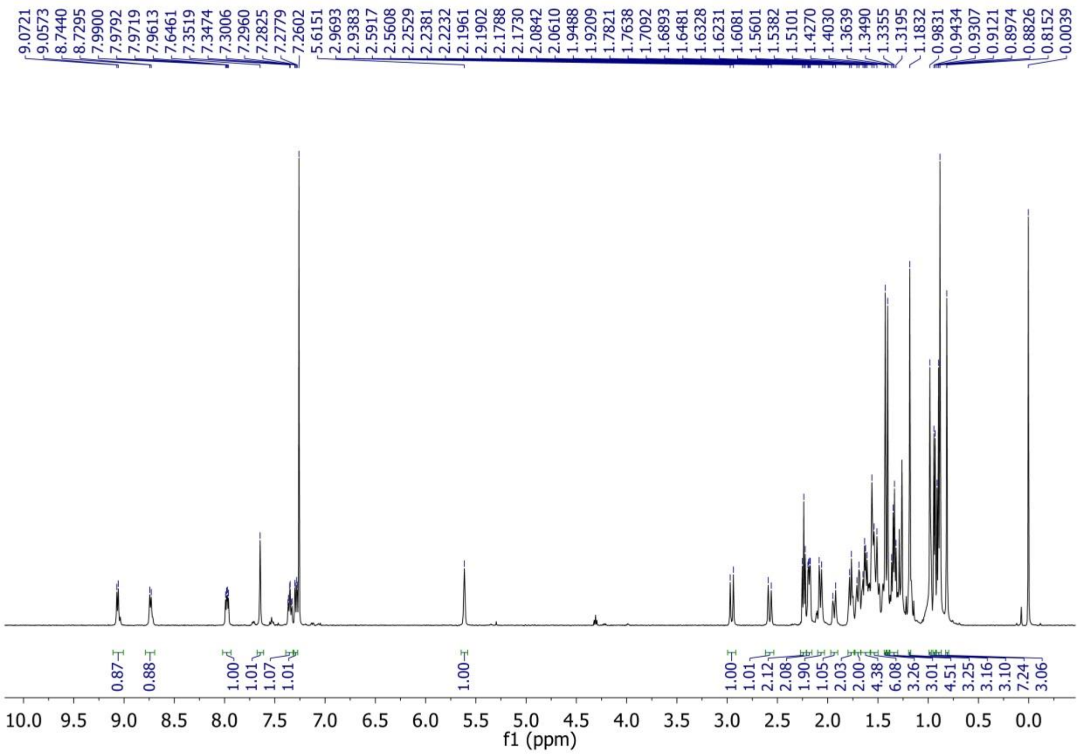

Fig. S13. ^{1}H-NMR spectrum of compound **4g** (500 MHz, $CDCl_3$).

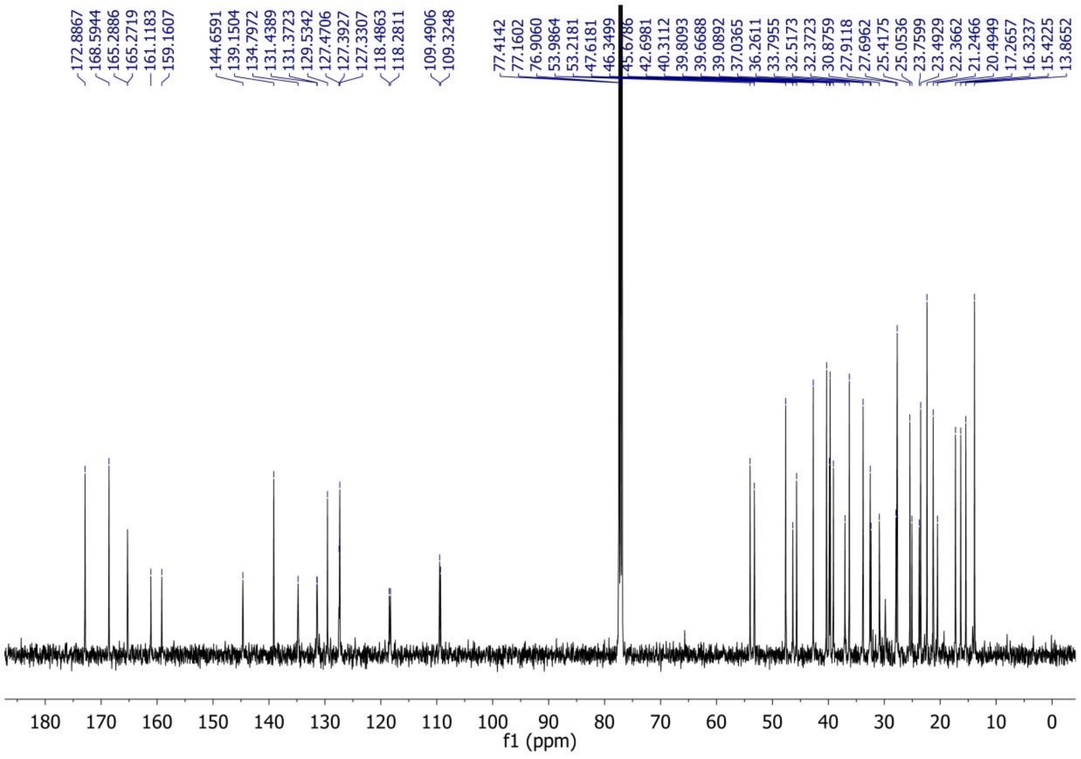

Fig. S14. ^{13}C-NMR spectrum of compound **4g** (125 MHz, $CDCl_3$).

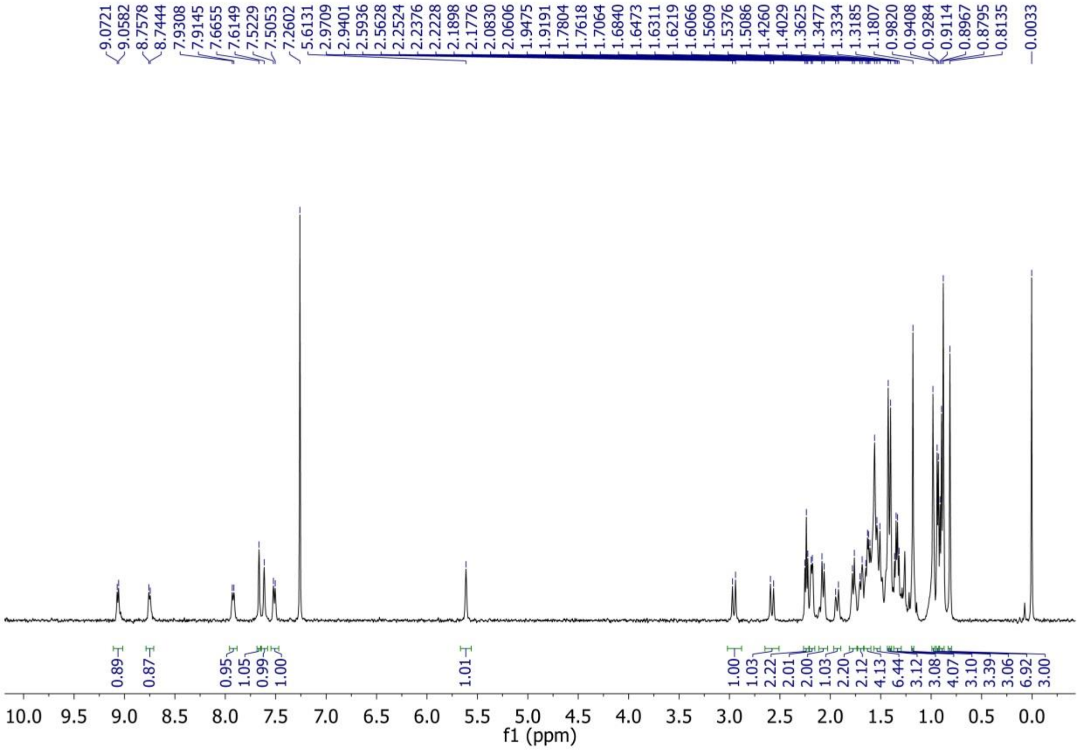

Fig. S15. ^{1}H-NMR spectrum of compound **4h** (500 MHz, $CDCl_3$).

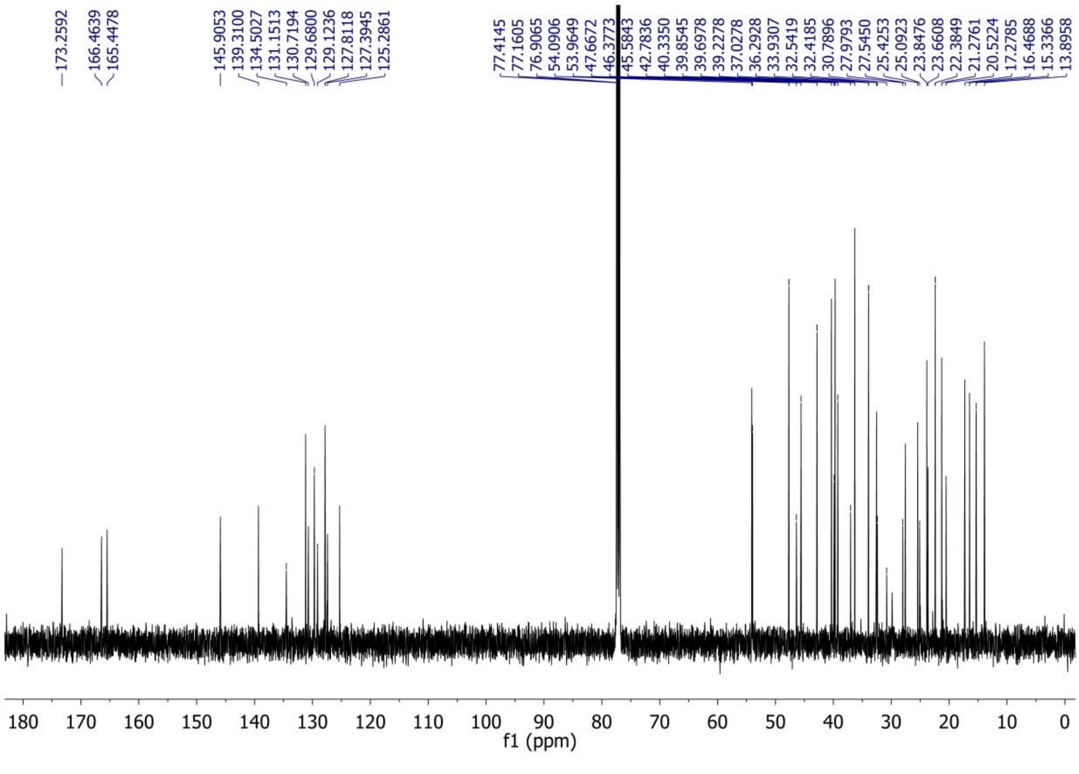

Fig. S16. ^{13}C-NMR spectrum of compound **4h** (125 MHz, $CDCl_3$).

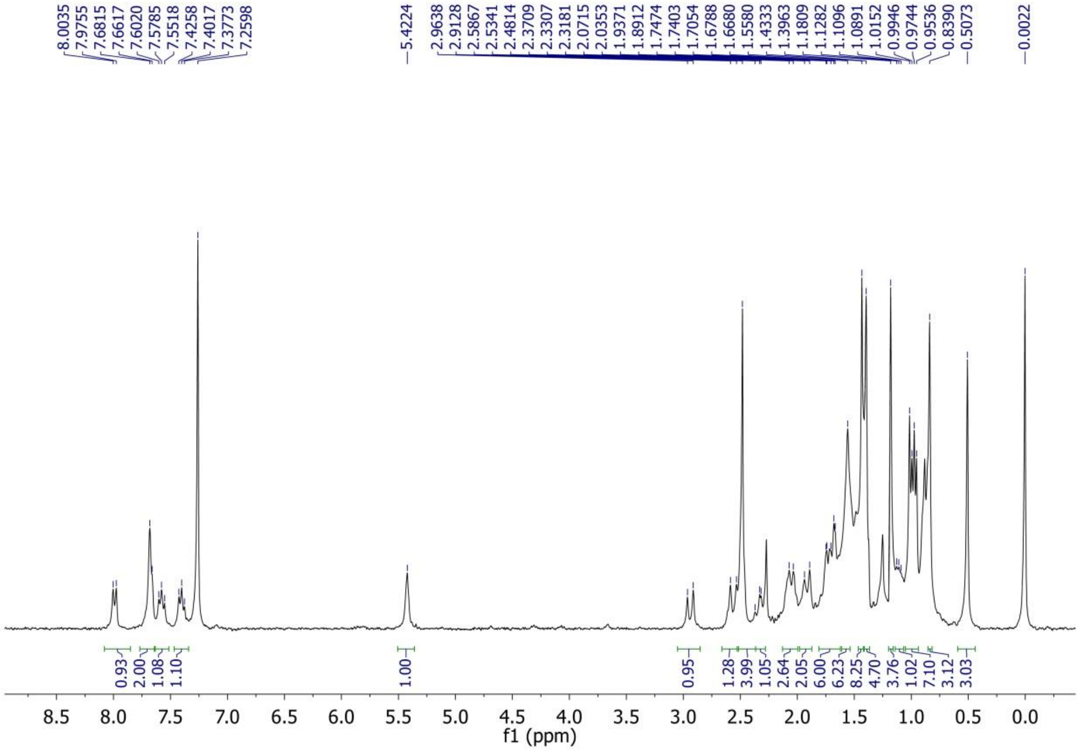

Fig. S17. ^{1}H-NMR spectrum of compound **5a** (300 MHz, $CDCl_3$).

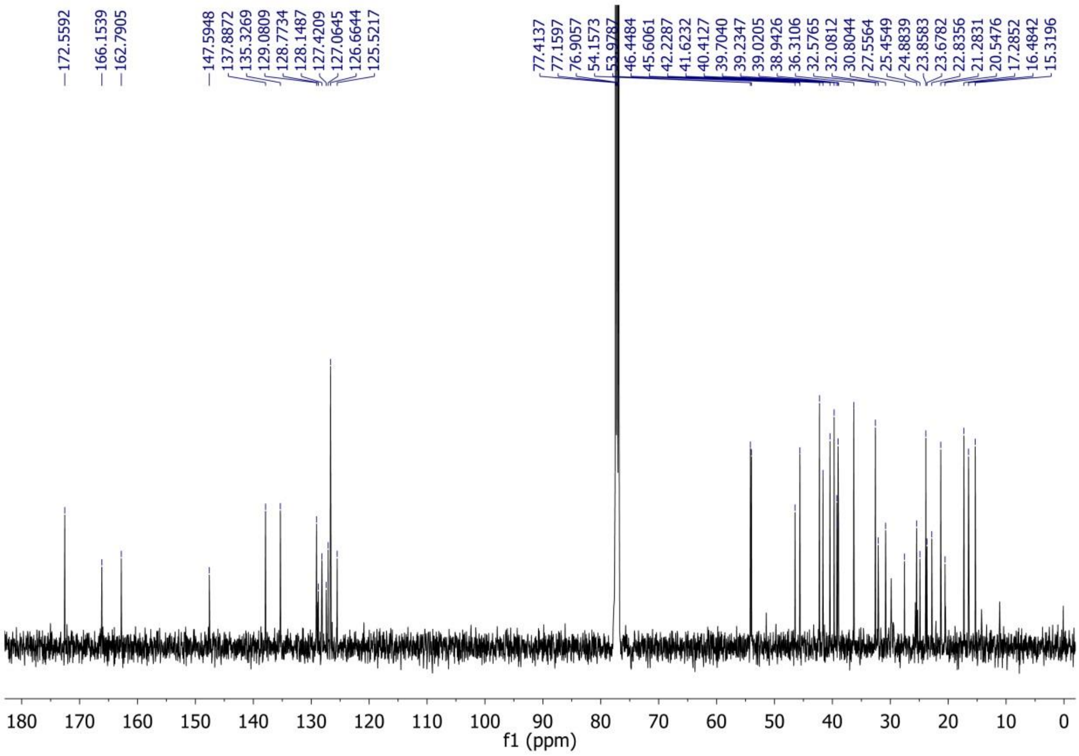

Fig. S18. ^{13}C-NMR spectrum of compound **5a** (125 MHz, $CDCl_3$).

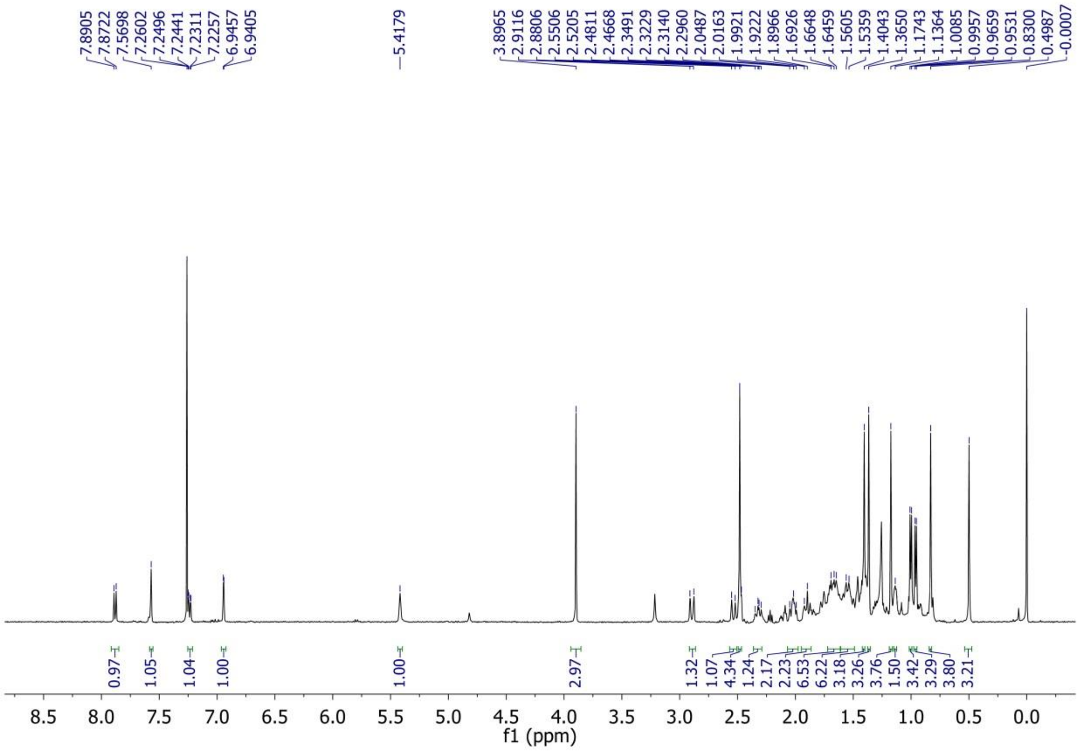

Fig. S19. ^{1}H-NMR spectrum of compound **5b** (500 MHz, $CDCl_3$).

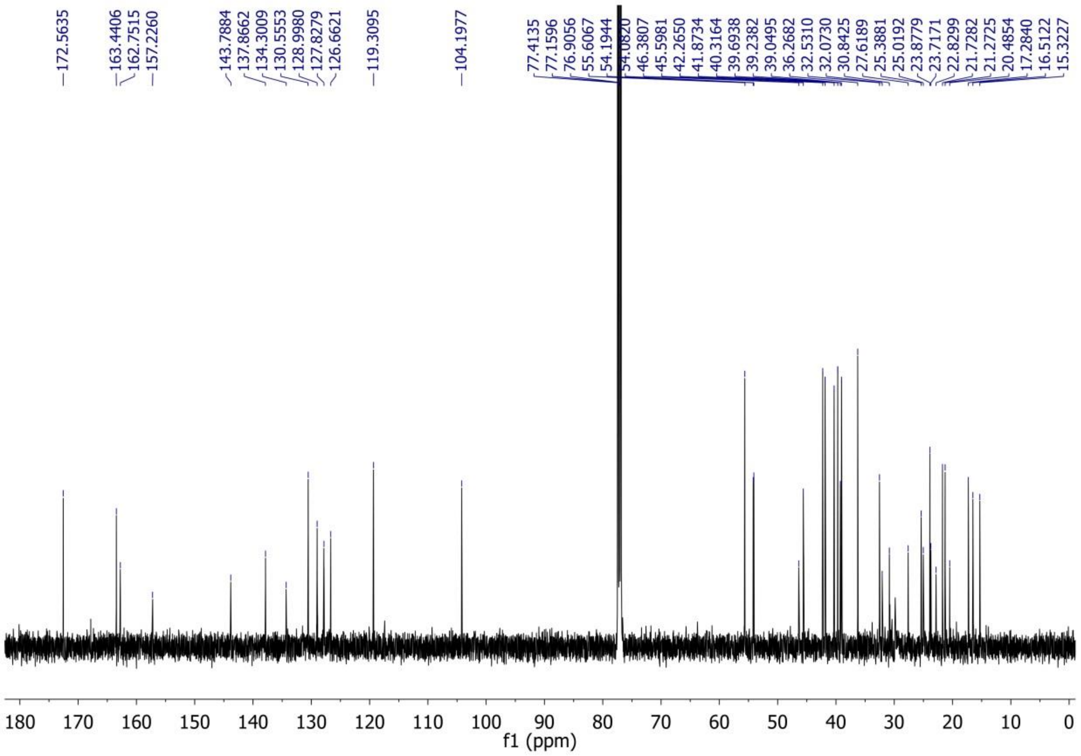

Fig. S20. ^{13}C-NMR spectrum of compound **5b** (125 MHz, $CDCl_3$).

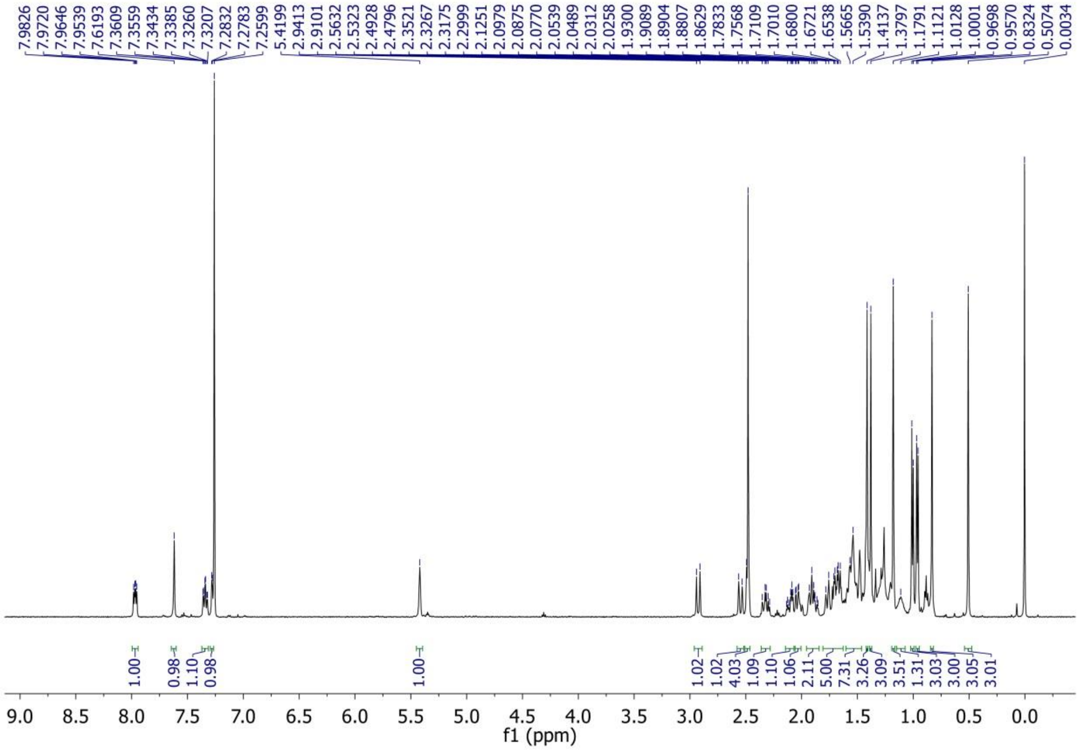

Fig. S21. ^{1}H-NMR spectrum of compound **5c** (500 MHz, $CDCl_3$).

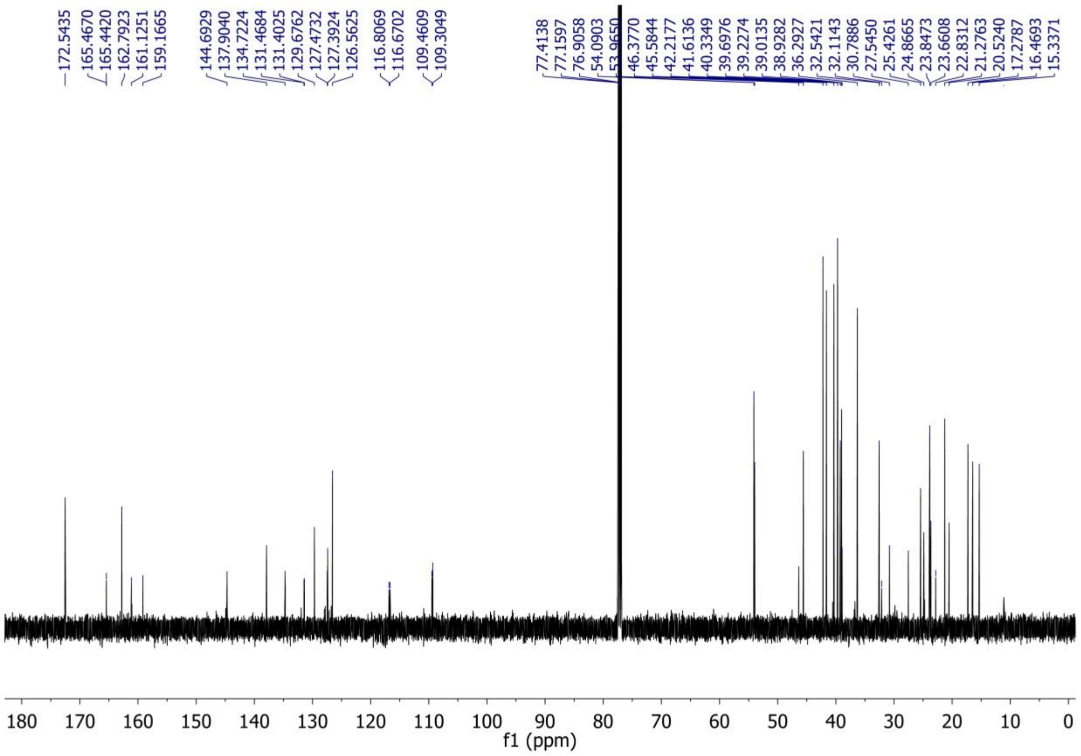

Fig. S22. ^{13}C-NMR spectrum of compound **5c** (125 MHz, $CDCl_3$).

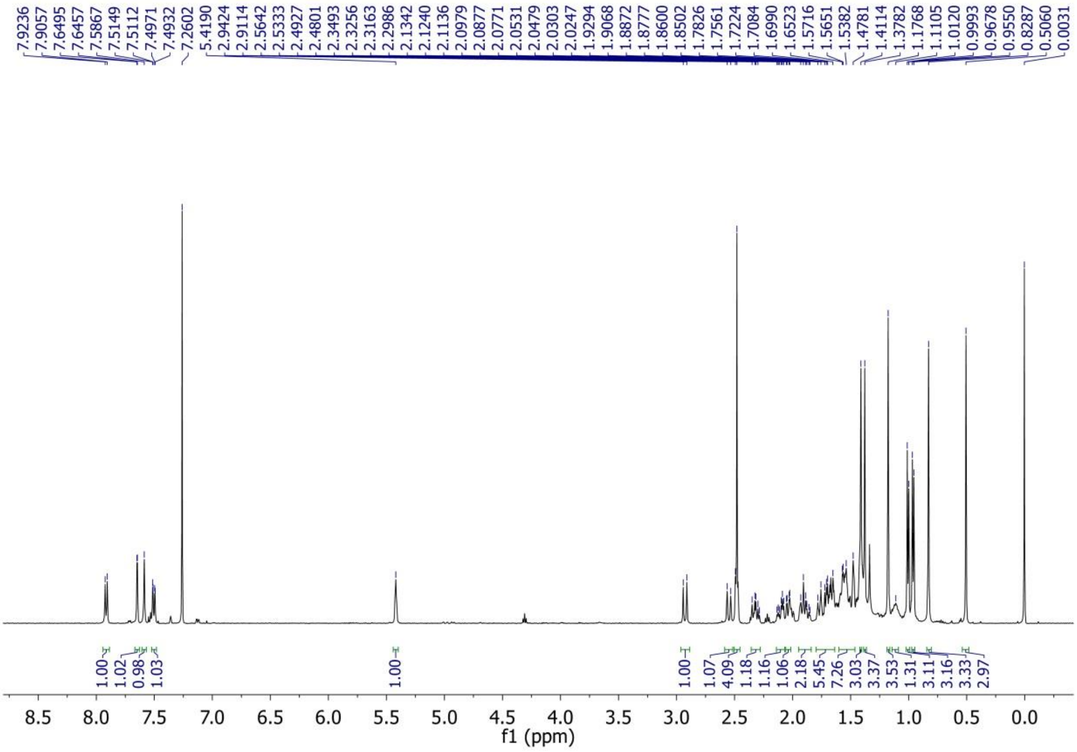

Fig. S23. ^{1}H-NMR spectrum of compound **5d** (500 MHz, $CDCl_3$).

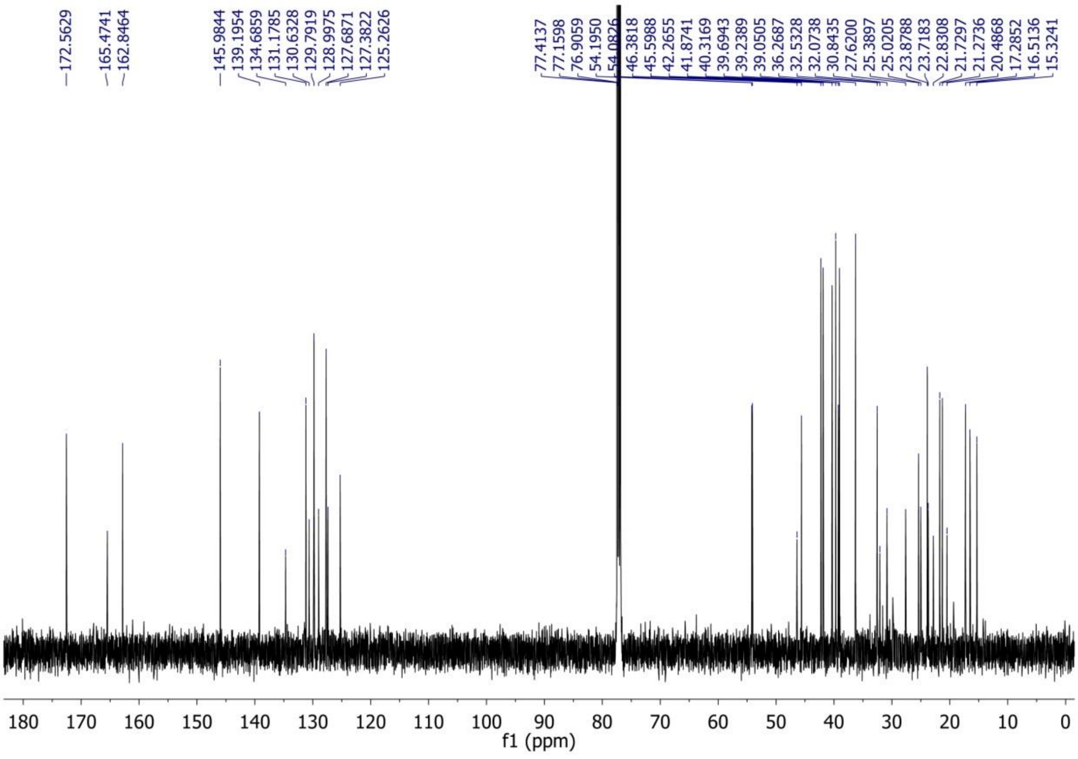

Fig. S24. ^{13}C-NMR spectrum of compound **5d** (125 MHz, $CDCl_3$).

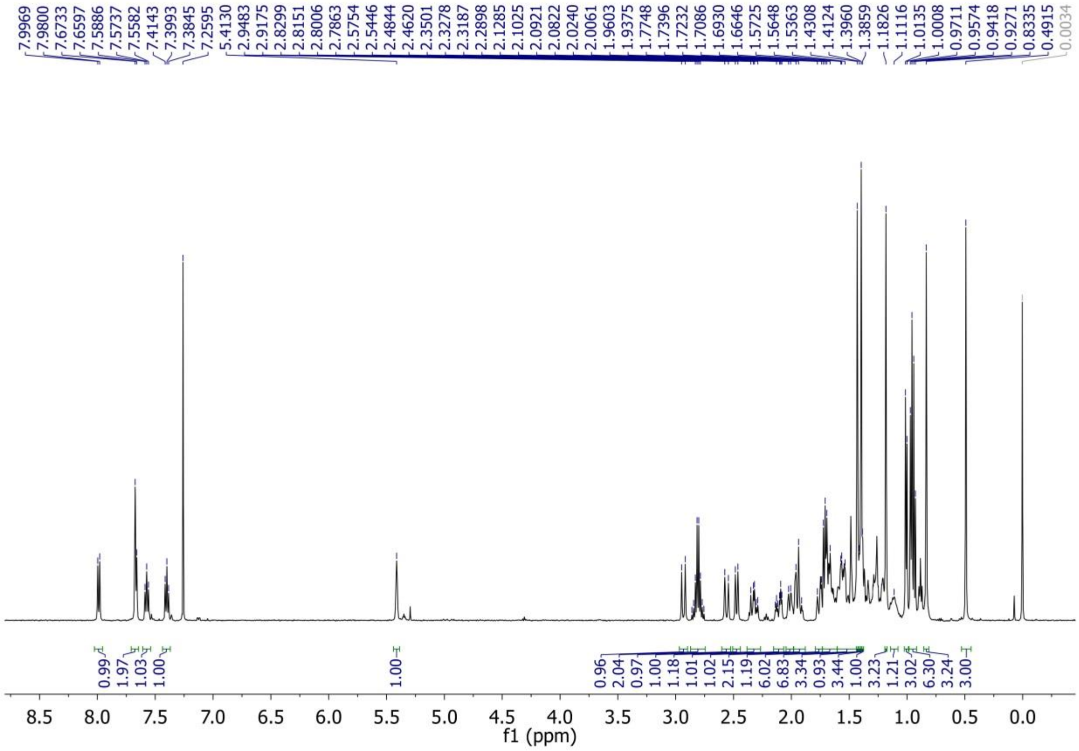

Fig. S25. ^{1}H-NMR spectrum of compound **5e** (500 MHz, $CDCl_3$).

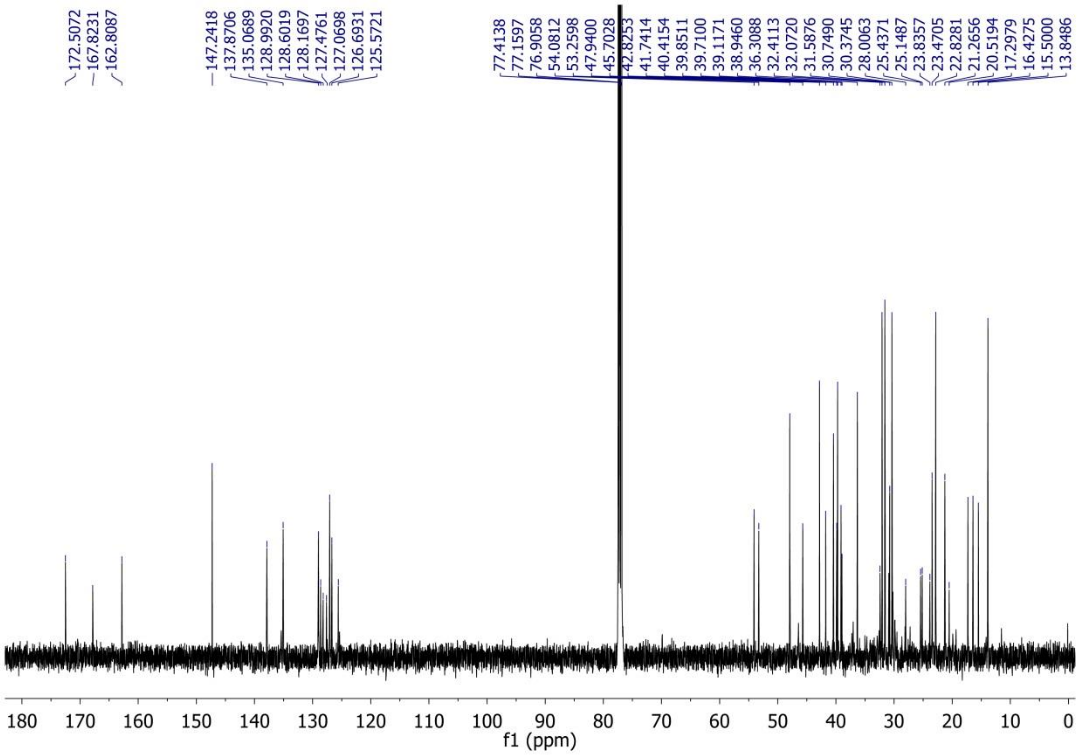

Fig. S26. ^{13}C-NMR spectrum of compound **5e** (125 MHz, $CDCl_3$).

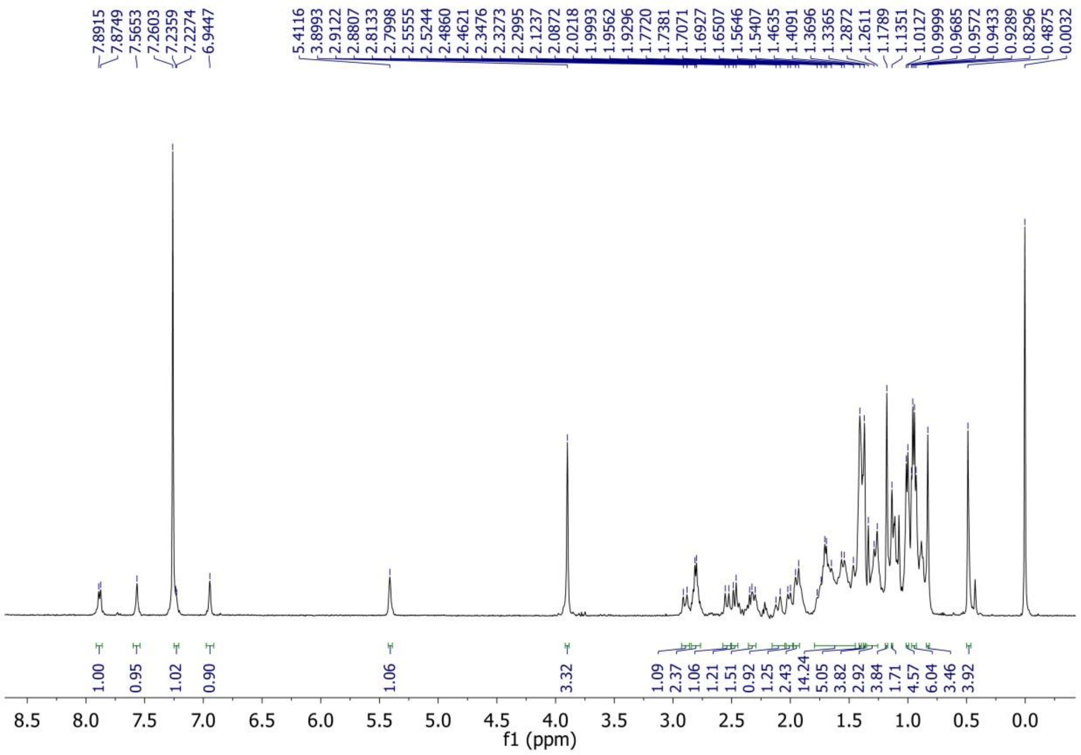

Fig. S27. ^{1}H-NMR spectrum of compound **5f** (500 MHz, $CDCl_3$).

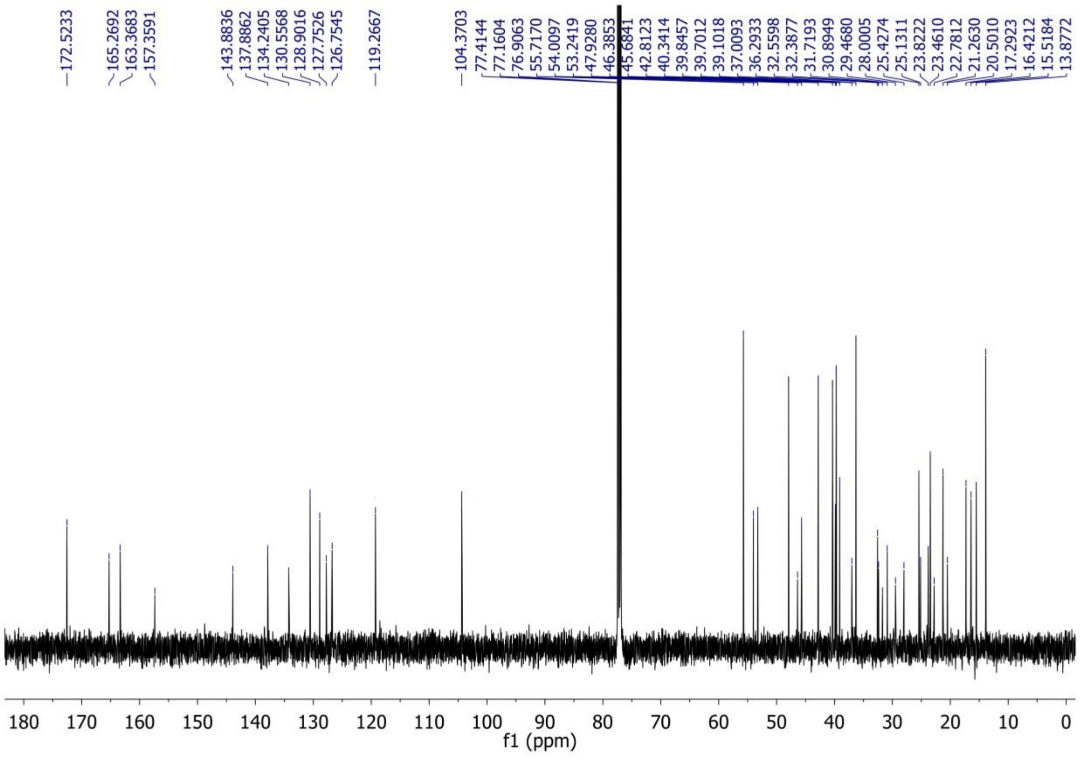

Fig. S28. ^{13}C-NMR spectrum of compound **5f** (125 MHz, $CDCl_3$).

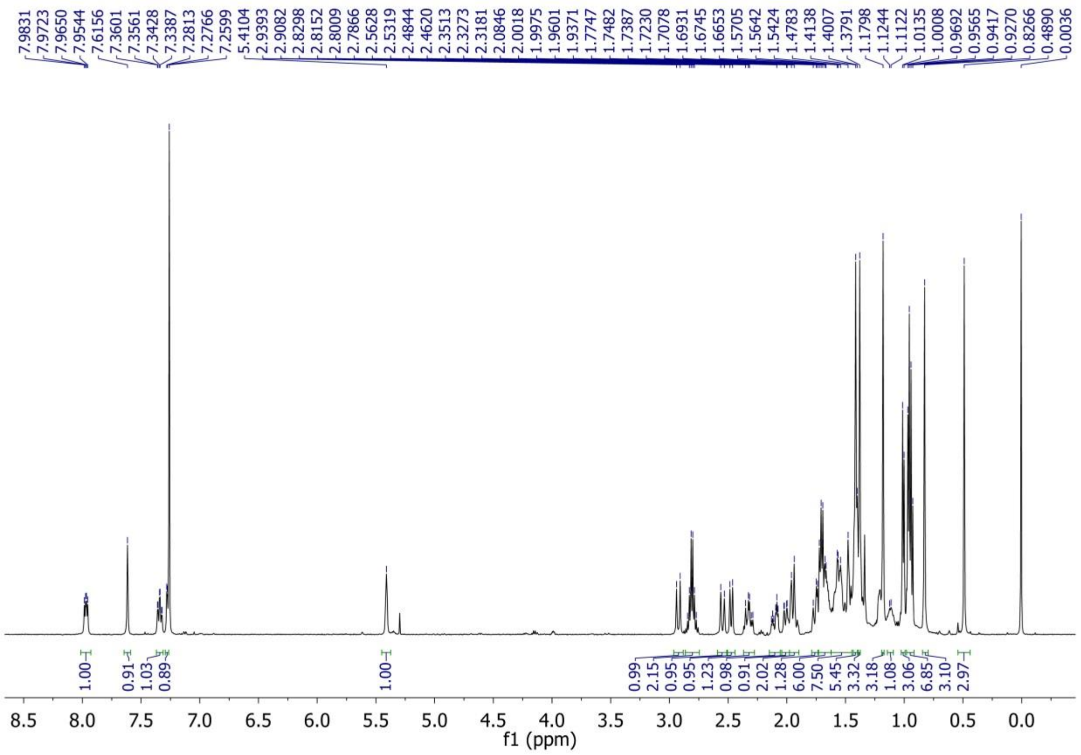

Fig. S29. ^{1}H-NMR spectrum of compound **5g** (500 MHz, $CDCl_3$).

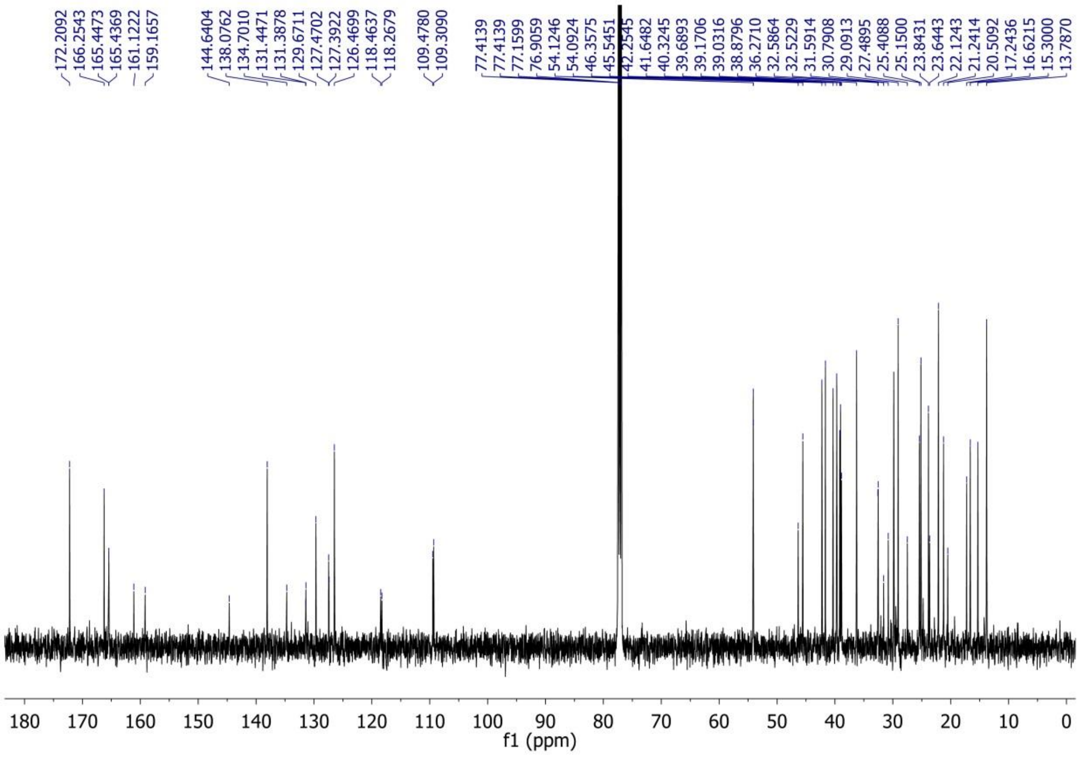

Fig. S30. ^{13}C-NMR spectrum of compound **5g** (125 MHz, $CDCl_3$).

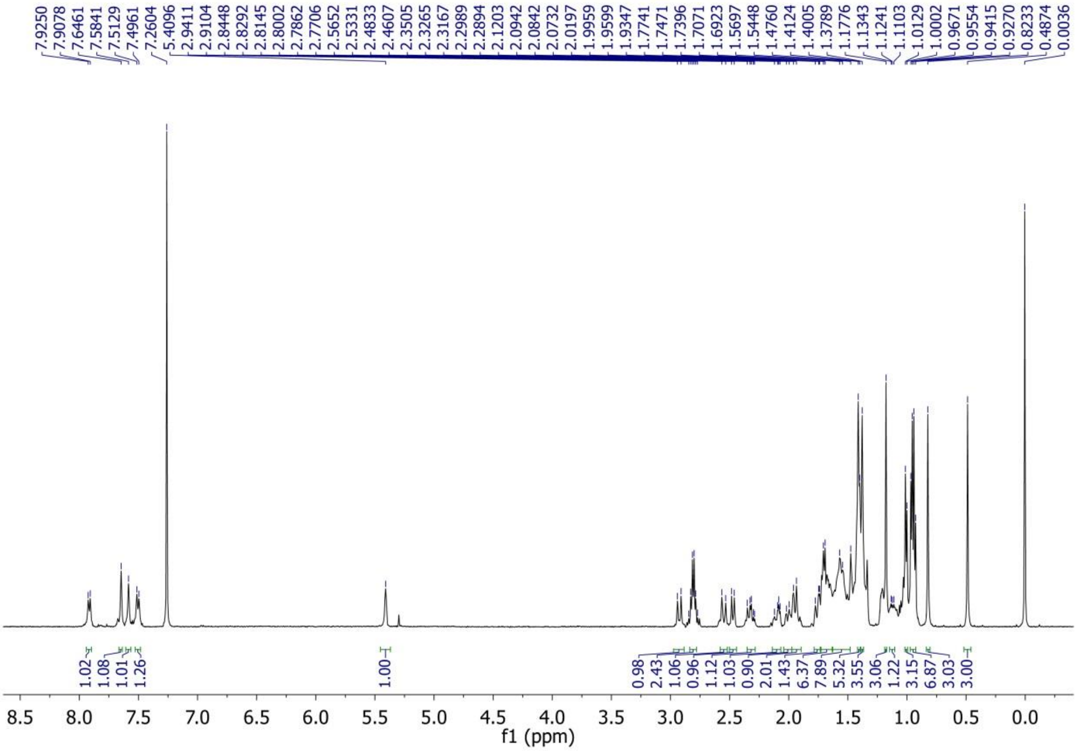

Fig. S31. ^{1}H-NMR spectrum of compound **5h** (500 MHz, $CDCl_3$).

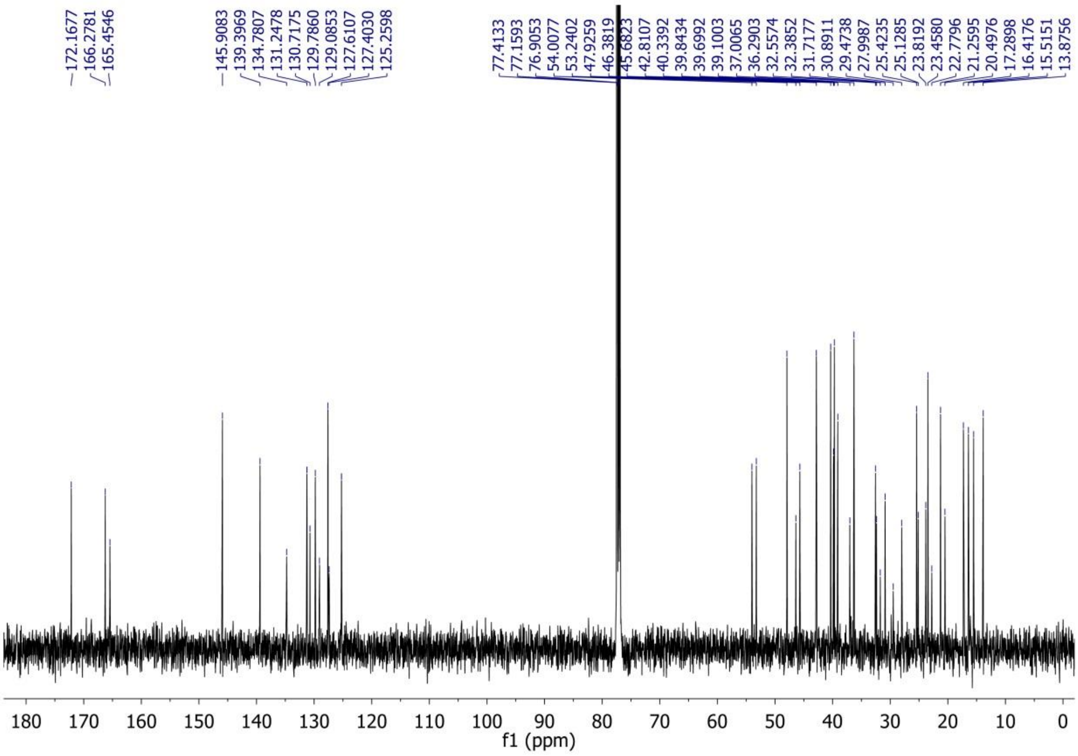

Fig. S32. ^{13}C-NMR spectrum of compound **5h** (125 MHz, $CDCl_3$).

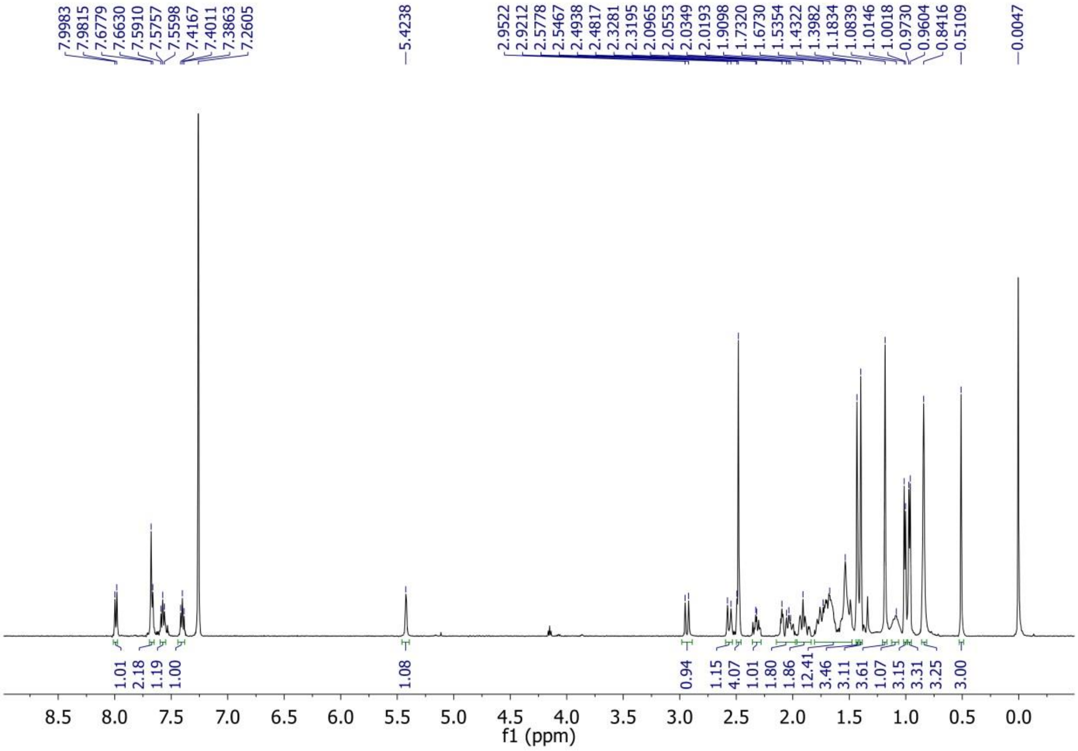

Fig. S33. ^{1}H-NMR spectrum of compound **6a** (500 MHz, $CDCl_3$).

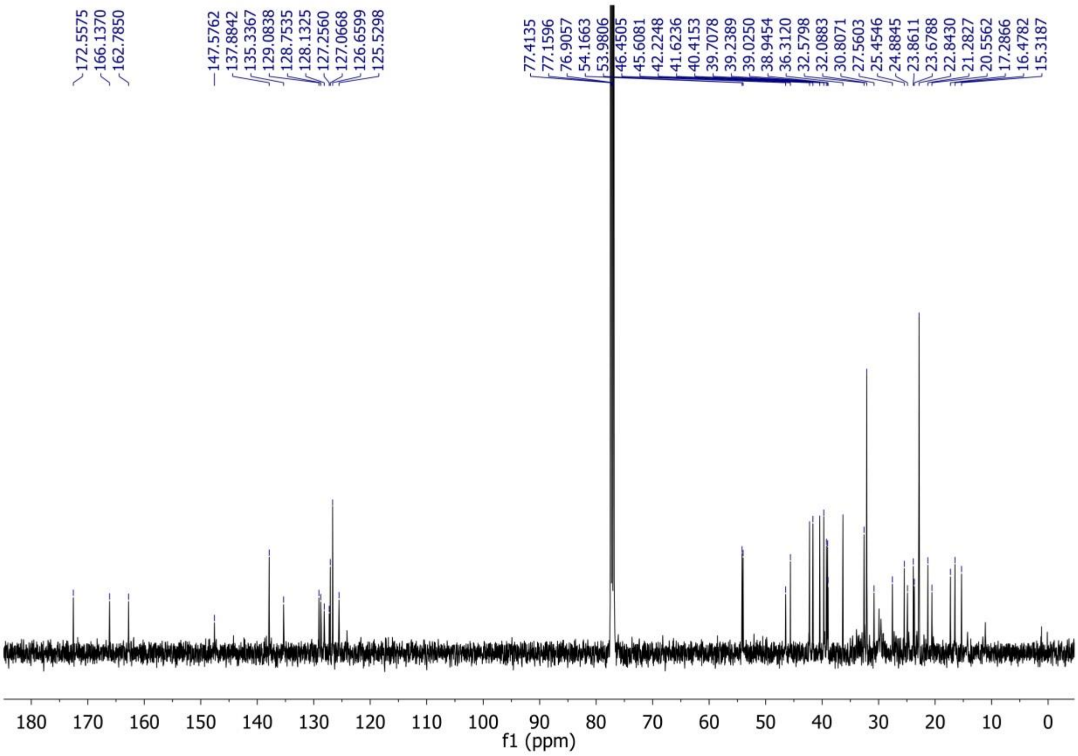

Fig. S34. ^{13}C-NMR spectrum of compound **6a** (125 MHz, $CDCl_3$).

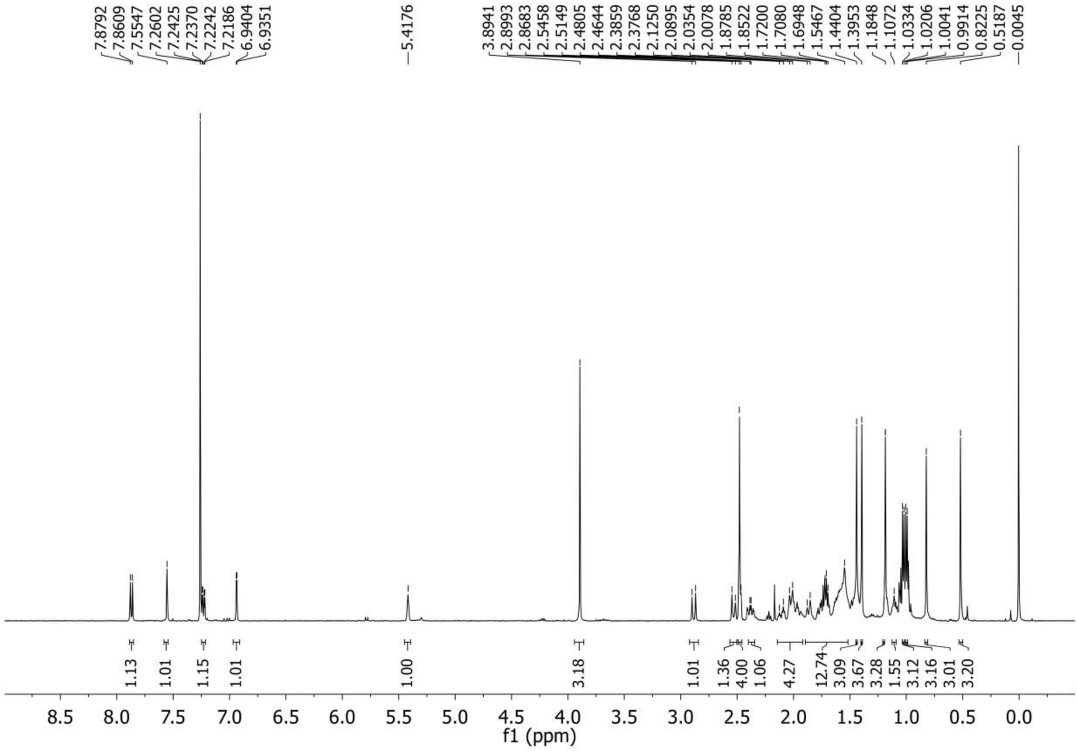

Fig. S35. ^{1}H-NMR spectrum of compound **6b** (500 MHz, $CDCl_3$).

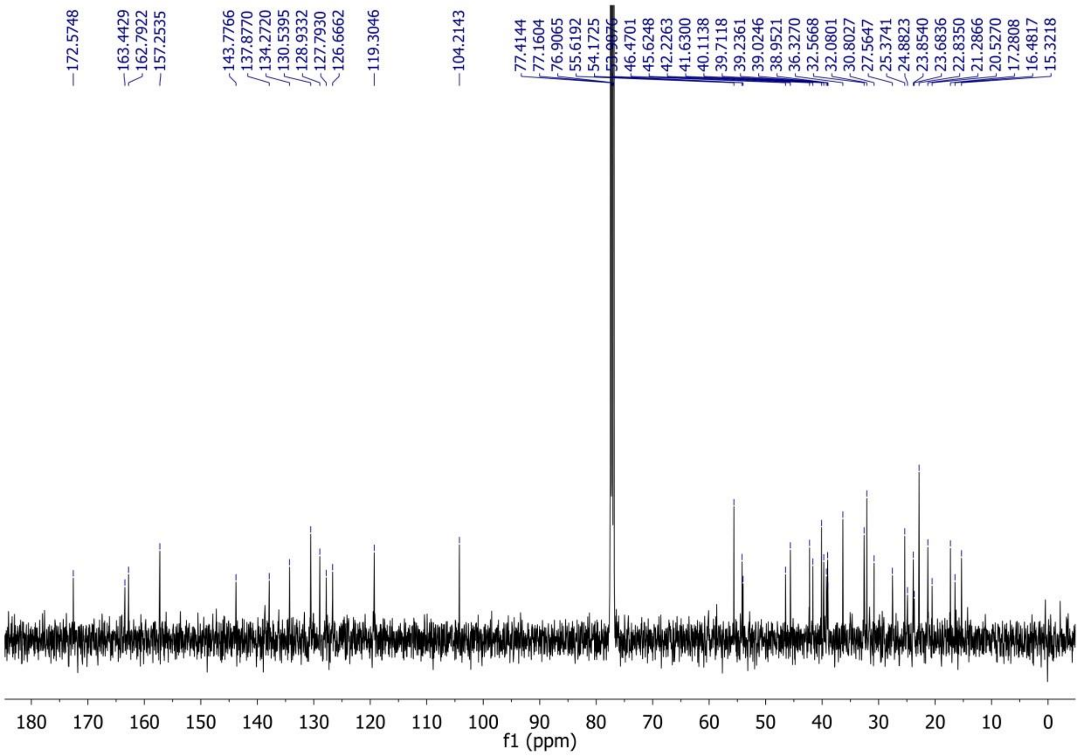

Fig. S36. ^{13}C-NMR spectrum of compound **6b** (125 MHz, $CDCl_3$).

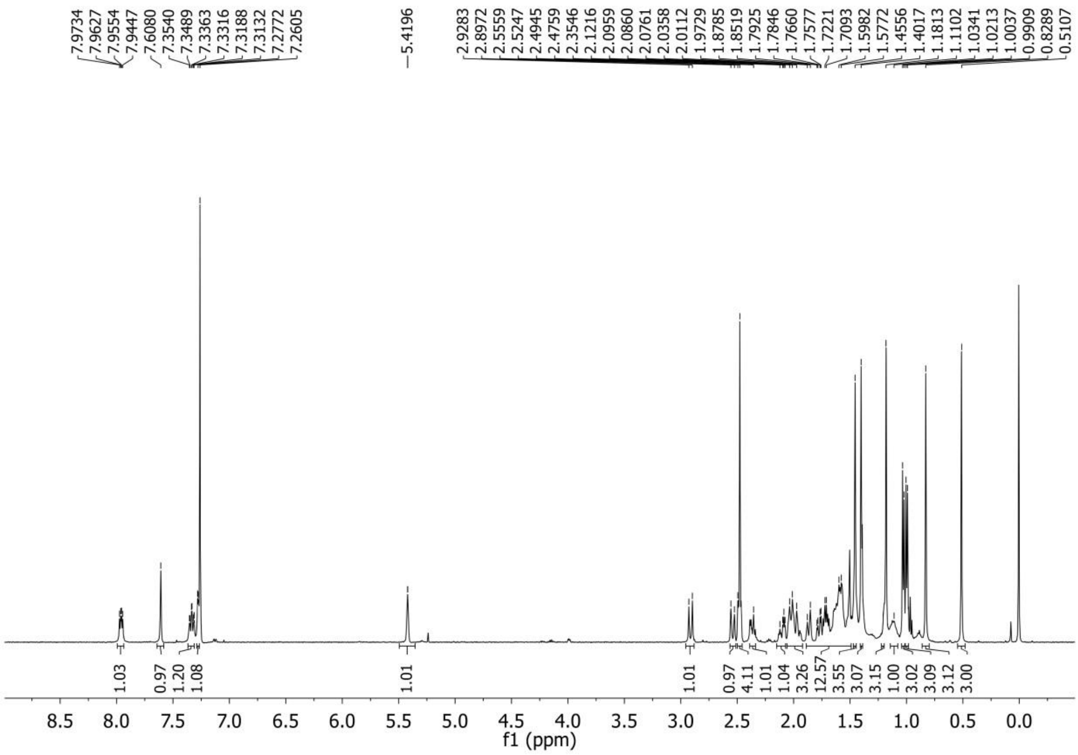

Fig. S37. ^{1}H-NMR spectrum of compound **6c** (500 MHz, $CDCl_3$).

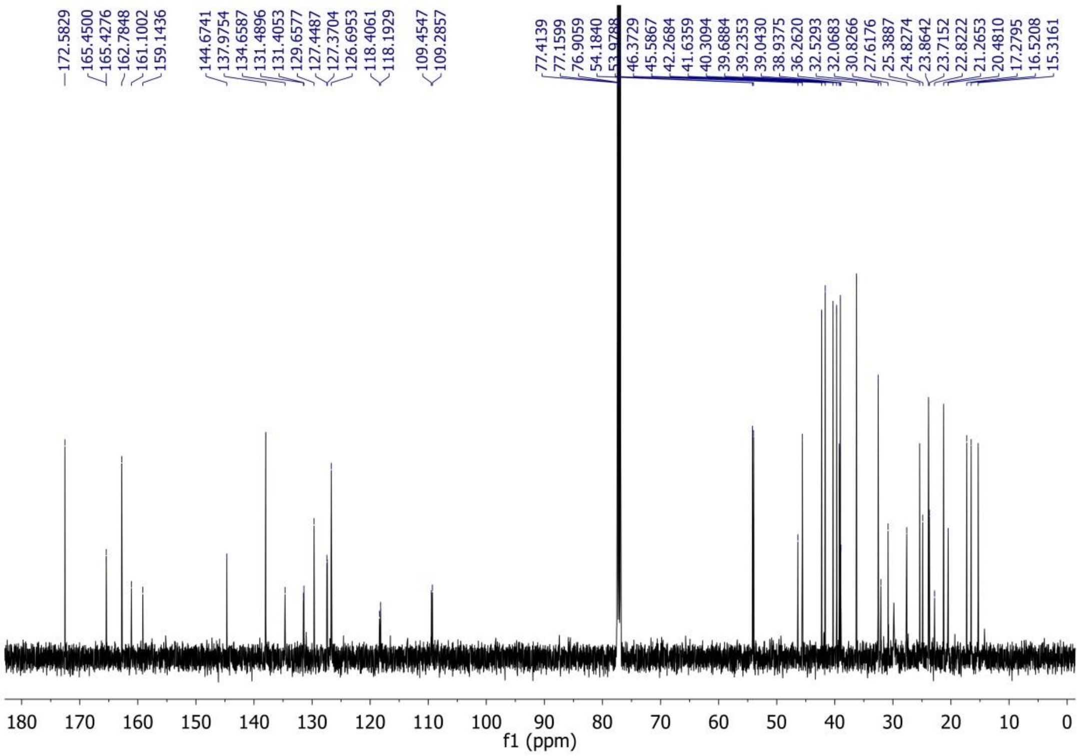

Fig. S38. ^{13}C-NMR spectrum of compound **6c** (125 MHz, $CDCl_3$).

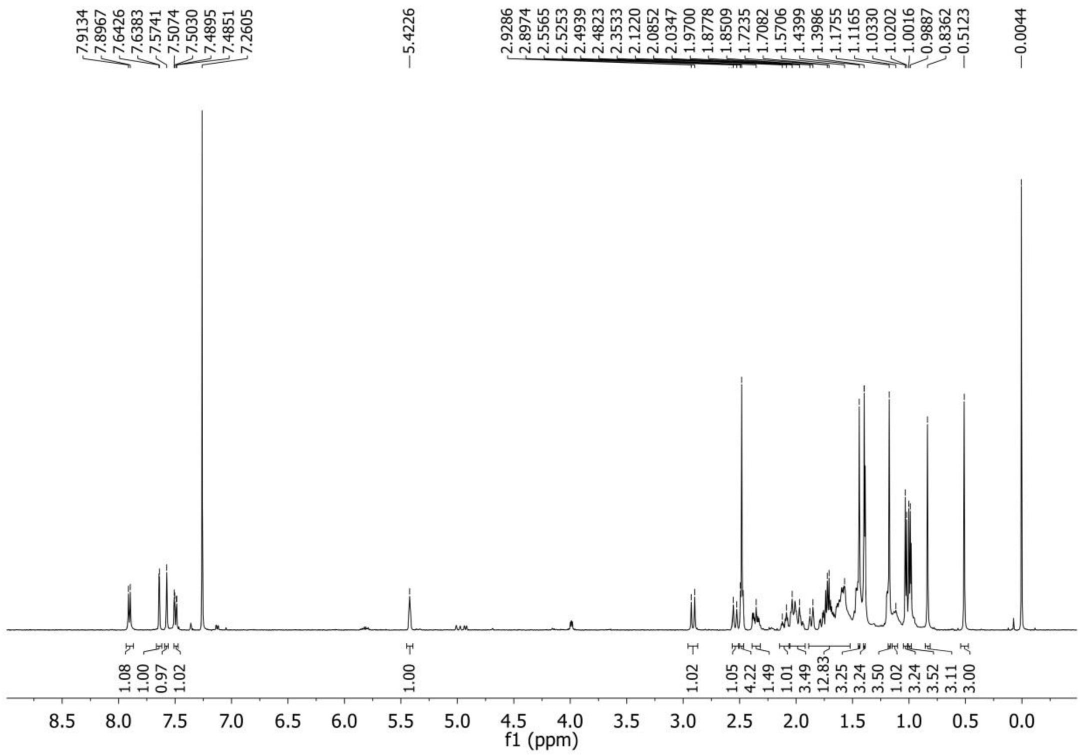

Fig. S39. ^{1}H-NMR spectrum of compound **6d** (500 MHz, $CDCl_3$).

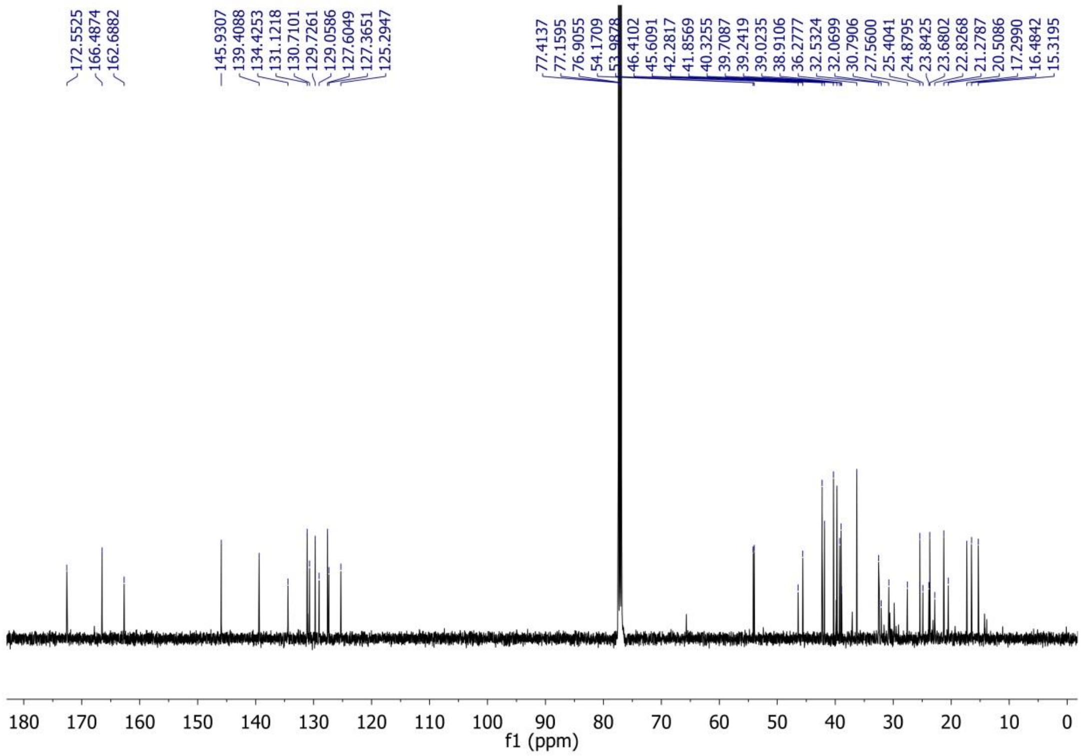

Fig. S40. ^{13}C-NMR spectrum of compound **6d** (125 MHz, $CDCl_3$).

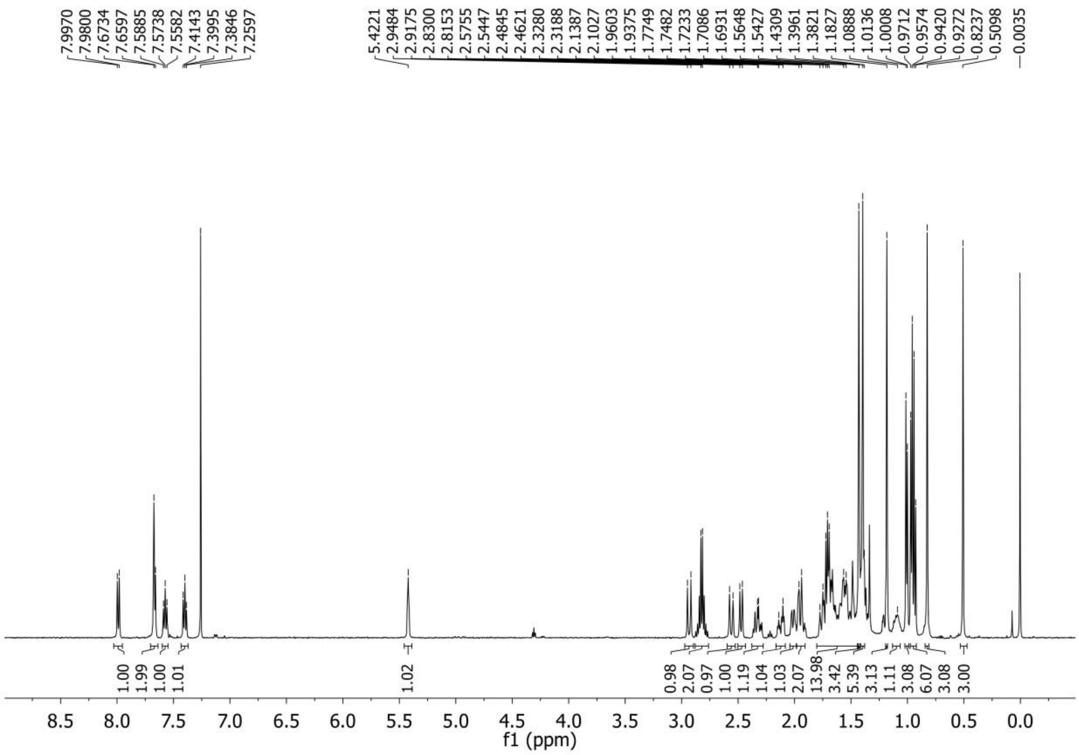

Fig. S41. ^{1}H-NMR spectrum of compound **6e** (500 MHz, $CDCl_3$).

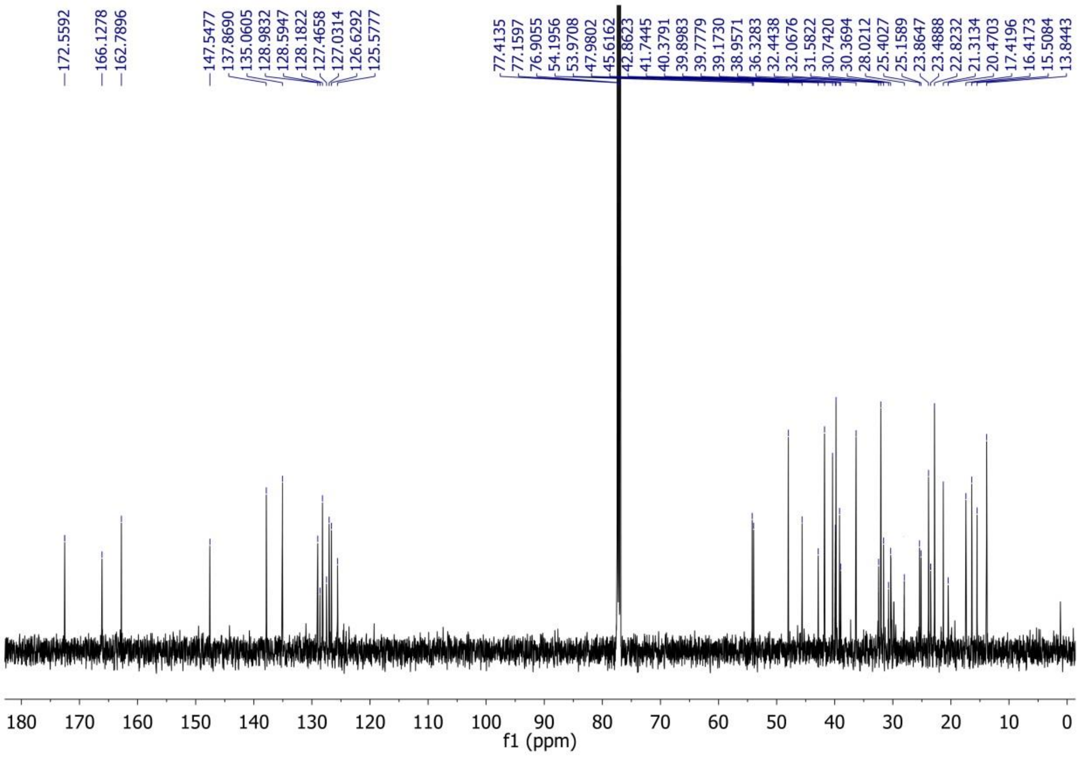

Fig. S42. ^{13}C-NMR spectrum of compound **6e** (125 MHz, $CDCl_3$).

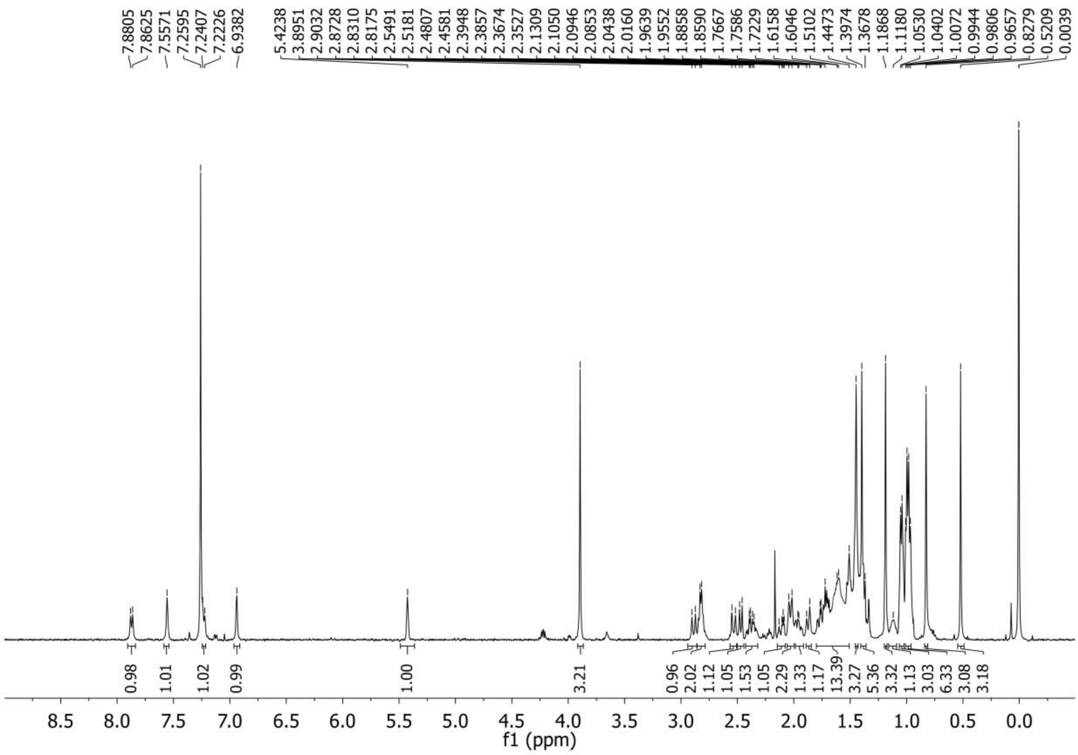

Fig. S43. ^{1}H-NMR spectrum of compound **6f** (500 MHz, $CDCl_3$).

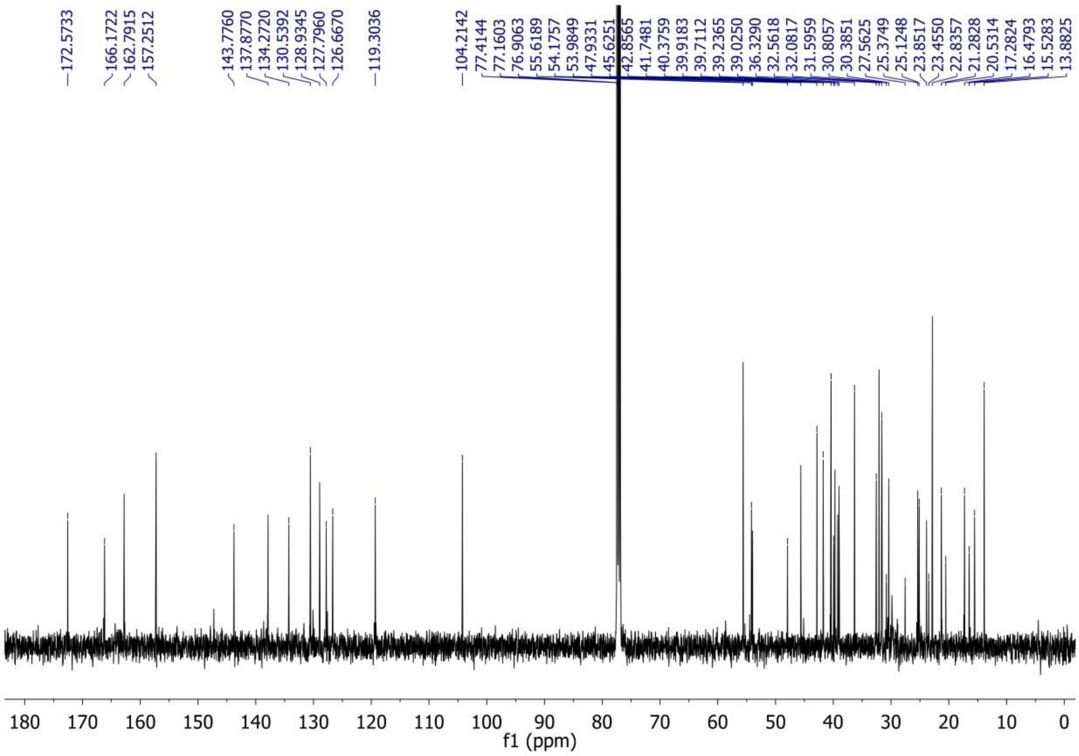

Fig. S44. ^{13}C-NMR spectrum of compound **6f** (125 MHz, $CDCl_3$).

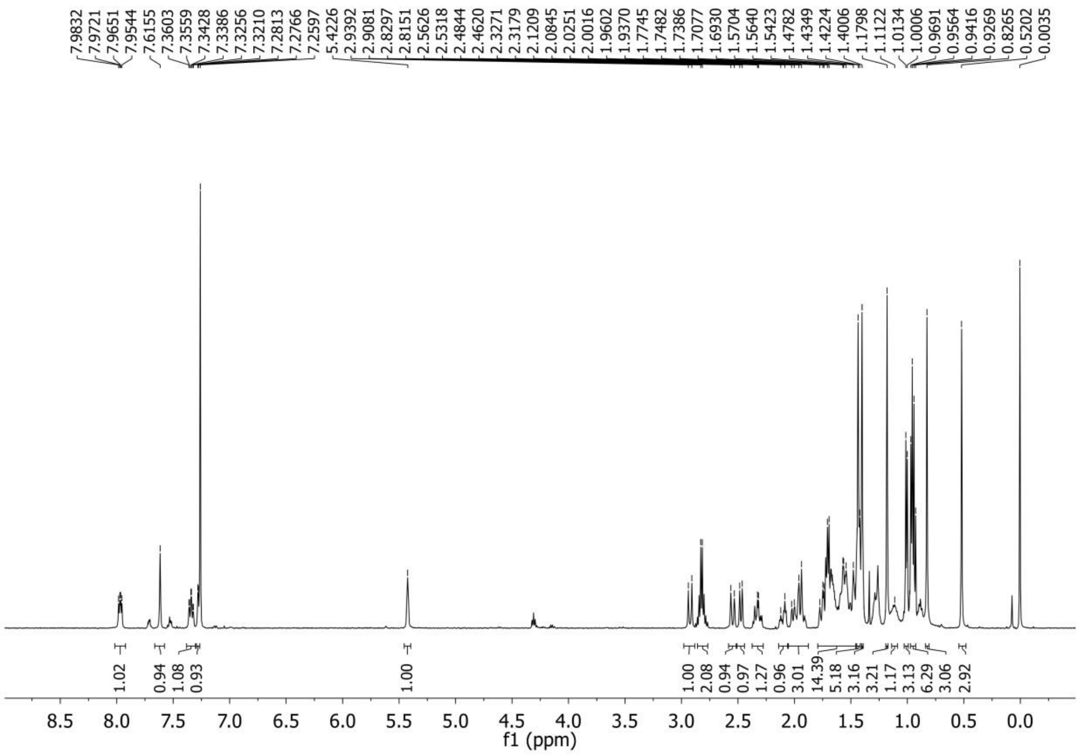

Fig. S45. ^{1}H-NMR spectrum of compound **6g** (500 MHz, $CDCl_3$).

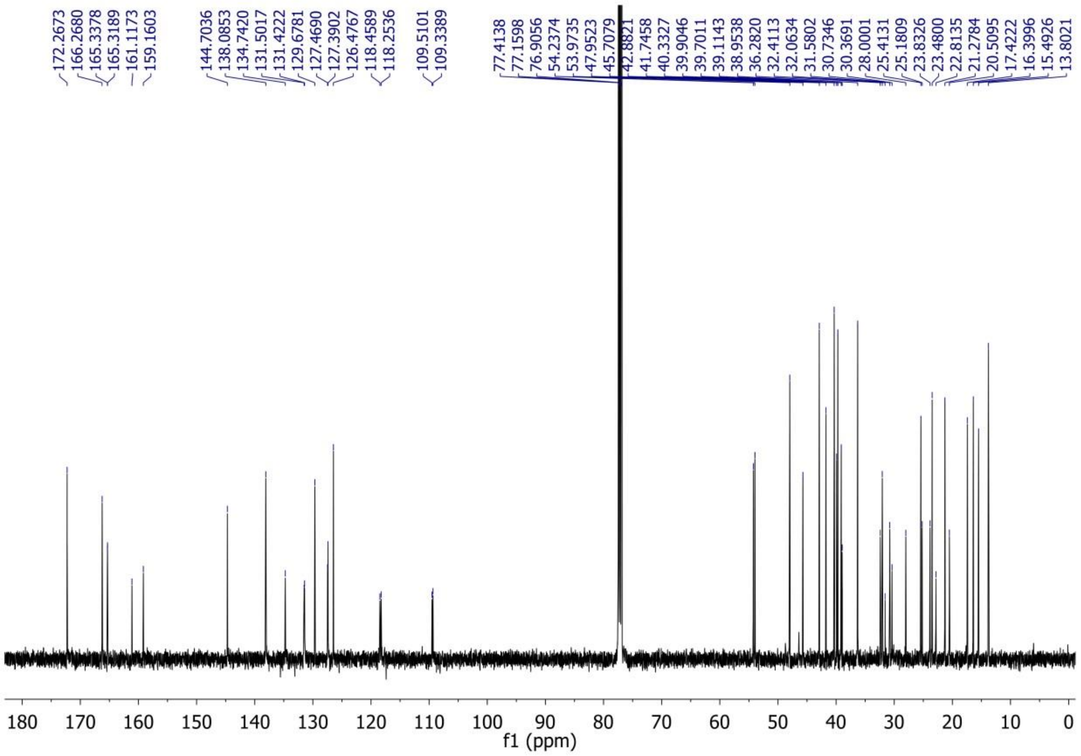

Fig. S46. ^{13}C-NMR spectrum of compound **6g** (125 MHz, $CDCl_3$).

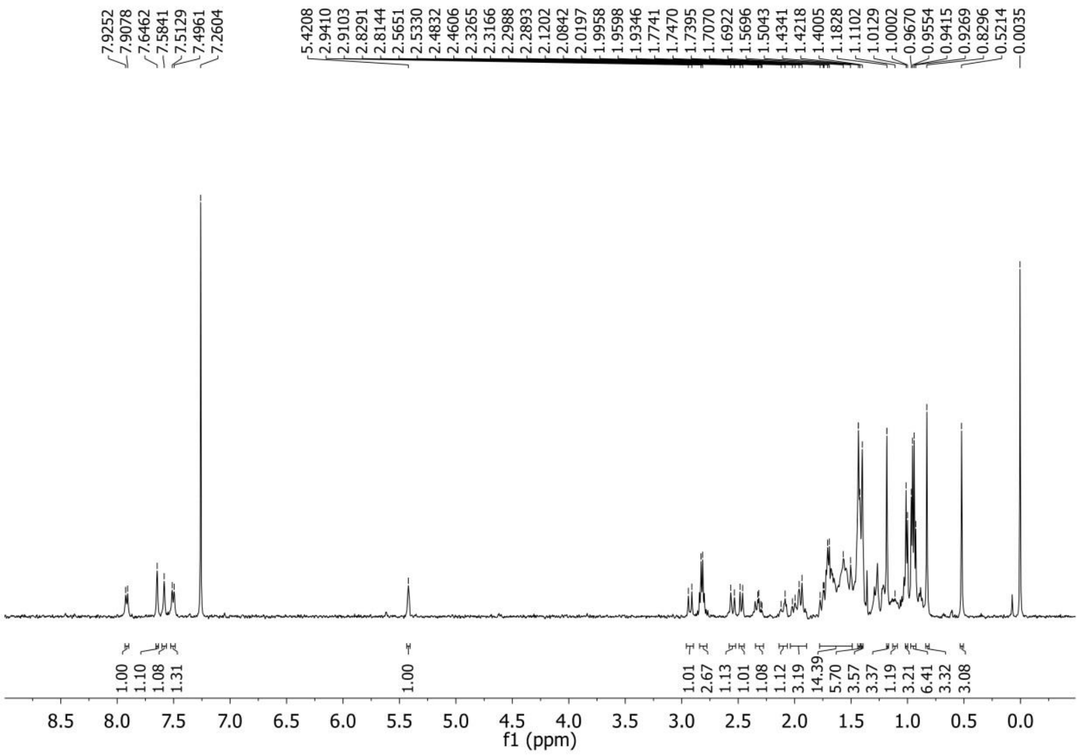

Fig. S47. ^{1}H-NMR spectrum of compound **6h** (500 MHz, $CDCl_3$).

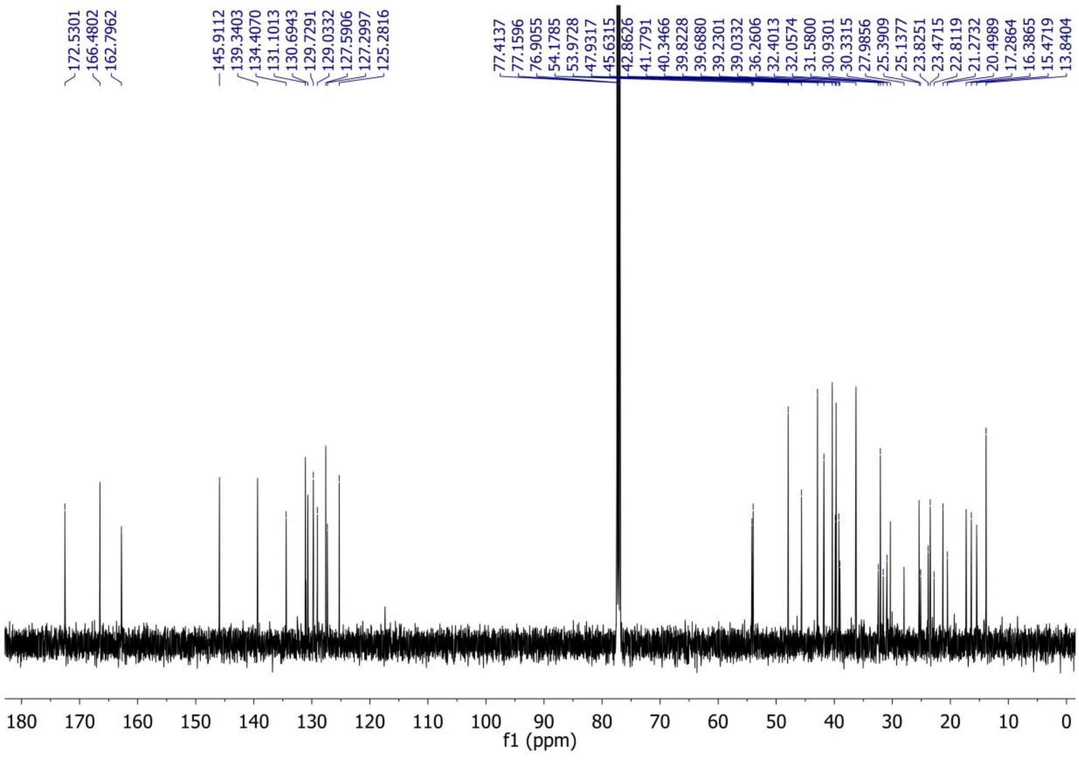

Fig. S48. ^{13}C-NMR spectrum of compound **6h** (125 MHz, $CDCl_3$).
